# Validation of new equipment for SARS-CoV-2 diagnosis in Ecuador: Detection of the virus and antibodies generated by disease and vaccines with one POC device

**DOI:** 10.1371/journal.pone.0321794

**Published:** 2025-04-16

**Authors:** Stephany D. Villota, Eliana Veloz-Villavicencio, Santiago Garcia-Iturralde, Johanna Valentina Arévalo, Suying Lu, Katariina Jaenes, Yuxiu Guo, Seray Cicek, Karen Colwill, Anne-Claude Gingras, Rod Bremner, Patricio Ponce, Keith Pardee, Varsovia Enid Cevallos

**Affiliations:** 1 Centro de Investigación en Enfermedades Infecciosas y Vectoriales (CIREV), Instituto Nacional de Investigación en Salud Pública (INSPI), Quito, Ecuador; 2 Facultad de Ciencias Médicas y Bienestar. Universidad Iberoamericana del Ecuador – UNIB.E, Quito, Ecuador; 3 Departamento de Ciencias de la Vida y la Agricultura, Universidad de las Fuerzas Armadas ESPE, Sangolquí, Ecuador; 4 Escuela de Enfermería, Facultad de Ciencias de la Salud, Universidad de las Américas, Quito, Ecuador; 5 Lunenfeld-Tanenbaum Research Institute at Mount Sinai Hospital, Sinai Health, Toronto, Ontario, Canada; 6 Department of Laboratory Medicine and Pathobiology, University of Toronto, Toronto, Ontario, Canada; 7 Department of Ophthalmology and Vision Science, University of Toronto, Toronto, Ontario, Canada; 8 Leslie Dan Faculty of Pharmacy, University of Toronto, Toronto, Ontario, Canada; 9 LSK Technologies Inc., Kitchener, Canada; 10 Department of Molecular Genetics, University of Toronto, Toronto, Ontario, Canada; 11 Department of Mechanical and Industrial Engineering, University of Toronto, Toronto, Ontario, Canada; Qatar University, QATAR

## Abstract

The COVID-19 pandemic underscored the critical need to enhance screening capabilities and streamline diagnosis. Point-of-care (POC) tests offer a promising solution by decentralizing testing. We aimed to validate the PLUM device (LSK Technologies Inc.), a portable optical reader, to detect SARS-CoV-2 RNA using direct RT-LAMP targeting the *ORF1a* and *E1* genes and patient antibodies by ELISA. The direct RT-LAMP assays employ nasopharyngeal swabs and bypass RNA extraction protocols through a brief chemical and physical lysis step. Test sensitivity and specificity were assessed against gold-standard detection methods in laboratory and field conditions. For samples with Ct values below 25, direct RT-LAMP showed 83% sensitivity and 90% specificity under laboratory conditions and 91% sensitivity and 92% specificity under field conditions. The nucleocapsid antigen antibody assay had 99% positive percent agreement (PPA) and 97% negative percent agreement (NPA), outperforming spike-RBD antigen (98% PPA, 92% NPA) and seroprevalence (98% PPA, 88% NPA) under laboratory conditions. Under field conditions, similar results were found for antibody detection for the nucleocapsid antigen (93% PPA; 100% NPA), spike-RBD (100% PPA; 94% NPA), and seroprevalence (90% PPA; 94% NPA). This study validated the PLUM device as a dual POC tool for direct RT-LAMP-based SARS-CoV-2 and ELISA-based COVID-19 antibody detection.

## Introduction

According to the World Health Organization (WHO), during the COVID-19 pandemic, there have been around 776,800,000 confirmed cases worldwide. From January 2020 to November 2024, Ecuador had over a million confirmed COVID-19 cases, representing 6% of the country’s population [1]. Furthermore, with the emergence of several variants, such as Omicron, a critical peak of cases was observed in the country from January to mid-February 2022, followed by another rise in July 2022 [2].

In Ecuador, most reported cases have come from patients in cities accessing the gold-standard viral detection method, RT-qPCR, which relies on centralized diagnostic laboratories with well-equipped and well-trained staff [3]. Even for those in urban areas, where testing was available for 30–45 USD, mid and low-income patients faced the challenges of accessing testing. Logistic and financial challenges were barriers to accessible diagnostics, confounding public health efforts and underscoring the need for distributed testing.

The COVID-19 pandemic has highlighted the need for accurate and accessible molecular diagnosis of infectious diseases. Efforts are underway to improve the performance of Point-of-care (POC) tests by incorporating advanced technologies such as microfluidics, biosensors, and artificial intelligence. These innovations may help improve the accuracy and speed of POC tests for SARS-CoV-2 detection while reducing costs [4]. However, developing POC tests presents several challenges, including maintaining sensitivity and specificity levels compared to gold-standard laboratory-based tests, reducing costs, and shorter processing times [5].

Further, after infection or vaccination, identifying the resulting antibodies is another crucial step to assess the immune response in patients. This can be used to monitor and correlate the outcomes of vaccinated patients across different vaccine technologies, non-infected but vaccinated individuals, and infected-recovered and vaccinated patients [6]. This information could be valuable for establishing more efficient booster application schedules and understanding the development of an immune response to SARS-CoV-2 [7].

A reliable POC test for virus identification and antibody response in remote locations could eliminate sample transport, which takes several hours and can compromise the sample’s integrity. It could also reduce sample processing times, enabling massive testing and faster clinical decisions [4,5]. The ideal scenario would use POC tests in diverse settings such as doctor offices, urgent care facilities, and temporary diagnosis services [3]. Therefore, a POC device that can detect SARS-CoV-2 and patient antibody response would be a valuable tool for healthcare providers in remote locations where access to centralized testing facilities may be limited.

Several POC tests and testing devices have been developed to detect SARS-CoV-2 using different technologies based on real-time PCR [8], reverse-transcription loop-mediated isothermal amplification (RT-LAMP), and lateral flow assays, such as antigen tests. Each test format provides varying levels of sensitivity and specificity and yields results within 5–50 minutes [9,10]. Likewise, POC tests have been designed to detect antibodies generated after COVID-19 infection or vaccination. Most POC serology tests have used a lateral flow design to detect IgM, IgG, or both within 10–25 minutes for slower tests [11,12] and 5–10 minutes for the fastest, depending on the physical properties of the sample. Other immunoassay-based techniques employ magnetic immunodetection [13] and microarray chips [14].

To decentralize testing, we are using an optical multimodal isothermal plate reader called PLUM (LSK Technologies Inc.), for conducting LAMP and enzyme-linked immunosorbent assays (ELISA) [15]. PLUM’s performance has been tested using cell-free synthetic biology tools to detect the Zika and Chikungunya viruses in serum samples [16]. Similarly, a PLUM device that can track changes in fluorescence (FluoroPLUM) was developed for direct RT-LAMP detection of SARS-CoV-2 in nasopharyngeal samples [17].

Our current study used the PLUM to detect color changes in the RT-LAMP and ELISA assays. Using the PLUM for the RT-LAMP assay allows semi-quantitative detection by changing the reaction from purple to blue, as the device will take measurements every minute [15]. The PLUM’s portability and ease of use make it an attractive device to respond to diagnostic needs in resource-limited settings where access to laboratory infrastructure is limited.

Here, we used the optical measurement capabilities of the PLUM to detect SARS-CoV-2 by direct RT-LAMP and antibodies by ELISA. Our study aimed to validate PLUM under laboratory conditions and assess its suitability as a POC device in a remote location in Ecuador (Tena, Napo province). We hypothesized that using the direct RT-LAMP protocol in the PLUM device would reduce virus detection time and provide similar specificity and sensitivity to RT-qPCR (i.e., >90% sensitivity and specificity for *E* gene, as previously shown [18,19]). We also expected the PLUM device to perform with similarly specificity and sensitivity to a conventional microplate reader in detecting antibodies (i.e., at 99% specificity a >90% and >80% sensitivity for RBD and Nucleocapsid, respectively, as shown previously [20]).

## Materials and methods

### Sample collection

Nasopharyngeal swabs from patients with suspected COVID-19 infection (n= 1200) and sera samples (n= 800) from other patients were collected from Hospital Carlos Andrade Marín (Quito, Pichincha province), Hospital Civil de Borbón (Borbón, Esmeraldas province), and Hospital José María Velasco Ibarra (Tena, Napo province). These samples were collected between January 2021 and January 2022. Samples were aliquoted and stored at -80°C until processing to reduce the freeze-thaw cycles.

Nasopharyngeal swabs were processed by RT-qPCR to confirm infection cases (n= 720 positive, n= 480 negative for SARS-CoV-2). From this group of samples, 149 positive and 151 negative samples were randomly chosen, maintaining the same distribution of Ct values, and tested for laboratory validation of the PLUM device by RT-LAMP. The sample size was determined based on the recommendation of the United States Food and Drug Administration (FDA) regarding the development of molecular diagnostic tests for SARS-CoV-2 [21]. Sera samples were processed by ELISA for antibody signals from the receptor-binding domain of the spike (spike-RBD) and nucleocapsid (N) antigens, and absorbance was measured in a microplate reader to confirm the presence of antibodies. All sera samples were tested by ELISA for laboratory validation of the PLUM device. For field validation, nasopharyngeal swabs (n= 30) and sera (n= 45) samples were collected from Hospital José María Velasco Ibarra (Tena, province of Napo) and processed *in situ*.

This study was approved under protocol N°042–2020 by an Ecuadorian Expedited Ethics Committee. The research was conducted according to the ethical principles of the World Medical Association Declaration of Helsinki. All the participants provided their written informed consent to participate in the study.

### RNA extraction and amplification of SARS-CoV-2

RNA extraction from nasopharyngeal samples was done using the ExtractMe viral RNA kit (Blirt, Cat. No. EM39) following the manufacturer’s instructions. The volume used for extraction was 100 μl of the sample, and the elution of RNA was performed with 30 μl of RNase-free water.

The *E* gene of the SARS-CoV-2 virus was amplified by RT-qPCR as described in previous protocols [18,22]. The SuperScript™ III Platinum™ One-Step RT-qPCR Kit (Invitrogen, Cat. No. 12574026) was used to detect *E* gene (E_Sarbeco_F ACAGGTACGTTAATAGTTAATAGCGT; E_Sarbeco_R ATATTGCAGCAGTACGCACACA) and the human *ACTB* gene (ACTB-F3 AGTACCCCATCGAGCACG; ACTB-B3 AGCCTGGATAGCAACGTACA) as an internal control. A Ct value of 35 was set as a cutoff for *E* and *ACTB* genes.

### Direct RT-LAMP in PLUM

Direct detection of SARS-CoV-2 was performed using nasopharyngeal swab samples and an RT-LAMP assay with the SARS-CoV-2 Nucleic Acid Amplification Kit (LSK, Cat. No. A2021F) targeting both the Open Reading Frame (*ORF1a*) and Envelope (*E1*) genes, as per the manufacturer’s instructions. Samples were lysed using 40 µl of lysis buffer and 10 µl of the swab sample, vortexed, and centrifuged at 1500 rpm for 1 minute. Then, the samples were incubated at 95°C for 5 minutes in the PLUM, cooled to 4°C, and kept on ice until the mixes for amplification were ready.

Mixes for SARS-CoV-2 and internal control (IC), included in the kit, were prepared with 4.6 µl of master mix, 3.4 µl of SARS-CoV-2 or IC primer mix, and 4 µl of nuclease-free water. Each reaction consisted of 12 µl of RT-LAMP mix (IC or viral) and 4 µl of sample or control. The final volume of each reaction was 16 µl. The samples were run in the PLUM (LSK Technologies Inc., Cat. No. SPF-005 and SPF-006) for 50 minutes at 65°C, using 96-well plates (Thermo Scientific™, Cat. No. AB0700). Using the PLUM for the direct RT-LAMP assay allows for semi-quantitative detection by the color change of the reaction from purple to blue, as the device will take measurements every minute [15].

PLUM RT-LAMP validation was done using 300 nasopharyngeal swab samples (n= 149 positive, n= 151 negative) under laboratory conditions and 60 samples under field conditions (n= 30 nasopharyngeal samples collected in the field, plus n= 30 samples previously tested by RT-qPCR under laboratory conditions). Positive controls included in the kit were used to enable cross-plate comparisons.

### ELISA assays: spike-RBD and N

The protocol for manual colorimetric ELISA described by Colwill and collaborators was modified as indicated [6]. High-binding 96-well plates (NEST, Cat. 514201) were coated with 50 µl of 0.9 µg/mL (0.45 µg/mL for the second batch after titration and comparison to the control batch to obtain similar performance) spike-RBD antigen (Sino Biological, Cat. No. 40592-VNAH) or 0.5 µg/mL N antigen (Sino Biological, Cat. No. 40588-V07E) diluted in sterile 1X PBS. Plates were incubated at 4°C overnight, washed three times with PBST (1X PBS + 0.1% TWEEN-20), blocked with 200 µl of Blok-Blotto blocking solution (G-Biosciences, Cat. No. 82022–630), and incubated for 75 minutes at room temperature on a shaker at 200 rpm. The blocking solution was discarded. Serum samples were incubated at 56°C for one hour in a dry bath/block for viral inactivation.

A standard curve of recombinant antibodies (anti-spike-RBD [SARS-CoV-2 Spike S1 Antibody (HC2001) Human Chimeric: GenScript, Cat. No. A02038-1] or anti-N [SARS-CoV-2 Nucleocapsid Antibody (HC2003) Human Chimeric: GenScript, Cat. No. A02039]) at known concentrations (8-point curve of 2-fold dilutions starting at 10 ng/well) was added to each plate to allow the calculation of antibody concentration in each sample. Samples, controls, and blanks were then added (50 µl per well, diluted in 1% w/v casein in PBST), and the plates were incubated for two hours at room temperature on a shaker at 200 rpm. After washing the plates three times with 200 µl of PBST, 50 µl of anti-human IgG HRP-conjugated secondary antibody (Jackson ImmunoResearch, Cat. No. 109-035-098), diluted 1:60000 in 1% w/v casein in PBST was added to each well. Plates were incubated for 1 hour at room temperature on a shaker at 200 rpm and washed three times with 200 µl of PBST. Then, in each well was added 50 µl of 1-Step™ Ultra TMB-ELISA Substrate Solution (Thermo Scientific™, Cat. No. PI34029). After 15 minutes of incubation in the dark, the reaction was stopped with a Stop solution (Thermo Scientific™ Cat. No. PIN600) for the spike-RBD antigen or 0.2N H_2_SO_4_ for the N antigen. The absorbance was measured in a GEA microplate reader (Linear, Cat. 6802000) at 450 nm and in the PLUM device under the set parameters for ELISA by the manufacturer. Each sample was run in duplicate. Four samples from non-infected and non-vaccinated patients and one human IgG (Sigma, Cat. No. I4506) diluted 1:1000 were used as negative controls for cross-plate comparisons. A pool of eight positive sera was used as a positive control. One percent casein in PBST was used as a blank, and its absorbance was subtracted from sera values for further calculations (relative value).

A total of 800 sera samples were used for laboratory validation and 62 for field validation (n= 45 sera samples collected in the field, plus n= 17 samples that tested negative under laboratory conditions). Additionally, 60 pre-pandemic sera samples from patients with antibodies against dengue were assayed to test the specificity of our ELISA assays. These pre-pandemic samples were de-identified and donated by the National Surveillance and Reference Laboratory for Exanthematic and Gastroenteric Viruses (INSPI, Guayaquil – Ecuador) as per institutional regulations.

### Data analysis

#### Receiver-operating characteristic (ROC) analysis.

The Receiver-operating characteristic (ROC) analysis for detecting SARS-CoV-2 by RT-LAMP was done with 106 positive and 144 negative nasopharyngeal swab samples confirmed by RT-qPCR.

The ROC analysis for detecting antibodies by ELISA was performed using relative ratios with individual replicates considered individual samples. Samples with different results between replicates were removed from the analysis to avoid skewed threshold determination. These sera samples were collected before the availability of the COVID-19 vaccine in Ecuador. Positive samples (n= 94) were from patients diagnosed with SARS-CoV-2 infection. Negative samples (n= 84) were from asymptomatic PCR-negative patients without contact with a known case. Additionally, 230 negative controls (blanks, IgG pools, 0.16 and 0.0781 ηl/well of the standard curve of recombinant antibodies) were included in the analysis.

#### Statistical analysis.

Data are expressed as mean ± SEM. Data were tested for normality of the distribution by the D’Agostino–Pearson test with a confidence interval of 95%. Statistical comparisons were performed using the Mann–Whitney U test and the Spearman method. ROC analyses were generated using the Wilson/Brown method with a confidence interval of 95%. The thresholds were selected to maximize sensitivity and specificity based on ROC curve analysis. A Cohen’s kappa coefficient was used to assess interobserver variability; *k* ≥ 0.8 was considered to show a near-perfect agreement. All statistics were calculated, and graphs were plotted using GraphPad Prism Software version 9.01 for macOS (GraphPad Software, La Jolla, California, USA).

The sensitivity, specificity, positive predictive value, negative predictive value, and accuracy of the RT-LAMP and ELISA were calculated using the web-based software MedCalc’s Diagnostic Test Evaluation Calculator (https://www.medcalc.org/calc/diagnostic_test.php). Agreement rates and associated Clopper-Pearson 95% CIs of the ELISA wider sample size were calculated using the Westgard QC online 2 × 2 contingency calculator tool [23].

## Results

### Detection of SARS-CoV-2 by RNA extraction and RT-qPCR

**Fig 1:**
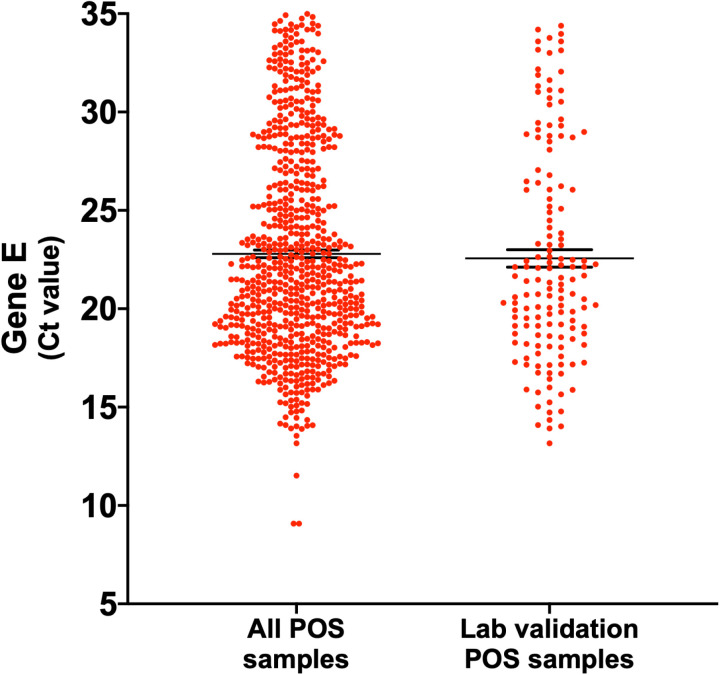
Distribution of nasopharyngeal samples positive for SARS-CoV-2. Comparison of the Ct values between all positive samples (n= 720) and the positive samples selected for laboratory validation of RT-LAMP (n= 149). Mean ± SEM. Mann-Whitney *U* test (*p* > 0.05).

A total of 1200 nasopharyngeal samples were processed by RNA extraction and detection of SARS-CoV-2 by RT-qPCR amplification of the *E* gene. Our results showed that 60% of the samples (n=720) were positive for the virus with Ct values ranging from 9.08 to 34.99 (mean 22.79; SEM ± 0.20) (Fig 1).

For the laboratory validation of RT-LAMP, we randomly selected 25% of the total nasopharyngeal samples collected (n= 149 positive; n= 151 negative), maintaining the same distribution of Ct values (13.16 to 34.39; mean 22.56; SEM ± 0.44; *p* > 0.05) (Fig 1) and negative samples.

### Detection of SARS-CoV-2 by direct RT-LAMP assay in the PLUM - Laboratory validation

**Fig 2:**
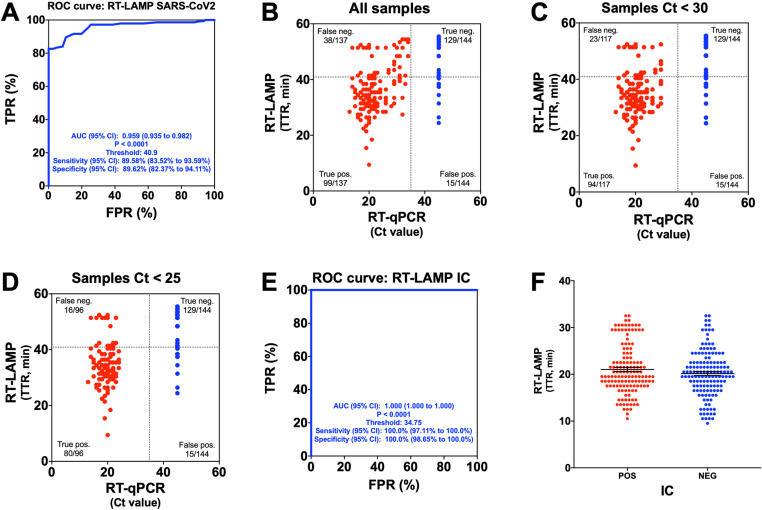
Direct RT-LAMP in the PLUM, validation under laboratory conditions. (A) ROC curve evaluating the PLUM’s performance for detecting SARS-CoV-2 by direct RT-LAMP. (B) Distribution of RT-LAMP TTRs against RT-qPCR Ct values for 137 positive (red dots) and 144 negative (blue dots) nasopharyngeal samples. RT-qPCR and RT-LAMP positives were defined by Ct < 35.0 and TTR ≤ 40.9’, respectively (dotted lines). (C) Distribution of RT-LAMP and RT-qPCR values for 117 positive and 144 negative nasopharyngeal samples with Ct values below 30. (D) Distribution of RT-LAMP and RT-qPCR values for 96 positive and 144 negative nasopharyngeal samples with Ct values below 25. (E) ROC curve evaluating the PLUM’s performance for detecting internal control (IC) by direct RT-LAMP. (F) Distribution of RT-LAMP TTRs for IC from 137 positive and 144 negative samples as determined by RT-qPCR for SARS-CoV-2. Mean ± SEM. Mann-Whitney *U* test (*p* > 0.05).

The **receiver-operating characteristic (**ROC) analysis of the RT-LAMP assays targeting the *ORF1a* and *E* genes showed a time-to-result (TTR) threshold of 40.9 minutes. This cutoff revealed a sensitivity of 89.58% (95% CI 83.52% to 93.59%) and a specificity of 89.62% (95% CI 82.37% to 94.11%) of the assay (AUC 0.959; 95% CI 0.935 to 0.982; *p* < 0.0001) (Fig 2A).

Of the 300 nasopharyngeal samples used for laboratory validation of the direct RT-LAMP assay in the PLUM device, 19 samples did not amplify for the internal control (IC) and were eliminated from further analyses. Hence, we analyzed the results from 137 positive and 144 negative samples, as determined by RT-qPCR results (Table 1). Our results showed that the assay’s sensitivity depends on the sample’s viral load. RT-LAMP performed using the PLUM device showed 89.6% specificity and 72.3% sensitivity for all the positive samples detected by RT-qPCR (Ct values ranging from 13.16 to 34.19) (Table 1; Fig 2B). However, sensitivity increased to 80.3% and 83.3% for samples with Ct values below 30 (Ct values ranging from 13.16 to 29.45) and 25 (Ct values ranging from 13.16 to 24.49), respectively (Table 1; Figs 2B and 2C). Spearman’s rank correlation was computed to assess the relationship between RT-qPCR and direct RT-LAMP results. There was a positive correlation between the two variables for all samples (r(279)= 0.69, *p*= <0.0001), samples with Ct below 30 (r(259)= 0.71, *p*= <0.0001), and samples with Ct below 25 (r(238)= 0.70, *p*= <0.0001).

**Table 1:** Diagnostic performance of SARS-CoV-2 detection by direct RT-LAMP assay in the PLUM using nasopharyngeal samples, laboratory validation.

	All samples			Ct < 30			Ct < 25		
RT-qPCR +	RT-qPCR -	Total	RT-qPCR +	RT-qPCR -	Total	RT-qPCR +	RT-qPCR -	Total
RT-LAMP +	99	15	114	94	15	109	80	15	95
RT-LAMP -	38	129	167	23	129	152	16	129	145
Total	137	144	281	117	144	261	96	144	240
Sensitivity (95% CI)	72.26% (63.97% to 79.57%)			80.34% (71.98% to 87.11%)			83.33% (74.35% to 90.16%)		
Specificity (95% CI)	89.58% (83.40% to 94.05%)			89.58% (83.40% to 94.05%)			89.58% (83.40% to 94.05%)		
Prevalence (95% CI)	48.75% (42.77% to 54.76%)			44.83% (38.69% to 51.08%)			40.00% (33.75% to 46.50%)		
PPV (95% CI)	86.84% (80.17% to 91.51%)			86.24% (79.38% to 91.07%)			84.21% (76.62% to 89.67%)		
NPV (95% CI)	77.25% (72.04% to 81.73%)			84.87% (79.47% to 89.04%)			88.97% (83.71% to 92.68%)		
Accuracy (95% CI)	81.14% (76.07% to 85.54%)			85.44% (80.57% to 89.49%)			87.08% (82.17% to 91.05%)		

The results from the IC amplification were analyzed to determine potential differences between positive (n=137) and negative samples (n=144) for SARS-CoV-2, according to RT-qPCR results. A TTR threshold of 34.75 for the IC amplification revealed a sensitivity of 100% (95% CI 97.11% to 100%) and a specificity of 100% (95% CI 98.65% to 100%) of the assay (Fig 2E). Furthermore, there were no statistical differences when comparing the IC amplification TTR values between positive (10.50 to 32.50; mean: 21.02; SEM: ± 0.45) and negative samples (9.50 to 32.50; mean 20.14; SEM ± 0.42; *p* > 0.05) (Fig 2F).

### Detection of SARS-CoV-2 by direct RT-LAMP assay in the PLUM - Field validation

**Fig 3:**
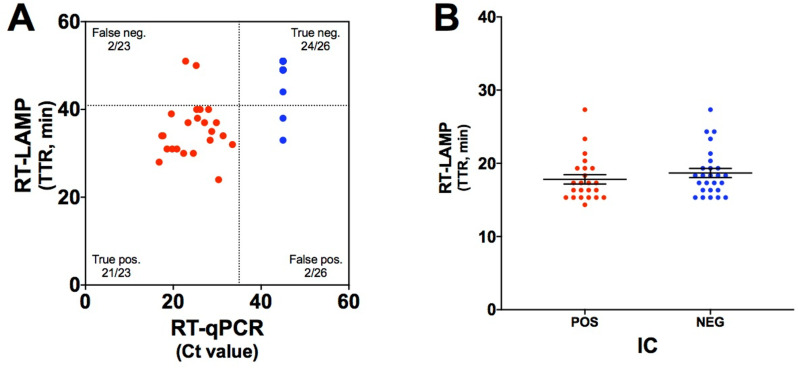
Direct RT-LAMP in the PLUM, validation under field conditions. (A) Distribution of RT-LAMP TTRs against RT-qPCR Ct values for 23 positive (red dots) and 26 negative (blue dots) nasopharyngeal samples. RT-qPCR and RT-LAMP positives were defined by Ct < 35.0 and TTR ≤ 40.9’, respectively (dotted lines). (B) Distribution of RT-LAMP TTRs for IC from 23 positive and 26 negative samples as determined by RT-qPCR for SARS-CoV-2. Mean ± SEM. Mann-Whitney *U* test (*p* > 0.05).

We tested 60 nasopharyngeal samples to validate the RT-LAMP assay using the PLUM under field conditions. However, 11 samples did not amplify for the IC and were excluded from further analysis. Hence, the analyses were based on 23 positive samples (Ct values ranging from 16.20 to 33.47) and 26 negative samples as determined by RT-qPCR (Fig 3A).

SARS-CoV-2 prevalence in the field-collected patient samples was 46.9% (95% CI 32.5% to 61.7%). The direct RT-LAMP assay in the PLUM under field conditions showed a sensitivity of 91.3% (95% CI 72.0% to 98.9%) and a specificity of 92.3% (95% CI 74.9% to 99.0%). The positive predictive value (PPV) was 91.3% (95% CI 73.4% to 97.6%). The negative predictive value (NPV) was 92.3% (95% CI 76.1% to 97.8%). The overall accuracy of the RT-LAMP assay under field conditions was 91.8% (95% CI 80.4% to 97.7%). There was a positive correlation between the RT-qPCR and direct RT-LAMP results tested under field validation (r(47)= 0.69, *p*= <0.0001).

Using the TTR threshold of 34.75, our results showed no statistical differences in IC amplification between positive (14.33 to 27.33; mean 17.81; SEM ± 0.64) and negative samples (15.33 to 27.33; mean 18.68; SEM ± 0.62; *p* > 0.05) (Fig 3B).

### Receiver-operating characteristic (ROC) analysis - ELISA assays

A ROC analysis combined data from six months of ELISA assays performed on a conventional microplate reader (defined as gold-standard ELISA for this comparison). A total of 408 samples were used for this analysis, consisting of 94 COVID-19-diagnosed convalescent individuals (10–57 days post-symptom onset) and 84 non-vaccinated, PCR-negative samples, plus 230 negative controls, which were used for this analysis.

A false positive rate of < 1% revealed sensitivities of 99.8% and 99.7% for the spike-RBD and N antigens, respectively (Table 2). A sample was considered positive only when both antigens exceeded the threshold, minimizing the risk of false results. Our results showed a combined specificity of 100.0% and a sensitivity of 98.9% (Table 2). The same ROC analysis was performed for the ELISA assays in the PLUM device, showing sensitivities of 99.8% and 99.7% for spike-RBD and N antigens, respectively, and a combined specificity of 100% and sensitivity of 99.4% (Table 2).

**Table 2:** ELISA performance statistics for serum IgG detection of spike-RBD and N antigens and seroprevalence.

	Spike-RBD	N	Seroprevalence
**Microplate reader**			
AUC (95% CI)	1.000 (1.000 to 1.000)*	1.000 (1.000 to 1.000)*	N/A
Threshold	1.396	0.807	N/A
Sensitivity (95% CI)	99.80% (98.86% to 99.99%)	99.71% (98.37% to 99.99%)	98.86% (95.96% to 99.86%)
Specificity (95% CI)	100.0% (97.95% to 100.0%)	100.0% (97.86% to 100.0%)	100.00% (98.91% to 100.00%)
**PLUM**			
AUC (95% CI)	1.000 (1.000 to 1.000)*	1.000 (1.000 to 1.000)*	N/A
Threshold	1.674	0.811	N/A
Sensitivity (95% CI)	99.79% (98.80% to 99.99%)	99.68% (98.23% to 99.98%)	99.44% (96.93% to 99.99%)
Specificity (95% CI)	100.0% (98.00% to 100.0%)	100.0% (97.89% to 100.0%)	100.00% (98.78% to 100.00%)

Results from microplate reader (absorbance) and PLUM (PLUM reading units - PRU). ROC by Wilson/Brown method (*p < 0.0001).

### Detection of COVID-19 antibodies by ELISA assay in PLUM - Laboratory validation

**Fig 4:**
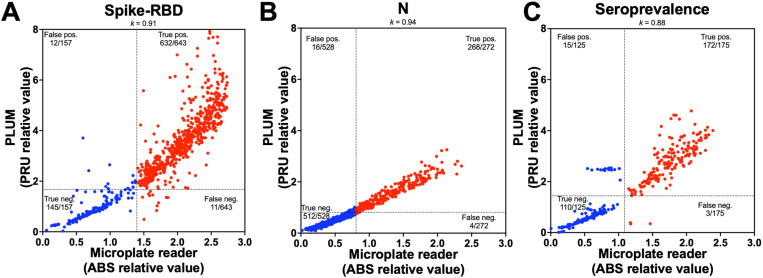
Detection of COVID-19 antibodies in sera samples by ELISA assays in the PLUM device, validation under laboratory conditions. (A) Distribution of spike-RBD ELISA absorbance (ABS) relative ratio as determined by a microplate reader against PLUM reading units (PRU) relative ratio from 643 positive (red dots) and 153 negative (blue dots) sera samples. (B) Distribution of **N** ELISA ABS relative ratio against PRU relative ratio from 272 positive and 528 negative sera samples. (C) Distribution of seroprevalence results from the average ABS from spike-RBD and N against the average PRU from the same antigens using 175 positive and 125 negative sera samples. Cohen’s kappa statistics are shown. Dashed lines represent the threshold determined by ROC curve analysis (Table 3).

We used a PLUM reading unit (PRU) threshold of 1.674 for spike-RBD antigens (Table 2). Our results, using 643 positive and 157 negative samples as determined by the gold-standard ELISA assay, showed a 98.3% positive percent agreement (PPA) and 92.4% negative percent agreement (NPA) for detecting antibodies against spike-RBD (Table 3, Fig 4A). Likewise, a PRU threshold of 0.811 for N, with 272 positive and 528 negative samples, showed a 98.5% PPA and 97.0% NPA (Table 3, Fig 4B). There was a very good agreement between the microplate reader and the PLUM results for both antigens (*k* > 0.90) (Table 3).

**Table 3:** Diagnostic performance of COVID-19 antibodies detection by ELISA assays in PLUM using sera samples, laboratory validation.

	Spike-RBD			N			Seroprevalence		
Microp. +	Microp. -	Total	Microp. +	Microp. -	Total	Microp. +	Microp. -	Total
PLUM +	632	12	644	268	16	284	172	15	187
PLUM -	11	145	156	4	512	516	3	110	113
Total	643	157	800	272	528	800	175	125	300
PPA (95% CI)	98.29% (96.96% to 99.14%)			98.53% (96.28% to 99.60%)			98.29% (95.07% to 99.65%)		
NPA (95% CI)	92.36% (87.03% to 95.99%)			96.97% (95.13% to 98.26%)			88.00% (80.98% to 93.13%)		
OPA (95% CI)	97.12% (95.72% to 98.17%)			97.50% (96.17% to 98.47%)			94.00% (90.68% to 96.41%)		
Kappa (95% CI)	0.91 (0.87 to 0.95)			0.94 (0.92 to 0.97)			0.88 (0.82 to 0.93)		

Results from positive percent agreement (PPA), negative percent agreement (NPA), overall percent agreement (OPA), and Cohen’s kappa statistic (kappa).

We used 300 sera samples (n= 175 positive; n= 125 negative) that showed concordant results for both antigens from the gold-standard assay for seroprevalence. Our results showed a 98.3% PPA and 88.0% NPA (Table 3, Fig 4C). There was a very good agreement between the microplate reader and the PLUM results for seroprevalence (*k*= 0.88) (Table 3).

The specificity of our ELISA assays was further analyzed using pre-pandemic negative samples that were also positive for antibodies (IgM) against dengue. No cross-reaction was detected when samples were tested against spike-RBD or N antigens.

### Detection of COVID-19 antibodies by ELISA assay in the PLUM - Field validation

**Fig 5:**
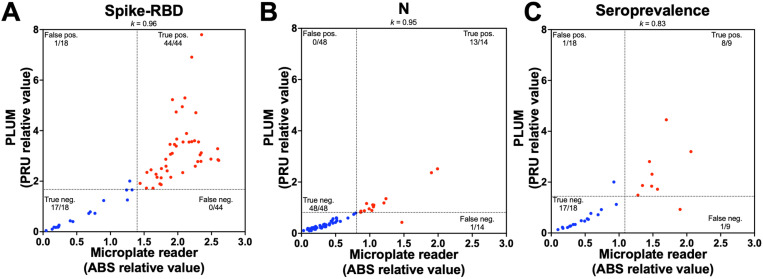
Detection of COVID-19 antibodies in sera samples by ELISA assays in the PLUM device, validation under field conditions. (A) Distribution of spike-RBD ELISA absorbance (ABS) relative ratio as determined by a microplate reader against PLUM reading units (PRU) relative ratio from 44 positive (red dots) and 18 negative (blue dots) sera samples. (B) Distribution of **N** ELISA ABS relative ratio against PRU relative ratio from 14 positive and 48 negative sera samples. (C) Distribution of seroprevalence results from the average ABS from spike-RBD and N against the average PRU from the same antigens using nine positive and 18 negative sera samples. Cohen’s kappa statistics are shown. Dashed lines represent the threshold determined by ROC curve analysis (Table 3).

Our results showed a 100% PPA and 94.4% NPA for detecting antibodies against spike-RBD by the PLUM under field conditions (Table 4, Fig 5A), with a PRU threshold of 1.674 and testing 44 positive and 18 negative samples as determined by the microplate reader. Likewise, our results showed a 92.9% PPA and 100% NPA against N by the PLUM (Table 4, Fig 5B), with a PRU threshold of 0.811 and testing 14 positive and 48 negative samples. There was agreement between the microplate reader and the PLUM results for spike-RBD (*k* ≥ 0.95) (Table 4).

**Table 4:** Diagnostic performance of COVID-19 antibodies detection by ELISA assays in the PLUM using sera samples, field validation.

	Spike-RBD			N			Seroprevalence		
Microp. +	Microp. -	Total	Microp. +	Microp. -	Total	Microp. +	Microp. -	Total
PLUM +	44	1	45	13	0	13	8	1	9
PLUM -	0	17	17	1	48	49	1	17	18
Total	44	18	62	14	48	62	9	18	27
PPA (95% CI)	100.00% (91.96% to 100.00%)			92.86% (66.13% to 99.82%)			88.89% (51.75% to 99.72%)		
NPA (95% CI)	94.44% (72.71% to 99.86%)			100.00% (92.60% to 100.00%)			94.44% (72.71% to 99.86%)		
OPA (95% CI)	98.39% (91.34% to 99.96%)			98.39% (91.34% to 99.96%)			92.59% (75.71% to 99.09%)		
Kappa (95% CI)	0.96 (0.88 to 1.00)			0.95 (0.86 to 1.00)			0.83 (0.61 to 1.00)		

Results from positive percent agreement (PPA), negative percent agreement (NPA), overall percent agreement (OPA), and Cohen’s kappa statistic (kappa).

For seroprevalence, we determined an 88.9% PPA and 94.4% NPA by the PLUM (Table 3, Fig 5C) under field conditions (n= 8 positive; n= 18 negative). There was agreement between the microplate reader and PLUM results for seroprevalence (*k* = 0.87) (Table 4).

## Discussion

This study demonstrated the PLUM device’s potential as a versatile point-of-care (POC) diagnostic platform for detecting SARS-CoV-2 by direct RT-LAMP and COVID-19 antibodies by ELISA. The PLUM offers significant advantages, reducing the time-to-result (TTR), eliminating the need for complex laboratory infrastructure, and minimizing costs associated with sample transport and processing. Regarding the sensitivity and specificity of the PLUM, virus detection depends on the sample’s viral load, while antibody detection performs similarly to an ELISA assay, where absorbance is measured in a microplate reader. These attributes align with the World Health Organization’s ASSURED criteria (Affordable, Sensitive, Specific, User-friendly, Rapid and robust, Equipment-free, and Deliverable), making the PLUM device a practical option for decentralized healthcare applications.

### Enhanced accessibility and decentralized viral detection

Multiple POC tests have been designed to detect SARS-CoV-2 by RT-LAMP either with RNA extraction [24–31] or without conventional RNA extraction [17,32–37]. Our direct RT-LAMP assay bypasses RNA extraction protocols by a five-minute chemical (lysis buffer) and physical (thermal shock) lysis step, reducing time, reagents, and consumables. Likewise, it meets the criteria for minimal sample manipulation –an essential feature of effective POC tests [38]. Furthermore, using the PLUM for the direct RT-LAMP assay allows for semi-quantitative viral detection through a visible color change from purple to blue, supported by kinetic measurements taken every minute [15].

Under laboratory conditions, our direct RT-LAMP assay showed 83% sensitivity and 90% specificity, with performance dependent on the sample’s viral load. Importantly, it effectively identified patients with high viral loads (Ct < 25), who are more likely to transmit the virus. While further optimization is needed to enhance detection for lower viral loads (Ct > 30), the current configuration offers substantial public health benefits, particularly in low-resource settings where identifying highly infectious individuals is critical.

In this regard, for the *Sarbeco E* gene, measured with the SuperScript^TM^ III Platinum^TM^ One-Step RT-PCR System, a Ct value of 33 corresponds to viral loads of 440 *in vitro* transcribed RNA copies/µl [39]. The viral load of COVID-19 patients is a leading driver for SARS-CoV-2 transmission. Patients with a viral load of 1x10^7^ copies per µl or higher showed a secondary attack rate of 24% [40]. Hence, patients with Ct values lower than 30 for the *Sarbeco E* gene would have a high viral load detectable by our direct RT-LAMP assay. Furthermore, the internal control did not show differences in amplification between positive and negative samples. These results showed that the samples had a quantifiable amount of good-quality RNA and did not contain inhibitory agents for the detection reaction.

Hence, the PLUM device complies with the ASSURED criteria outlined by the WHO, as it is rapid, robust, and maintains sensitivity and specificity in viral detection. Additionally, the PLUM device’s affordability is notable; the assay costs less than USD 15 and produces results within two hours, compared to USD 30–45 for RT-qPCR tests in Ecuador, which typically take 24 hours to yield results. Likewise, the device’s intuitive design requires minimal training, making it accessible to healthcare workers without specialized technical expertise. The lightweight and portable nature ensures ease of distribution and use across geographically isolated regions. Finally, the self-contained nature of the device obviates the need for external power, with minimal equipment (vortex and centrifuge) needed, ensuring functionality in off-grid settings.

### Comparison of molecular testing modalities

Several POC tests for SARS-CoV-2 detection have been validated, utilizing equipment of varying complexity. For instance, a multiplex RT-LAMP assay that can be incubated in a heat block showed 73% sensitivity and 89% specificity, with results available within an hour based on color change [32]. In contrast, the direct PLUM-based RT-LAMP assay reported here showed higher sensitivity and specificity. The PLUM device offers an additional advantage of semi-quantitative detection through continuous kinetic measurements. Other POC tests using specific equipment to detect SARS-CoV-2 have shown higher sensitivity and specificity [17,34,35]. However, these tests often need to meet the ASSURED criteria for affordability and equipment-free operation, which is particularly challenging for health centers in remote locations. With its low cost and remote data interpretation capabilities facilitated by Wi-Fi connectivity, the PLUM device is well-suited for decentralized settings [15]. These features highlight the potential of the PLUM device as an effective and accessible tool for decentralized SARS-CoV-2 testing in resource-limited settings.

The reported RT-LAMP results, under field conditions, showed 91% sensitivity and 92% specificity. According to the WHO, diagnostic tests with ≥ 80% sensitivity and ≥ 97% specificity are suitable replacements for laboratory-based RT-PCR when the latter cannot be delivered promptly [22]. Hence, our results highlight the potential of the PLUM device and direct RT-LAMP assay to provide POC testing for SARS-CoV-2 detection from nasopharyngeal samples in remote locations. Even so, having limited equipment in the laboratory presented some challenges during the setup of the assays. For instance, a lack of centrifuges for microtubes and microplates meant that the reagents and samples might not settle at the bottom of the tubes. In this regard, we employed alternative methods to improve the precipitation of the solutions. We tapped the microplates to remove as many bubbles as possible and ensure that most of the mix covered the well before starting the incubation into the PLUM. These challenges should be considered for future assays under reduced equipment scenarios.

### Comparison of antibody detection

Several point-of-care (POC) tests have been developed to detect antibodies following COVID-19 infection or vaccination, primarily using lateral flow designs for rapid IgM and IgG detection. Commercially available rapid test cassettes have shown varying sensitivity and specificity for IgG detection [41–44]. However, these tests often lack information about their target antigens, making it challenging to assess their suitability for future SARS-CoV-2 variants. Our proposed ELISA POC test, which targets antigens from the original Wuhan strain, may face reduced sensitivity to later variants, such as Omicron, but offers adaptability for variant-specific testing and other disease agents.

Our in-house ELISA assay for IgG Spike-RBD detection using the PLUM device demonstrated higher sensitivity (99.8%) and specificity (100%), as determined by ROC analysis results, when compared to other studies [41,43,44]. However, it is crucial to acknowledge that our ROC results were derived from a sample size of fewer than 100, whereas the other studies employed larger sample sizes. Nevertheless, when expanded to a larger scale (n=800), our in-house ELISA assay for IgG Spike-RBD detection using the PLUM device showed 98% PPA and 92% NPA under laboratory conditions relative to the microplate reader. Under field conditions, it showed 100% PPA and 94% NPA. Our assay showed higher sensitivity (PPA) under laboratory and field validation but lower specificity (NPA) than the abovementioned results. Previous research has shown that the sensitivity and specificity of antibody detection with POC tests are influenced by the number of days after infection when samples are collected. Studies have indicated that samples collected more than 14 days after testing positive for SARS-CoV-2 exhibit 100% sensitivity and specificity [43,44], while samples collected more than 140 days post-infection show a reduced sensitivity [45]. While our study had limitations in confirming infection dates for participants (90 out of 800), our results suggest strong performance of our ELISA assay for IgG Spike-RBD detection on the PLUM device, particularly in field conditions.

Our in-house ELISA assay for IgG Nucleocapsid using the PLUM device showed 99% PPA and 97% NPA under laboratory conditions compared to the microplate reader. Under field conditions, it showed 93% PPA and 100% NPA. Our results are comparable to an in-house ELISA based on the SARS-CoV-2 N, which showed 94% sensitivity and 100% specificity for the combined detection of IgA, IgM, and IgG [46]. Within our cohort, vaccinated patients received varying numbers of doses from AstraZeneca, CanSino, Pfizer-BioNTech, and/or CoronaVac (data not shown). Since CoronaVac is the only vaccine containing inactivated viruses that would react to both spike-RBD and N antigens used in this study, these samples were excluded from our seroprevalence analyses (Spike-RBD and N). Our results using the PLUM device showed 98% PPA and 88% NPA under laboratory conditions and 90% PPA and 94% NPA under field conditions. These results indicate that our proposed ELISA POC test has the potential to provide accurate antibody detection in primary care and POC settings. For comparison, in Ecuador, a rapid qualitative test for IgM/IgG detection costs around USD 17, while a quantitative test costs USD 40, with results available within a 24 hours. The ELISA POC tests reported here (targeting Spike-RBD and N antigens) could be available for less than USD 10 with results within five hours.

## Conclusions

Our study validated the PLUM as a dual-function POC diagnostic platform for detecting SARS-CoV-2 and conducting COVID-19 antibody testing, meeting WHO’s ASSURED criteria. Its affordability, portability, and minimal training requirements make it particularly well-suited for deployment in remote and resource-limited settings. Additionally, the assays reduce the time required to produce results and lower sample processing costs. Regarding sensitivity and specificity rates, the sensitivity of the SARS-CoV-2 detection depends on the sample’s viral load, while antibody detection in this study matched the performance of a gold-standard technique. Furthermore, due to the flexibility of RT-LAMP and ELISA assays, we envision that these methods and infrastructure reported here could be adapted to detect specific SARS-CoV-2 variants, as well as other disease agents. Its deployment could significantly enhance disease surveillance and control efforts, particularly in regions where traditional diagnostic modalities are impractical or unavailable.

## Supporting information

S1 File RT-qPCR. (PDF)

S2 File RT-LAMP laboratory validation. (PDF)

S3 File RT-LAMP field validation. (PDF)

S4 File ELISA laboratory validation for Spike-RBD. (PDF)

S5 File ELISA laboratory validation for Nucleocapsid. (PDF)

S6 File ELISA laboratory validation for Seroprevalence. (PDF)

S7 File ELISA field validation for Spike-RBD. (PDF)

S8 File ELISA field validation for Nucleocapsid. (PDF)

S9 File ELISA field validation for Seroprevalence. (PDF)

S10. Fig Graphical abstract. (TIF)
