# Supplementary material for: Validation of new equipment for SARS-CoV-2 diagnosis in Ecuador: Detection of the virus and antibodies generated by disease and vaccines with one POC device
Source: PLoS One. 2025 Apr 16;20(4):e0321794. doi: 10.1371/journal.pone.0321794 (PMC12002511; doi:10.1371/journal.pone.0321794)
Supplement: S1 File — (PDF) [file pone.0321794.s001.pdf]

SAMPLE	RT-qPCR_E	Ct_E	RT-LAMP
1001	Negative		
1003	Negative		Lab_validation
1004	Negative		
1005	Negative		Lab_validation
1006	Negative		
1007	Negative		
1008	Negative		
1009	Negative		Lab_validation
1010	Negative		
1012	Negative		
1013	Negative		Lab_validation
1014	Negative		
1015	Negative		
1016	Negative		
1017	Negative		Lab_validation
1018	Negative		
1019	Negative		
1020	Negative		Lab_validation
1021	Negative		
1022	Negative		
1023	Negative		Lab_validation
1024	Negative		
1025	Negative		Lab_validation
1026	Negative		
1027	Negative		Lab_validation
1029	Negative		
1030	Negative		Lab_validation
1031	Negative		
1032	Negative		Lab_validation
1033	Negative		
1034	Negative		
1035	Negative		Lab_validation
1036	Negative		Lab_validation
1037	Negative		
1038	Negative		
1039	Negative		Lab_validation
1040	Negative		
1041	Negative		
1042	Negative		Lab_validation
1043	Negative		
1044	Negative		Lab_validation
1045	Negative		
1046	Negative		Lab_validation
1047	Negative		
1048	Negative		Lab_validation
1049	Negative		
1050	Negative		
1051	Negative		Lab_validation
1052	Negative		

1054	Negative		
1055	Negative		
1057	Negative		
1058	Negative		
1059	Negative		Lab_validation
1060	Negative		
1061	Negative		Lab_validation
1062	Negative		
1063	Negative		
1064	Negative		
1065	Negative		
1066	Negative		
1067	Negative		Lab_validation
1068	Negative		
1070	Negative		
1071	Negative		
1072	Negative		Lab_validation
1073	Negative		
1074	Negative		
1075	Negative		
1076	Negative		Lab_validation
1077	Negative		
1079	Negative		
1080	Negative		Lab_validation
1081	Negative		
1082	Negative		
1083	Negative		Lab_validation
1084	Negative		Lab_validation
1085	Negative		Lab_validation
1086	Negative		Lab_validation
1087	Negative		
1088	Negative		
1089	Negative		
1090	Negative		
1091	Negative		
1092	Negative		Lab_validation
1094	Negative		
1095	Positive	20.24	
1096	Negative		
1097	Negative		Lab_validation
1098	Negative		
1099	Negative		
1100	Negative		Lab_validation
1101	Negative		
1102	Negative		Lab_validation
1103	Negative		Lab_validation
1104	Negative		
1105	Negative		Lab_validation
1107	Negative		Lab_validation
1108	Negative		Lab_validation

1109	Negative		
1110	Negative		
1111	Negative		Lab_validation
1112	Negative		Lab_validation
1113	Negative		
1114	Positive	17.29	Lab_validation
1115	Negative		
1116	Negative		
1117	Negative		
1119	Negative		Lab_validation
1120	Positive	16.82	
1121	Positive	16.75	Lab_validation
1122	Negative		Lab_validation
1123	Negative		Lab_validation
1124	Positive	28.39	
1125	Negative		
1126	Positive	24.47	
1127	Negative		
1128	Negative		
1129	Negative		
1130	Negative		Lab_validation
1131	Negative		Lab_validation
1132	Negative		
1133	Negative		
1134	Negative		Lab_validation
1135	Negative		
1136	Negative		Lab_validation
1138	Negative		Lab_validation
1139	Negative		
1140	Negative		Lab_validation
1141	Negative		
1142	Negative		Lab_validation
1143	Negative		
1144	Negative		Lab_validation
1145	Negative		
1146	Negative		
1147	Positive	16.70	Lab_validation
1148	Negative		
1149	Negative		Lab_validation
1150	Negative		Lab_validation
1151	Negative		
1152	Negative		Lab_validation
1153	Positive	17.34	
1154	Negative		Lab_validation
1155	Negative		
1156	Positive	20.22	
1157	Positive	28.86	
1158	Positive	22.22	Lab_validation
1159	Negative		Lab_validation
1160	Negative		

1161	Negative		Lab_validation
1162	Negative		
1164	Negative		
1165	Negative		
1166	Negative		Lab_validation
1167	Negative		
1168	Positive	16.56	
1169	Negative		Lab_validation
1170	Negative		
1171	Negative		
1172	Negative		Lab_validation
1173	Negative		
1174	Negative		Lab_validation
1175	Negative		Lab_validation
1176	Negative		
1177	Negative		
1178	Negative		Lab_validation
1180	Negative		
1181	Negative		
1182	Negative		Lab_validation
1183	Negative		
1184	Negative		
1185	Negative		
1186	Negative		Lab_validation
1187	Negative		
1188	Negative		
1189	Positive	25.70	
1190	Positive	16.33	
1191	Negative		
1192	Negative		Lab_validation
1194	Negative		
1195	Negative		
1196	Negative		Lab_validation
1197	Negative		
1198	Negative		
1199	Negative		
1200	Negative		
1201	Negative		Lab_validation
1202	Negative		
1203	Negative		
1204	Negative		
1205	Negative		
1206	Negative		
1207	Negative		
1208	Negative		Lab_validation
1209	Negative		
1210	Positive	19.22	Lab_validation
1211	Negative		
1212	Negative		
1213	Negative		

1214	Negative		Lab_validation
1216	Positive	16.40	
1217	Negative		
1218	Negative		Lab_validation
1219	Negative		Lab_validation
1220	Negative		Lab_validation
1221	Negative		
1222	Negative		
1225	Negative		
1226	Negative		
1227	Negative		Lab_validation
1228	Positive	19.74	
1229	Negative		
1230	Negative		
1231	Negative		Lab_validation
1232	Negative		
1233	Negative		
1234	Positive	21.33	Lab_validation
1235	Negative		Lab_validation
1238	Negative		Lab_validation
1240	Negative		Lab_validation
1242	Negative		
1243	Negative		
1244	Negative		Lab_validation
1246	Negative		
1247	Negative		Lab_validation
1248	Negative		
1249	Negative		Lab_validation
1250	Negative		Lab_validation
1251	Negative		
1252	Negative		
1253	Negative		
1254	Positive	17.16	Lab_validation
1255	Negative		Lab_validation
1256	Negative		
1257	Negative		
1258	Positive	17.67	
1259	Negative		Lab_validation
1260	Negative		
1261	Positive	14.09	Lab_validation
1263	Positive	15.02	Lab_validation
1264	Positive	15.75	
1265	Positive	14.73	Lab_validation
1266	Positive	29.18	
1267	Positive	19.93	Lab_validation
1268	Positive	24.18	
1269	Positive	22.64	Lab_validation
1270	Positive	24.20	
1271	Positive	15.75	Lab_validation
1272	Positive	27.95	

1273	Negative		
1274	Negative		Lab_validation
1275	Negative		
1276	Negative		Lab_validation
1277	Negative		
1280	Negative		Lab_validation
1281	Positive	16.43	Lab_validation
1282	Negative		
1283	Negative		Lab_validation
1284	Negative		
1285	Positive	29.96	
1286	Negative		
1287	Negative		
1288	Negative		
1289	Negative		Lab_validation
1290	Negative		
1291	Negative		
1292	Negative		
1294	Positive	29.57	
1295	Negative		Lab_validation
1296	Negative		
1297	Negative		
1298	Negative		Lab_validation
1299	Negative		
1300	Negative		
1301	Negative		Lab_validation
1302	Negative		Lab_validation
1303	Negative		Lab_validation
1304	Negative		
1305	Negative		
1306	Positive	20.71	Lab_validation
1307	Negative		
1308	Negative		Lab_validation
1309	Positive	17.44	
1310	Negative		Lab_validation
1311	Negative		
1312	Positive	30.59	
1313	Positive	19.09	Lab_validation
1314	Positive	15.38	
1315	Negative		
1317	Positive	26.41	
1318	Negative		
1319	Negative		Lab_validation
1320	Negative		
1321	Negative		
1322	Negative		
1323	Negative		Lab_validation
1324	Negative		
1325	Negative		
1327	Negative		Lab_validation

1328	Negative		
1329	Negative		Lab_validation
1330	Negative		
1331	Negative		
1332	Negative		
1333	Negative		Lab_validation
1334	Positive	31.09	Lab_validation
1335	Negative		
1336	Negative		
1337	Negative		
1338	Negative		Lab_validation
1339	Positive	29.00	
1340	Negative		
1342	Positive	20.09	Lab_validation
1343	Negative		Lab_validation
1346	Positive	33.04	
1347	Positive	28.71	Lab_validation
1349	Positive	34.99	
1350	Positive	31.20	
1351	Positive	28.87	Lab_validation
1352	Positive	27.96	
1353	Positive	28.03	
1354	Negative		
1356	Positive	34.63	
1357	Negative		Lab_validation
1358	Negative		
1359	Negative		
1360	Negative		
1361	Negative		
1362	Negative		
1363	Negative		Lab_validation
1364	Negative		
1365	Negative		
1366	Negative		
1367	Negative		Lab_validation
1368	Negative		
1369	Negative		
1370	Negative		
1371	Negative		Lab_validation
1372	Negative		
1373	Positive	33.17	Lab_validation
1374	Negative		
1375	Negative		
1376	Negative		Lab_validation
1377	Negative		
1378	Negative		
1379	Negative		
1380	Negative		Lab_validation
1382	Negative		Lab_validation
1383	Positive	16.20	Field_validation

1384	Positive	23.31	
1385	Positive	18.12	Field_validation
1386	Positive	20.30	Lab_validation
1387	Positive	15.34	
1388	Positive	17.38	Field_validation
1389	Positive	23.30	
1390	Positive	25.22	Field_validation
1391	Positive	29.64	
1392	Positive	31.35	Field_validation
1393	Positive	21.64	
1394	Positive	23.54	Lab_validation
1395	Negative		Lab_validation
1396	Negative		Lab_validation
1397	Positive	19.47	
1398	Positive	14.83	
1400	Positive	32.58	
1401	Negative		
1403	Negative		
1404	Positive	34.19	Lab_validation
1405	Positive	27.95	
1406	Negative		
1407	Positive	18.31	
1408	Negative		
1409	Positive	21.11	
1411	Negative		
1412	Negative		Lab_validation
1413	Negative		
1414	Negative		
1415	Negative		Lab_validation
1416	Negative		
1417	Negative		
1418	Positive	19.13	Lab_validation
1419	Positive	17.17	
1421	Positive	33.12	
1422	Positive	22.15	
1423	Positive	34.19	
1424	Negative		
1425	Positive	13.54	
1426	Positive	29.34	
1427	Positive	29.63	Lab_validation
1428	Positive	29.66	
1429	Negative		Lab_validation
1431	Negative		
1432	Negative		
1433	Positive	24.20	
1434	Positive	32.58	
1435	Negative		Lab_validation
1437	Positive	32.41	
1438	Negative		
1439	Positive	33.77	Lab_validation

1440	Positive	34.20	
1441	Positive	15.16	
1444	Positive	22.73	
1445	Positive	27.46	
1446	Positive	33.59	Lab_validation
1447	Positive	22.16	
1448	Positive	32.05	
1449	Negative		
1450	Negative		Lab_validation
1451	Negative		
1452	Negative		Lab_validation
1453	Negative		Lab_validation
1454	Negative		
1456	Negative		
1457	Negative		
1458	Positive	20.58	
1459	Positive	33.27	
1461	Negative		Lab_validation
1462	Negative		
1463	Negative		
1464	Negative		
1465	Positive	13.92	Lab_validation
1466	Positive	31.50	
1468	Negative		
1469	Negative		
1470	Negative		
1471	Negative		Lab_validation
1472	Positive	34.32	
1473	Negative		
1475	Negative		
1476	Negative		Lab_validation
1477	Negative		Lab_validation
1478	Positive	19.58	Lab_validation
1479	Negative		
1480	Negative		
1481	Negative		
1482	Negative		
1483	Negative		
1484	Negative		Lab_validation
1486	Negative		
1487	Negative		
1488	Negative		Lab_validation
1489	Negative		Lab_validation
1490	Negative		
1492	Negative		
1493	Negative		
1494	Negative		
1495	Negative		
1496	Negative		
1497	Negative		Lab_validation

1498	Positive	32.05	
1501	Negative		
1502	Positive	29.40	
1503	Positive	33.13	Lab_validation
1504	Positive	33.56	
1505	Positive	32.69	
1507	Positive	33.97	Lab_validation
1508	Negative		
1509	Negative		
1510	Negative		Lab_validation
1512	Negative		
1513	Negative		
1514	Positive	23.52	
1516	Negative		
1517	Negative		
1518	Negative		Lab_validation
1519	Positive	21.71	
1520	Negative		
1521	Negative		
1522	Negative		Lab_validation
1523	Negative		
1524	Negative		
1525	Negative		Lab_validation
1527	Negative		
1528	Negative		
1529	Negative		
1530	Negative		Lab_validation
1531	Negative		
1532	Negative		
1533	Negative		
1534	Negative		Lab_validation
1537	Negative		
1538	Positive	14.78	Lab_validation
1539	Negative		
1540	Positive	14.05	
1541	Negative		Lab_validation
1543	Negative		
1546	Negative		
1547	Positive	23.68	
1548	Positive	18.27	
1549	Positive	20.51	
1550	Negative		Lab_validation
1551	Positive	33.00	Lab_validation
1552	Negative		
1553	Negative		
1554	Negative		
1555	Negative		Lab_validation
1557	Negative		
1558	Negative		Lab_validation
1559	Negative		

1560	Negative		
1561	Negative		
1562	Negative		Lab_validation
1563	Negative		
1564	Negative		
1565	Negative		
1566	Positive	18.12	
1567	Positive	28.72	
1568	Positive	27.12	
1569	Negative		Lab_validation
1571	Negative		
1572	Positive	9.08	
1574	Positive	14.02	Lab_validation
1575	Negative		
1576	Negative		Lab_validation
1578	Negative		
1580	Positive	17.85	
1581	Negative		
1582	Positive	15.24	Lab_validation
1583	Positive	32.05	
1584	Positive	16.52	
1585	Positive	20.94	Lab_validation
1586	Positive	16.70	
1587	Positive	14.16	
1588	Positive	16.12	
1589	Positive	21.11	
1590	Positive	9.08	
1591	Positive	21.49	Lab_validation
1593	Positive	19.03	
1594	Positive	15.08	
1596	Positive	16.04	
1597	Positive	13.16	Lab_validation
1598	Positive	21.23	
1599	Positive	17.46	
1600	Positive	18.08	Lab_validation
1601	Positive	32.66	
1603	Negative		Lab_validation
1604	Negative		
1605	Negative		
1606	Negative		
1607	Positive	32.20	
1608	Negative		Lab_validation
1612	Positive	32.76	
1613	Negative		
1614	Negative		
1616	Negative		
1617	Negative		
1618	Positive	28.84	
1619	Positive	21.68	Lab_validation
1620	Positive	34.47	

1621	Negative		
1623	Positive	29.35	
1625	Positive	28.37	
1627	Negative		
1628	Negative		
1629	Negative		
1630	Positive	26.89	
1631	Positive	27.01	
1633	Positive	33.04	
1634	Negative		
1635	Negative		
1636	Negative		Lab_validation
1637	Negative		
1638	Negative		
1639	Negative		
1641	Negative		
1642	Negative		
1643	Negative		
1644	Negative		
1646	Negative		
1647	Negative		
1648	Positive	29.72	
1649	Negative		
1650	Negative		
1651	Negative		
1652	Positive	31.22	
1653	Positive	24.90	Lab_validation
1654	Negative		
1655	Negative		
1657	Negative		
1658	Negative		
1659	Positive	33.41	
1660	Positive	23.55	
1661	Positive	24.37	
1662	Positive	14.45	
1663	Positive	16.63	
1664	Positive	20.32	
1665	Positive	33.41	
1666	Positive	18.11	Lab_validation
1667	Positive	21.36	
1668	Positive	25.60	
1669	Positive	22.11	
1670	Positive	23.10	
1671	Negative		
1672	Positive	33.90	
1673	Negative		
1674	Negative		
1675	Positive	21.59	
1676	Positive	29.33	Lab_validation
1677	Positive	17.51	

1678	Positive	17.56	
1679	Positive	23.80	
1680	Positive	20.87	
1681	Positive	34.46	
1682	Negative		
1683	Positive	16.00	Lab_validation
1685	Positive	19.15	
1686	Positive	34.92	
1687	Positive	17.08	
1688	Positive	23.30	Lab_validation
1690	Positive	19.67	
1691	Positive	20.93	
1692	Positive	17.57	
1693	Positive	27.49	
1694	Positive	17.57	
1695	Positive	23.18	
1696	Positive	17.14	Lab_validation
1697	Positive	23.13	
1698	Positive	24.12	
1699	Negative		
1700	Positive	17.49	
1701	Negative		Lab_validation
1703	Positive	30.59	
1705	Positive	17.67	
1706	Positive	27.05	Lab_validation
1707	Positive	20.86	
1708	Positive	23.12	
1709	Positive	18.94	
1710	Positive	24.14	
1711	Positive	17.45	
1712	Positive	15.88	Lab_validation
1713	Positive	18.83	
1714	Positive	19.72	
1716	Positive	21.62	
1717	Positive	16.20	
1718	Positive	21.34	
1719	Positive	19.09	
1720	Positive	20.16	
1721	Positive	17.17	Lab_validation
1722	Positive	20.57	
1723	Positive	20.75	
1724	Positive	17.59	
1725	Positive	30.09	
1726	Positive	20.62	
1727	Positive	17.72	Lab_validation
1728	Positive	20.02	
1729	Positive	18.74	
1730	Positive	28.66	
1733	Positive	17.66	
1734	Positive	22.95	Lab_validation

1735	Positive	31.03	
1737	Positive	23.30	
1738	Positive	18.41	
1739	Positive	18.39	
1740	Positive	32.49	
1742	Positive	18.30	
1743	Positive	23.81	
1744	Positive	21.27	
1745	Positive	22.13	Lab_validation
1746	Positive	22.27	
1747	Positive	17.48	
1750	Positive	18.37	
1751	Positive	21.97	
1753	Positive	17.37	
1754	Positive	21.38	
1755	Positive	17.82	
1756	Positive	20.33	
1757	Positive	26.27	
1758	Positive	19.57	Lab_validation
1759	Positive	22.40	
1761	Positive	30.21	
1762	Positive	17.25	
1763	Positive	21.76	
1764	Positive	23.16	
1765	Positive	18.36	
1766	Positive	28.78	Lab_validation
1767	Positive	18.44	
1768	Positive	19.94	
1769	Positive	18.94	
1770	Positive	20.41	
1771	Positive	19.98	
1772	Positive	17.01	
1773	Positive	20.76	Lab_validation
1774	Positive	21.89	
1775	Positive	21.74	
1776	Positive	20.89	
1778	Positive	20.86	
1779	Positive	27.14	
1780	Positive	22.06	
1781	Positive	20.59	Lab_validation
1782	Positive	19.11	
1783	Positive	29.34	
1784	Positive	22.06	
1785	Negative		
1787	Positive	22.84	
1788	Positive	19.03	
1789	Positive	21.02	Lab_validation
1791	Positive	19.41	
1792	Positive	30.65	
1793	Positive	28.39	

1794	Positive	28.24	
1795	Positive	19.75	
1796	Positive	21.40	
1798	Negative		
1800	Positive	21.06	
1801	Positive	19.59	
1802	Positive	24.97	
1803	Positive	21.47	Lab_validation
1804	Negative		
1806	Negative		
1807	Negative		
1808	Positive	24.64	
1809	Positive	23.00	
1810	Positive	20.23	
1811	Positive	21.07	
1812	Positive	33.24	
1813	Positive	20.49	Lab_validation
1814	Positive	20.01	
1815	Positive	17.47	
1818	Positive	23.23	Lab_validation
1819	Positive	16.71	
1820	Positive	17.37	
1821	Positive	22.04	
1822	Positive	32.92	
1823	Positive	20.19	Lab_validation
1824	Positive	19.25	
1825	Positive	27.49	
1826	Negative		
1827	Positive	22.28	
1828	Positive	19.53	
1829	Positive	25.05	
1831	Negative		
1832	Negative		
1833	Positive	32.17	Lab_validation
1834	Positive	20.27	
1836	Positive	19.08	
1837	Positive	26.23	Lab_validation
1838	Positive	24.11	
1839	Positive	17.08	Lab_validation
1840	Positive	19.18	
1841	Positive	24.38	
1842	Positive	21.50	
1843	Positive	18.74	Lab_validation
1844	Positive	26.19	
1845	Positive	18.83	
1846	Positive	16.55	
1847	Positive	30.26	
1848	Positive	21.33	
1849	Positive	26.05	Lab_validation
1850	Positive	25.11	

1851	Positive	24.78	
1852	Positive	18.96	
1853	Positive	20.11	
1855	Positive	20.16	Lab_validation
1856	Positive	21.32	
1857	Positive	17.17	
1858	Positive	19.51	
1859	Positive	18.60	
1860	Positive	20.36	
1861	Positive	26.36	
1863	Positive	16.34	
1864	Positive	26.01	
1865	Positive	23.29	
1866	Positive	26.75	
1867	Positive	21.45	
1869	Positive	28.71	Lab_validation
1870	Positive	22.10	
1871	Positive	23.53	
1872	Positive	23.43	
1873	Positive	18.29	
1874	Positive	23.52	
1875	Positive	28.78	
1877	Positive	19.83	Lab_validation
1878	Positive	32.39	
1879	Positive	18.74	
1880	Positive	21.72	
1882	Positive	21.04	
1883	Positive	19.12	
1884	Positive	24.78	
1885	Positive	20.82	
1886	Positive	22.42	Lab_validation
1887	Positive	25.03	
1889	Positive	26.36	
1890	Positive	19.95	
1893	Positive	22.13	Lab_validation
1894	Positive	16.25	
1895	Positive	22.89	
1896	Positive	19.87	
1897	Positive	23.42	
1898	Positive	27.54	
1899	Positive	23.18	
1900	Positive	30.38	Lab_validation
1901	Positive	26.31	
1902	Positive	24.09	
1904	Positive	25.28	
1905	Positive	19.80	
1906	Positive	22.58	
1907	Positive	22.80	
1908	Positive	22.47	
1909	Positive	18.11	

1910	Positive	20.97	
1911	Positive	16.68	
1912	Positive	30.51	
1913	Positive	22.13	Lab_validation
1914	Negative		
1915	Positive	19.64	
1916	Positive	23.60	
1918	Positive	23.10	
1919	Positive	11.52	
1920	Positive	32.52	
1922	Negative		
1923	Positive	31.62	Lab_validation
1924	Positive	18.29	
1925	Positive	17.05	
1926	Positive	17.68	
1927	Positive	20.07	
1928	Negative		
1929	Negative		Lab_validation
1930	Positive	14.08	
1931	Positive	17.15	Lab_validation
1933	Positive	18.50	
1935	Positive	25.28	
1937	Positive	29.51	
1939	Positive	17.46	
1940	Positive	13.89	
1942	Positive	21.01	
1943	Positive	25.35	
1945	Positive	22.35	Lab_validation
1946	Positive	22.64	
1947	Negative		
1948	Positive	21.37	
1949	Positive	18.47	
1950	Positive	20.66	
1951	Positive	24.71	
1953	Positive	15.58	
1954	Positive	19.21	
1955	Positive	26.04	Lab_validation
1956	Positive	19.55	
1958	Positive	18.71	
1959	Positive	16.18	
1960	Negative		
1961	Positive	20.42	
1962	Positive	21.82	
1963	Positive	31.88	Lab_validation
1964	Positive	23.78	
1965	Negative		Lab_validation
1966	Positive	16.22	
1967	Positive	31.68	
1968	Positive	31.05	
1969	Positive	20.15	

1970	Positive	22.54	Lab_validation
1971	Positive	28.21	
1972	Negative		
1973	Negative		
1974	Positive	18.06	
1975	Positive	26.79	Lab_validation
1976	Positive	19.80	
1977	Positive	19.43	
1978	Positive	19.79	
1979	Positive	17.86	
1980	Positive	28.22	
1981	Positive	23.32	
1982	Positive	26.47	Lab_validation
1983	Positive	24.99	
1984	Positive	20.40	
1985	Positive	15.19	
1986	Positive	18.07	
1987	Positive	23.25	
1988	Positive	20.26	
1989	Positive	17.79	
1990	Positive	25.20	Lab_validation
1991	Positive	28.21	
1992	Positive	25.20	
1993	Positive	31.10	
1994	Positive	21.82	
1995	Positive	22.29	
1996	Positive	20.23	
1997	Positive	23.27	
1999	Positive	17.48	Lab_validation
2001	Positive	19.95	
2003	Positive	19.04	
2004	Positive	18.15	
2005	Positive	18.55	Lab_validation
2007	Positive	23.02	
2008	Positive	23.48	
2009	Positive	18.06	
2010	Positive	19.25	
2011	Positive	24.32	
2012	Positive	17.80	
2013	Positive	32.05	Lab_validation
2014	Positive	18.21	Lab_validation
2015	Positive	21.24	Lab_validation
2016	Positive	26.24	
2018	Positive	34.12	
2019	Positive	20.46	
2020	Positive	17.81	
2021	Positive	28.99	Lab_validation
2022	Positive	20.04	
2023	Positive	30.16	
2024	Positive	16.30	

2025	Positive	20.94	
2026	Positive	19.73	Lab_validation
2027	Positive	23.00	
2029	Positive	19.89	
2030	Positive	29.80	
2031	Positive	16.12	
2032	Positive	23.95	
2033	Positive	16.73	Lab_validation
2034	Positive	23.56	
2035	Positive	16.33	
2036	Negative		
2037	Positive	19.86	
2038	Positive	28.15	
2039	Positive	25.30	
2040	Positive	17.58	Lab_validation
2041	Positive	15.88	
2042	Positive	18.88	
2043	Positive	24.23	
2044	Positive	20.17	
2045	Positive	28.34	
2046	Positive	18.61	Lab_validation
2047	Positive	18.59	
2048	Positive	19.10	
2049	Positive	31.78	
2050	Positive	24.99	
2051	Positive	30.75	
2052	Positive	18.28	
2053	Positive	18.46	Lab_validation
2054	Positive	30.69	
2055	Positive	25.58	
2056	Positive	21.43	
2057	Positive	29.62	
2059	Positive	15.89	Lab_validation
2060	Positive	16.81	
2061	Positive	18.02	
2063	Positive	19.17	
2064	Positive	22.63	Lab_validation
2065	Positive	17.05	
2066	Positive	19.72	
2067	Positive	20.79	
2068	Positive	18.73	Lab_validation
2069	Positive	20.47	
2070	Positive	18.24	
2071	Positive	21.13	
2072	Positive	21.01	
2073	Positive	28.13	
2074	Positive	18.67	
2075	Positive	21.33	
2076	Positive	18.88	
2077	Positive	18.70	

2078	Positive	16.28	
2079	Positive	18.28	Lab_validation
2081	Negative		Lab_validation
2082	Positive	24.19	
2083	Positive	17.39	
2084	Positive	17.52	
2085	Positive	19.14	Lab_validation
2087	Positive	17.00	
2088	Positive	21.22	
2089	Positive	26.04	
2090	Positive	28.79	Lab_validation
2091	Positive	17.20	
2093	Positive	20.73	
2094	Positive	18.24	
2095	Negative		
2096	Positive	32.94	
2097	Positive	22.21	
2098	Positive	20.74	Lab_validation
2099	Positive	19.38	
2100	Positive	17.23	
2101	Positive	31.32	
2103	Positive	15.21	
2104	Positive	24.48	
2105	Positive	30.71	Lab_validation
2106	Positive	22.10	
2108	Positive	30.53	
2109	Positive	21.48	
2110	Positive	31.32	Lab_validation
2111	Positive	25.45	
2112	Positive	28.81	
2113	Positive	28.66	
2114	Negative		
2115	Positive	14.35	Lab_validation
2116	Positive	20.25	
2117	Positive	23.25	
2118	Positive	27.23	
2119	Positive	23.69	Lab_validation
2120	Positive	22.19	
2121	Positive	22.47	
2123	Positive	19.13	Lab_validation
2124	Positive	34.50	
2125	Positive	18.22	
2126	Positive	20.02	Lab_validation
2127	Positive	15.89	
2128	Positive	29.22	
2129	Positive	19.20	Lab_validation
2130	Positive	27.04	
2131	Positive	14.49	
2132	Positive	19.60	
2133	Positive	22.06	Lab_validation

2134	Positive	20.52	
2135	Positive	16.86	
2136	Negative		Lab_validation
2137	Positive	16.38	
2138	Positive	20.29	Lab_validation
2139	Positive	17.65	
2140	Positive	17.05	
2141	Positive	33.59	Lab_validation
2142	Positive	23.01	
2143	Positive	18.56	
2144	Positive	29.30	Lab_validation
2145	Positive	32.19	
2146	Positive	20.94	
2147	Positive	19.25	
2148	Positive	28.08	Lab_validation
2149	Negative		
2150	Positive	18.25	
2151	Positive	19.38	
2153	Positive	22.49	Lab_validation
2154	Positive	22.89	
2156	Positive	22.26	Lab_validation
2157	Positive	19.20	
2158	Positive	19.16	
2159	Positive	20.28	
2160	Positive	22.43	Lab_validation
2161	Positive	23.22	
2162	Positive	21.89	
2163	Positive	19.52	Lab_validation
2164	Positive	19.77	
2165	Positive	22.53	
2166	Positive	25.58	Lab_validation
2167	Positive	25.51	
2168	Positive	25.01	
2169	Positive	20.40	
2170	Positive	18.16	Lab_validation
2171	Positive	20.48	
2172	Positive	19.40	Lab_validation
2173	Positive	25.10	
2174	Positive	28.67	
2175	Positive	24.62	
2176	Positive	18.27	
2177	Positive	22.07	Lab_validation
2178	Positive	17.68	
2179	Positive	15.65	Lab_validation
2180	Positive	21.93	
2181	Positive	29.04	
2182	Positive	21.90	
2183	Positive	28.50	Lab_validation
2184	Positive	18.29	
2185	Positive	34.17	

2186	Positive	25.04	
2187	Positive	18.00	
2188	Positive	32.26	
2189	Positive	31.12	Lab_validation
2190	Positive	21.04	
2192	Positive	31.88	
2193	Positive	21.37	
2194	Positive	19.85	Lab_validation
2195	Positive	34.75	
2196	Positive	19.22	
2198	Positive	20.52	Lab_validation
2199	Positive	15.72	
2200	Positive	19.67	
2201	Positive	19.24	
2203	Positive	21.37	
2204	Positive	25.08	Lab_validation
2205	Positive	31.58	
2206	Positive	23.78	
2207	Positive	28.21	
2208	Positive	21.41	Lab_validation
2209	Negative		
2210	Positive	32.24	
2211	Positive	28.87	
2212	Positive	22.25	
2213	Positive	23.84	Lab_validation
2214	Positive	27.62	
2215	Positive	19.01	
2216	Negative		
2218	Positive	21.58	Lab_validation
2219	Positive	25.19	
2220	Positive	23.35	
2223	Positive	17.90	
2224	Positive	17.26	Lab_validation
2227	Positive	34.14	
2228	Positive	28.75	
2229	Negative		
2230	Positive	21.44	
2231	Positive	34.39	Lab_validation
2232	Negative		Lab_validation
2233	Positive	17.80	
2234	Positive	29.14	
2235	Positive	26.52	
2236	Positive	18.23	
2237	Positive	25.83	
2238	Positive	27.09	
2239	Positive	19.02	Lab_validation
2240	Positive	18.98	
2241	Positive	30.01	
2242	Positive	23.64	
2243	Positive	26.87	

2244	Positive	19.60	
2245	Negative		
2246	Positive	22.17	
2247	Positive	20.33	
2248	Positive	29.18	
2249	Positive	20.06	Lab_validation
2250	Positive	21.62	
2251	Positive	34.83	
2252	Positive	21.38	
2253	Negative		
2254	Positive	34.42	
2255	Positive	24.63	
2256	Negative		
2257	Positive	21.66	Lab_validation
2258	Positive	22.43	
2259	Positive	18.10	
2260	Positive	18.23	
2261	Positive	28.04	
2262	Positive	33.99	
2263	Positive	18.04	Lab_validation
2264	Positive	30.54	
2266	Positive	25.22	
2267	Negative		
2268	Positive	18.71	
2269	Positive	27.47	
2270	Positive	19.27	
2271	Positive	30.52	Lab_validation
2272	Negative		Lab_validation
2273	Positive	22.42	
2274	Positive	17.95	
2275	Positive	22.58	
2276	Positive	19.56	
2277	Positive	19.78	
2278	Positive	21.21	
2279	Positive	19.27	
2280	Positive	17.09	
2281	Positive	20.66	Lab_validation
2282	Positive	18.60	
2283	Positive	29.91	
2285	Positive	19.59	
2286	Positive	22.22	
2287	Positive	23.25	Lab_validation
2288	Positive	16.51	
2289	Positive	21.91	
2290	Positive	20.17	
2292	Positive	16.50	
2294	Negative		Lab_validation
2295	Positive	18.66	Lab_validation
2296	Positive	18.30	
2297	Negative		

2298	Positive	16.39	
2299	Positive	29.45	Lab_validation
2300	Positive	19.25	
2301	Positive	22.99	
2303	Positive	20.99	
2304	Negative		
2305	Positive	31.02	Lab_validation
2306	Positive	32.63	
2307	Positive	32.21	
2308	Positive	32.58	
2309	Positive	22.12	Lab_validation
2310	Positive	26.16	
2313	Positive	16.78	Field_validation
2314	Positive	29.10	Lab_validation
2315	Negative		Field_validation
2316	Negative		
2317	Positive	19.76	Field_validation
2318	Positive	22.56	
2319	Negative		Field_validation
2320	Positive	29.82	Field_validation
2321	Positive	26.06	Lab_validation
2322	Positive	30.33	Field_validation
2323	Positive	27.12	Field_validation
2324	Negative		Field_validation
2325	Negative		Field_validation
2326	Positive	28.75	Field_validation
2327	Positive	20.83	Field_validation
2328	Positive	23.02	
2329	Positive	24.58	Field_validation
2330	Positive	24.12	Lab_validation
2331	Positive	29.42	
2332	Positive	18.58	Field_validation
2333	Positive	24.79	
2334	Negative		Lab_validation
2335	Positive	20.05	Lab_validation
2336	Positive	28.39	Field_validation
2337	Positive	28.62	
2338	Positive	26.12	Field_validation
2339	Positive	17.68	Field_validation
2340	Positive	23.37	Field_validation
2341	Positive	24.49	Lab_validation
2342	Positive	29.12	Field_validation
2343	Positive	27.23	
2344	Positive	25.51	Field_validation
2345	Positive	25.34	Field_validation
2347	Positive	19.57	Field_validation
2348	Positive	28.03	Field_validation
2349	Positive	22.80	Field_validation
2350	Negative		Field_validation
2351	Positive	22.35	Field_validation

2352	Positive	26.40	Lab_validation
