# Supplementary material for: Validation of new equipment for SARS-CoV-2 diagnosis in Ecuador: Detection of the virus and antibodies generated by disease and vaccines with one POC device
Source: PLoS One. 2025 Apr 16;20(4):e0321794. doi: 10.1371/journal.pone.0321794 (PMC12002511; doi:10.1371/journal.pone.0321794)
Supplement: S2 File — (PDF) [file pone.0321794.s002.pdf]

SAMPLE	PLATE	SARS-CoV-2 (TTR)	IC (TTR)	RT-qPCR_E	PLUM RT-LAMP	VALIDATION
2	1	52.4	41.5	POS	NEG	False_Neg
3	1	52.4	41.5	NEG	NEG	True_Neg
4	1	42.4	36.5	POS	NEG	False_Neg
5	1	52.4	41.5	NEG	NEG	True_Neg
6	1	35.4	34.5	POS	POS	True_Pos
7	1	52.4	41.5	NEG	NEG	True_Neg
8	1	40.4	35.5	POS	POS	True_Pos
9	1	52.4	34.5	NEG	NEG	True_Neg
10	1	34.4	30.5	POS	POS	True_Pos
11	8	41.4	37.5	NEG	NEG	True_Neg
13	1	52.4	42.5	NEG	NEG	True_Neg
14	1	52.4	38.5	NEG	NEG	True_Neg
15	1	52.4	41.5	NEG	NEG	True_Neg
16	1	39.4	39.5	POS	POS	True_Pos
17	1	52.4	38.5	NEG	NEG	True_Neg
18	1	37.4	34.5	POS	POS	True_Pos
19	1	52.4	39.5	NEG	NEG	True_Neg
20	1	36.4	32.5	POS	POS	True_Pos
21	1	52.4	41.5	NEG	NEG	True_Neg
22	1	33.4	33.5	POS	POS	True_Pos
23	8	41.4	38.5	NEG	NEG	True_Neg
24	1	52.4	44.5	POS	NEG	False_Neg
25	1	52.4	43.5	NEG	NEG	True_Neg
26	1	42.4	39.5	POS	NEG	False_Neg
27	1	52.4	43.5	NEG	NEG	True_Neg
28	1	41.4	42.5	POS	NEG	False_Neg
29	1	52.4	43.5	NEG	NEG	True_Neg
30	1	34.4	33.5	POS	POS	True_Pos
32	1	40.4	32.5	POS	POS	True_Pos
33	1	52.4	32.5	NEG	NEG	True_Neg
34	1	36.4	34.5	POS	POS	True_Pos
35	1	52.4	7.5	NEG	NEG	True_Neg
36	8	41.4	31.5	POS	NEG	False_Neg
37	1	52.4	41.5	NEG	NEG	True_Neg
38	1	40.4	37.5	POS	POS	True_Pos
39	1	52.4	23.5	NEG	NEG	True_Neg
40	1	45.4	35.5	POS	NEG	False_Neg
41	1	52.4	32.5	NEG	NEG	True_Neg
42	1	52.4	34.5	POS	NEG	False_Neg
43	1	52.4	33.5	NEG	NEG	True_Neg
44	8	41.4	28.5	POS	NEG	False_Neg
45	8	41.4	28.5	NEG	NEG	True_Neg
47	2	54.4	40.5	NEG	NEG	True_Neg
48	2	41.4	41.5	POS	NEG	False_Neg
49	10	55.4	35.5	NEG	NEG	True_Neg
50	2	54.4	38.5	POS	NEG	False_Neg
51	2	54.4	38.5	NEG	NEG	True_Neg
52	2	54.4	35.5	POS	NEG	False_Neg

53	2	54.4	40.5	NEG	NEG	True_Neg
54	2	30.4	32.5	POS	POS	True_Pos
55	2	54.4	30.5	NEG	NEG	True_Neg
56	2	38.4	36.5	POS	POS	True_Pos
57	2	54.4	46.5	NEG	NEG	True_Neg
58	2	40.4	44.5	POS	POS	True_Pos
59	2	54.4	40.5	NEG	NEG	True_Neg
60	2	35.4	40.5	POS	POS	True_Pos
61	2	54.4	7.5	NEG	NEG	True_Neg
62	2	38.4	8.5	POS	POS	True_Pos
63	2	54.4	8.5	NEG	NEG	True_Neg
64	2	29.4	31.5	POS	POS	True_Pos
65	2	54.4	35.5	NEG	NEG	True_Neg
66	2	28.4	24.5	POS	POS	True_Pos
67	2	54.4	31.5	NEG	NEG	True_Neg
68	2	29.4	29.5	POS	POS	True_Pos
69	2	54.4	16.5	NEG	NEG	True_Neg
70	2	39.4	47.5	POS	POS	True_Pos
71	2	54.4	42.5	NEG	NEG	True_Neg
72	2	42.4	43.5	POS	NEG	False_Neg
73	2	54.4	40.5	NEG	NEG	True_Neg
74	2	26.4	23.5	POS	POS	True_Pos
75	2	54.4	8.5	NEG	NEG	True_Neg
76	2	27.4	36.5	POS	POS	True_Pos
77	10	55.4	36.5	NEG	NEG	True_Neg
78	2	34.4	30.5	POS	POS	True_Pos
79	2	54.4	38.5	NEG	NEG	True_Neg
80	2	31.4	31.5	POS	POS	True_Pos
81	2	54.4	48.5	NEG	NEG	True_Neg
82	2	42.4	51.5	POS	NEG	False_Neg
83	2	54.4	42.5	NEG	NEG	True_Neg
84	8	41.4	27.5	POS	NEG	False_Neg
85	2	54.4	40.5	NEG	NEG	True_Neg
86	2	24.4	39.5	POS	POS	True_Pos
87	10	55.4	40.5	NEG	NEG	True_Neg
88	2	28.4	34.5	POS	POS	True_Pos
89	10	40.4	38.5	NEG	POS	False_Pos
90	2	25.4	31.5	POS	POS	True_Pos
91	10	55.4	37.5	NEG	NEG	True_Neg
92	2	54.4	32.5	POS	NEG	False_Neg
93	3	54.4	43.5	NEG	NEG	True_Neg
94	3	51.4	41.5	POS	NEG	False_Neg
95	3	51.4	43.5	NEG	NEG	True_Neg
96	3	33.4	39.5	POS	POS	True_Pos
97	3	51.4	37.5	NEG	NEG	True_Neg
98	3	33.4	37.5	POS	POS	True_Pos
99	3	51.4	40.5	NEG	NEG	True_Neg
100	3	31.4	33.5	POS	POS	True_Pos
101	3	51.4	38.5	NEG	NEG	True_Neg
102	3	30.4	32.5	POS	POS	True_Pos

104	3	51.4	41.5	POS	NEG	False_Neg
105	10	55.4	33.5	NEG	NEG	True_Neg
106	3	31.4	37.5	POS	POS	True_Pos
108	3	29.4	13.5	POS	POS	True_Pos
109	10	55.4	34.5	NEG	NEG	True_Neg
110	8	41.4	27.5	POS	NEG	False_Neg
111	3	51.4	40.5	NEG	NEG	True_Neg
112	3	31.4	32.5	POS	POS	True_Pos
113	3	51.4	34.5	NEG	NEG	True_Neg
114	3	33.4	33.5	POS	POS	True_Pos
115	3	51.4	25.5	NEG	NEG	True_Neg
116	3	30.4	36.5	POS	POS	True_Pos
117	3	51.4	40.5	NEG	NEG	True_Neg
118	3	32.4	40.5	POS	POS	True_Pos
119	3	51.4	39.5	NEG	NEG	True_Neg
120	3	35.4	38.5	POS	POS	True_Pos
121	3	51.4	39.5	NEG	NEG	True_Neg
122	3	33.4	34.5	POS	POS	True_Pos
124	3	36.4	34.5	POS	POS	True_Pos
125	8	41.4	34.5	NEG	NEG	True_Neg
126	3	25.4	33.5	POS	POS	True_Pos
127	10	55.4	34.5	NEG	NEG	True_Neg
128	3	36.4	43.5	POS	POS	True_Pos
129	3	38.4	25.5	NEG	POS	False_Pos
130	3	51.4	28.5	POS	NEG	False_Neg
131	3	34.4	40.5	NEG	POS	False_Pos
132	3	51.4	38.5	POS	NEG	False_Neg
133	3	37.4	38.5	NEG	POS	False_Pos
134	3	51.4	35.5	POS	NEG	False_Neg
135	3	31.4	37.5	NEG	POS	False_Pos
136	3	51.4	29.5	POS	NEG	False_Neg
137	3	24.4	36.5	NEG	POS	False_Pos
138	3	15.4	35.5	POS	POS	True_Pos
139	4	53.4	43.5	NEG	NEG	True_Neg
140	4	33.4	39.5	POS	POS	True_Pos
141	4	53.4	26.5	NEG	NEG	True_Neg
142	4	36.4	39.5	POS	POS	True_Pos
143	4	53.4	33.5	NEG	NEG	True_Neg
144	4	28.4	35.5	POS	POS	True_Pos
145	4	53.4	37.5	NEG	NEG	True_Neg
146	4	31.4	34.5	POS	POS	True_Pos
147	4	53.4	40.5	NEG	NEG	True_Neg
148	4	31.4	31.5	POS	POS	True_Pos
149	8	41.4	27.5	NEG	NEG	True_Neg
150	8	33.4	30.5	POS	POS	True_Pos
151	4	53.4	41.5	NEG	NEG	True_Neg
152	4	53.4	39.5	POS	NEG	False_Neg
153	4	53.4	38.5	NEG	NEG	True_Neg
154	4	43.4	36.5	POS	NEG	False_Neg
155	4	53.4	34.5	NEG	NEG	True_Neg

156	4	30.4	32.5	POS	POS	True_Pos
157	4	53.4	32.5	NEG	NEG	True_Neg
158	4	30.4	32.5	POS	POS	True_Pos
159	4	53.4	36.5	NEG	NEG	True_Neg
160	4	29.4	34.5	POS	POS	True_Pos
161	4	53.4	50.5	NEG	NEG	True_Neg
162	8	29.4	25.5	POS	POS	True_Pos
163	4	53.4	51.5	NEG	NEG	True_Neg
164	8	26.4	27.5	POS	POS	True_Pos
165	4	53.4	50.5	NEG	NEG	True_Neg
166	4	36.4	43.5	POS	POS	True_Pos
168	4	22.4	31.5	POS	POS	True_Pos
169	10	40.4	31.5	NEG	POS	False_Pos
170	4	23.4	35.5	POS	POS	True_Pos
171	4	53.4	34.5	NEG	NEG	True_Neg
172	4	26.4	36.5	POS	POS	True_Pos
173	4	53.4	7.5	NEG	NEG	True_Neg
175	8	41.4	26.5	NEG	NEG	True_Neg
177	4	53.4	40.5	NEG	NEG	True_Neg
178	4	34.4	40.5	POS	POS	True_Pos
179	4	53.4	36.5	NEG	NEG	True_Neg
180	4	30.4	35.5	POS	POS	True_Pos
181	10	40.4	29.5	NEG	POS	False_Pos
182	4	33.4	31.5	POS	POS	True_Pos
183	10	41.4	35.5	NEG	NEG	True_Neg
184	4	31.4	34.5	POS	POS	True_Pos
185	5	37.4	39.5	NEG	POS	False_Pos
186	5	48.4	42.5	POS	NEG	False_Neg
187	5	37.4	37.5	NEG	POS	False_Pos
188	5	48.4	37.5	POS	NEG	False_Neg
189	5	48.4	39.5	NEG	NEG	True_Neg
190	5	9.4	36.5	POS	POS	True_Pos
191	10	55.4	40.5	NEG	NEG	True_Neg
192	5	35.4	36.5	POS	POS	True_Pos
193	5	48.4	37.5	NEG	NEG	True_Neg
194	5	33.4	36.5	POS	POS	True_Pos
195	10	42.4	36.5	NEG	NEG	True_Neg
196	5	34.4	35.5	POS	POS	True_Pos
197	5	48.4	42.5	NEG	NEG	True_Neg
198	5	36.4	39.5	POS	POS	True_Pos
199	5	48.4	39.5	NEG	NEG	True_Neg
200	5	48.4	33.5	POS	NEG	False_Neg
201	5	48.4	26.5	NEG	NEG	True_Neg
202	5	26.4	29.5	POS	POS	True_Pos
203	5	48.4	36.5	NEG	NEG	True_Neg
204	5	48.4	35.5	POS	NEG	False_Neg
205	5	48.4	38.5	NEG	NEG	True_Neg
206	5	48.4	25.5	POS	NEG	False_Neg
207	5	48.4	41.5	NEG	NEG	True_Neg
208	5	48.4	42.5	POS	NEG	False_Neg

210	5	35.4	41.5	POS	POS	True_Pos
211	5	48.4	31.5	NEG	NEG	True_Neg
212	5	28.4	25.5	POS	POS	True_Pos
213	5	48.4	27.5	NEG	NEG	True_Neg
214	5	29.4	32.5	POS	POS	True_Pos
215	5	48.4	34.5	NEG	NEG	True_Neg
216	5	31.4	15.5	POS	POS	True_Pos
217	5	48.4	36.5	NEG	NEG	True_Neg
218	5	27.4	10.5	POS	POS	True_Pos
219	5	48.4	42.5	NEG	NEG	True_Neg
221	8	41.4	37.5	NEG	NEG	True_Neg
223	5	48.4	43.5	NEG	NEG	True_Neg
224	5	39.4	27.5	POS	POS	True_Pos
225	5	48.4	33.5	NEG	NEG	True_Neg
226	5	32.4	39.5	POS	POS	True_Pos
227	5	48.4	6.5	NEG	NEG	True_Neg
228	5	29.4	23.5	POS	POS	True_Pos
229	5	48.4	19.5	NEG	NEG	True_Neg
230	5	18.4	11.5	POS	POS	True_Pos
231	6	51.4	19.5	NEG	NEG	True_Neg
233	6	51.4	38.5	NEG	NEG	True_Neg
234	6	51.4	42.5	POS	NEG	False_Neg
235	6	51.4	5.5	NEG	NEG	True_Neg
236	6	38.4	30.5	POS	POS	True_Pos
237	6	51.4	36.5	NEG	NEG	True_Neg
238	6	32.4	29.5	POS	POS	True_Pos
239	6	51.4	35.5	NEG	NEG	True_Neg
240	6	51.4	33.5	POS	NEG	False_Neg
241	6	51.4	13.5	NEG	NEG	True_Neg
242	6	43.4	43.5	POS	NEG	False_Neg
243	9	38.4	28.5	NEG	POS	False_Pos
244	9	28.4	27.5	POS	POS	True_Pos
245	6	51.4	6.5	NEG	NEG	True_Neg
247	6	51.4	27.5	NEG	NEG	True_Neg
248	6	33.4	28.5	POS	POS	True_Pos
249	6	51.4	37.5	NEG	NEG	True_Neg
250	6	30.4	19.5	POS	POS	True_Pos
251	10	55.4	40.5	NEG	NEG	True_Neg
252	6	27.4	27.5	POS	POS	True_Pos
253	6	51.4	8.5	NEG	NEG	True_Neg
254	9	38.4	26.5	POS	NEG	False_Neg
255	6	51.4	45.5	NEG	NEG	True_Neg
256	9	21.4	24.5	POS	POS	True_Pos
257	6	51.4	44.5	NEG	NEG	True_Neg
258	6	46.4	38.5	POS	NEG	False_Neg
259	6	51.4	31.5	NEG	NEG	True_Neg
260	6	30.4	17.5	POS	POS	True_Pos
261	6	51.4	42.5	NEG	NEG	True_Neg
262	6	39.4	7.5	POS	POS	True_Pos
263	6	51.4	39.5	NEG	NEG	True_Neg

264	6	40.4	33.5	POS	POS	True_Pos
265	6	51.4	4.5	NEG	NEG	True_Neg
266	6	51.4	6.5	POS	NEG	False_Neg
267	6	51.4	5.5	NEG	NEG	True_Neg
268	9	27.4	22.5	POS	POS	True_Pos
269	6	51.4	8.5	NEG	NEG	True_Neg
270	6	38.4	35.5	POS	POS	True_Pos
271	6	51.4	34.5	NEG	NEG	True_Neg
272	6	44.4	33.5	POS	NEG	False_Neg
273	6	51.4	36.5	NEG	NEG	True_Neg
274	6	32.4	7.5	POS	POS	True_Pos
275	9	38.4	33.5	NEG	POS	False_Pos
276	7	37.4	37.5	POS	POS	True_Pos
277	9	38.4	24.5	NEG	POS	False_Pos
279	7	53.4	36.5	NEG	NEG	True_Neg
281	7	53.4	30.5	NEG	NEG	True_Neg
282	7	39.4	38.5	POS	POS	True_Pos
283	7	53.4	33.5	NEG	NEG	True_Neg
284	7	35.4	35.5	POS	POS	True_Pos
285	7	53.4	31.5	NEG	NEG	True_Neg
286	7	35.4	34.5	POS	POS	True_Pos
287	7	26.4	34.5	NEG	POS	False_Pos
288	9	38.4	33.5	POS	NEG	False_Neg
289	9	38.4	29.5	NEG	POS	False_Pos
290	9	38.4	28.5	POS	NEG	False_Neg
291	7	53.4	33.5	NEG	NEG	True_Neg
292	7	53.4	37.5	POS	NEG	False_Neg
293	7	53.4	32.5	NEG	NEG	True_Neg
295	7	53.4	32.5	NEG	NEG	True_Neg
296	9	38.4	34.5	POS	NEG	False_Neg
297	7	53.4	34.5	NEG	NEG	True_Neg
298	7	32.4	30.5	POS	POS	True_Pos
299	10	43.4	30.5	NEG	NEG	True_Neg
