# Supplementary material for: Validation of new equipment for SARS-CoV-2 diagnosis in Ecuador: Detection of the virus and antibodies generated by disease and vaccines with one POC device
Source: PLoS One. 2025 Apr 16;20(4):e0321794. doi: 10.1371/journal.pone.0321794 (PMC12002511; doi:10.1371/journal.pone.0321794)
Supplement: S3 File — (PDF) [file pone.0321794.s003.pdf]

SAMPLE	PLATE	SARS-CoV-2 (TTR)	IC (TTR)	RT-qPCR_E	PLUM RT-LAMP	VALIDATION
1388	C1	34	35.33	POS	POS	True_Pos
1390	C3	50	34.33	POS	NEG	False_Neg
1392	C1	34	31.33	POS	POS	True_Pos
2313	C1	28	35.33	POS	POS	True_Pos
2315	C1	49	38.33	NEG	NEG	True_Neg
2317	C1	31	37.33	POS	POS	True_Pos
2319	C1	49	43.33	NEG	NEG	True_Neg
2320	C1	37	47.33	POS	POS	True_Pos
2322	C1	24	23.33	POS	POS	True_Pos
2323	C1	37	38.33	POS	POS	True_Pos
2325	C1	44	32.33	NEG	NEG	True_Neg
2326	C1	35	35.33	POS	POS	True_Pos
2327	C2	31	40.33	POS	POS	True_Pos
2329	C2	30	34.33	POS	POS	True_Pos
2332	C2	31	34.33	POS	POS	True_Pos
2336	C2	33	32.33	POS	POS	True_Pos
2338	C2	40	32.33	POS	POS	True_Pos
2339	C2	34	35.33	POS	POS	True_Pos
2340	C2	37	37.33	POS	POS	True_Pos
2344	C2	38	35.33	POS	POS	True_Pos
2345	C2	40	36.33	POS	POS	True_Pos
2347	C2	39	30.33	POS	POS	True_Pos
2348	C2	40	41.33	POS	POS	True_Pos
2349	C2	51	16.33	POS	NEG	False_Neg
2350	C2	51	40.33	NEG	NEG	True_Neg
2351	C2	30	31.33	POS	POS	True_Pos
T01	C1	49	38.33	NEG	NEG	True_Neg
T02	C1	32	34.33	POS	POS	True_Pos
T03	C1	49	36.33	NEG	NEG	True_Neg
T04	C1	49	35.33	NEG	NEG	True_Neg
T06	C1	49	38.33	NEG	NEG	True_Neg
T08	C2	38	34.33	NEG	POS	False_Pos
T10	C1	49	35.33	NEG	NEG	True_Neg
T11	C1	49	38.33	NEG	NEG	True_Neg
T12	C1	49	41.33	NEG	NEG	True_Neg
T14	C1	49	38.33	NEG	NEG	True_Neg
T15	C1	49	35.33	NEG	NEG	True_Neg
T17	C2	51	37.33	NEG	NEG	True_Neg
T18	C2	51	36.33	NEG	NEG	True_Neg
T20	C2	51	35.33	NEG	NEG	True_Neg
T21	C2	51	44.33	NEG	NEG	True_Neg
T22	C1	49	36.33	NEG	NEG	True_Neg
T24	C2	51	30.33	NEG	NEG	True_Neg
T25	C2	51	44.33	NEG	NEG	True_Neg
T26	C2	51	39.33	NEG	NEG	True_Neg
T27	C2	51	32.33	NEG	NEG	True_Neg
T28	C2	51	34.33	NEG	NEG	True_Neg
T29	C2	51	32.33	NEG	NEG	True_Neg

T30	C2	33	27.33	NEG	POS	False_Pos
