# Supplementary material for: Validation of new equipment for SARS-CoV-2 diagnosis in Ecuador: Detection of the virus and antibodies generated by disease and vaccines with one POC device
Source: PLoS One. 2025 Apr 16;20(4):e0321794. doi: 10.1371/journal.pone.0321794 (PMC12002511; doi:10.1371/journal.pone.0321794)
Supplement: S4 File — (PDF) [file pone.0321794.s004.pdf]

SAMPLE	PLATE	Spectrophotometer (ABS relative ratio)	SPEC Spike-RBD	PLUM (PRU relative ratio)	PLUM Spike-RBD	VALIDATION
1	PLATE 01B	0.652	NEG	0.812	NEG	True_Neg
2	PLATE 01B	0.759	NEG	0.943	NEG	True_Neg
4	PLATE 01B	0.791	NEG	0.958	NEG	True_Neg
7	PLATE 01B	0.604	NEG	0.679	NEG	True_Neg
8	PLATE 01B	0.606	NEG	0.620	NEG	True_Neg
9	PLATE 01B	0.490	NEG	0.512	NEG	True_Neg
10	PLATE 01B	0.724	NEG	0.802	NEG	True_Neg
13	PLATE 01B	0.653	NEG	0.749	NEG	True_Neg
14	PLATE 01B	0.755	NEG	0.928	NEG	True_Neg
15	PLATE 01B	0.691	NEG	0.792	NEG	True_Neg
16	PLATE 01B	0.763	NEG	0.869	NEG	True_Neg
17	PLATE 01B	0.723	NEG	0.772	NEG	True_Neg
18	PLATE 01B	0.833	NEG	0.930	NEG	True_Neg
19	PLATE 01B	0.623	NEG	0.642	NEG	True_Neg
20	PLATE 01B	0.392	NEG	0.391	NEG	True_Neg
21	PLATE 01B	1.160	NEG	1.486	NEG	True_Neg
23	PLATE 01B	1.163	NEG	1.583	NEG	True_Neg
24	PLATE 01B	0.940	NEG	1.228	NEG	True_Neg
25	PLATE 01B	0.956	NEG	1.142	NEG	True_Neg
26	PLATE 01B	0.713	NEG	0.743	NEG	True_Neg
28	PLATE 01B	0.549	NEG	0.527	NEG	True_Neg
30	PLATE 01B	0.803	NEG	0.818	NEG	True_Neg
31	PLATE 01B	0.829	NEG	0.846	NEG	True_Neg
32	PLATE 01B	0.971	NEG	1.117	NEG	True_Neg
33	PLATE 01B	0.976	NEG	1.124	NEG	True_Neg
37	PLATE 01B	0.424	NEG	0.420	NEG	True_Neg
38	PLATE 01B	1.085	NEG	1.321	NEG	True_Neg
39	PLATE 01B	0.585	NEG	0.591	NEG	True_Neg
40	PLATE 01B	0.693	NEG	0.740	NEG	True_Neg
41	PLATE 01B	0.502	NEG	0.490	NEG	True_Neg
42	PLATE 01B	1.101	NEG	1.444	NEG	True_Neg
43	PLATE 01B	0.675	NEG	0.702	NEG	True_Neg
44	PLATE 01B	0.567	NEG	0.564	NEG	True_Neg
45	PLATE 02B	0.900	NEG	1.092	NEG	True_Neg
47	PLATE 02B	1.109	NEG	1.334	NEG	True_Neg
50	PLATE 02B	0.548	NEG	0.581	NEG	True_Neg
51	PLATE 02B	0.546	NEG	0.538	NEG	True_Neg
52	PLATE 02B	0.602	NEG	0.643	NEG	True_Neg
53	PLATE 02B	0.820	NEG	0.917	NEG	True_Neg
54	PLATE 02B	1.025	NEG	1.258	NEG	True_Neg
55	PLATE 02B	0.699	NEG	0.778	NEG	True_Neg
57	PLATE 02B	0.963	NEG	1.089	NEG	True_Neg
58	PLATE 02B	0.846	NEG	0.875	NEG	True_Neg
59	PLATE 02B	0.993	NEG	1.092	NEG	True_Neg
60	PLATE 02B	0.497	NEG	0.502	NEG	True_Neg
62	PLATE 02B	0.953	NEG	0.987	NEG	True_Neg
63	PLATE 02B	0.731	NEG	0.758	NEG	True_Neg

64	PLATE 02B	0.529	NEG	0.566	NEG	True_Neg
66	PLATE 02B	0.678	NEG	0.677	NEG	True_Neg
68	PLATE 02B	1.100	NEG	1.164	NEG	True_Neg
69	PLATE 02B	0.618	NEG	0.608	NEG	True_Neg
72	PLATE 02B	0.891	NEG	0.881	NEG	True_Neg
73	PLATE 02B	0.845	NEG	0.816	NEG	True_Neg
74	PLATE 02B	0.563	NEG	0.568	NEG	True_Neg
75	PLATE 02B	1.098	NEG	1.254	NEG	True_Neg
76	PLATE 02B	0.662	NEG	0.700	NEG	True_Neg
81	PLATE 02B	0.463	NEG	0.474	NEG	True_Neg
82	PLATE 02B	0.544	NEG	0.525	NEG	True_Neg
83	PLATE 02B	1.008	NEG	1.089	NEG	True_Neg
183	PLATE 29	0.888	NEG	0.989	NEG	True_Neg
185	PLATE 05	0.235	NEG	0.306	NEG	True_Neg
187	PLATE 29	1.029	NEG	1.249	NEG	True_Neg
188	PLATE 05	0.180	NEG	0.269	NEG	True_Neg
189	PLATE 05	0.189	NEG	0.277	NEG	True_Neg
190	PLATE 29	1.301	NEG	0.819	NEG	True_Neg
191	PLATE 21	0.373	NEG	0.319	NEG	True_Neg
194	PLATE 05	0.180	NEG	0.258	NEG	True_Neg
196	PLATE 05	0.180	NEG	0.246	NEG	True_Neg
198	PLATE 21	0.594	NEG	0.706	NEG	True_Neg
199	PLATE 21	0.736	NEG	0.818	NEG	True_Neg
229	PLATE 06	0.159	NEG	0.260	NEG	True_Neg
235	PLATE 21	1.082	NEG	1.355	NEG	True_Neg
247	PLATE 21	0.717	NEG	0.782	NEG	True_Neg
248	PLATE 06	0.236	NEG	0.292	NEG	True_Neg
250	PLATE 29	1.340	NEG	2.224	POS	False_Pos
251	PLATE 21	1.324	NEG	1.337	NEG	True_Neg
252	PLATE 21	1.026	NEG	1.255	NEG	True_Neg
254	PLATE 06	0.206	NEG	0.274	NEG	True_Neg
255	PLATE 06	0.217	NEG	0.279	NEG	True_Neg
259	PLATE 29	0.492	NEG	0.412	NEG	True_Neg
264	PLATE 07	0.531	NEG	0.635	NEG	True_Neg
272	PLATE 07	1.341	NEG	1.771	POS	False_Pos
276	PLATE 07	1.016	NEG	1.134	NEG	True_Neg
293	PLATE 07	1.121	NEG	1.316	NEG	True_Neg
297	PLATE 07	0.495	NEG	0.522	NEG	True_Neg
298	PLATE 07	0.498	NEG	0.501	NEG	True_Neg
321	PLATE 08	1.340	NEG	1.760	POS	False_Pos
324	PLATE 29	0.843	NEG	0.881	NEG	True_Neg
325	PLATE 08	0.589	NEG	0.614	NEG	True_Neg
328	PLATE 08	0.621	NEG	0.655	NEG	True_Neg
330	PLATE 08	0.768	NEG	0.772	NEG	True_Neg
331	PLATE 08	0.694	NEG	0.698	NEG	True_Neg
333	PLATE 08	0.392	NEG	0.449	NEG	True_Neg
334	PLATE 08	0.613	NEG	0.658	NEG	True_Neg
335	PLATE 08	0.846	NEG	0.941	NEG	True_Neg
340	PLATE 23	0.596	NEG	0.588	NEG	True_Neg
353	PLATE 22	1.330	NEG	1.945	POS	False_Pos

359	PLATE 09	0.131	NEG	0.238	NEG	True_Neg
363	PLATE 09	0.533	NEG	0.665	NEG	True_Neg
367	PLATE 22	1.225	NEG	1.665	NEG	True_Neg
371	PLATE 22	0.934	NEG	1.050	NEG	True_Neg
408	PLATE 22	0.835	NEG	0.885	NEG	True_Neg
425	PLATE 29	0.945	NEG	1.232	NEG	True_Neg
443	PLATE 11	1.271	NEG	1.529	NEG	True_Neg
447	PLATE 11	0.389	NEG	0.317	NEG	True_Neg
457	PLATE 11	0.953	NEG	1.348	NEG	True_Neg
475	PLATE 22	1.161	NEG	1.415	NEG	True_Neg
477	PLATE 12	0.402	NEG	0.312	NEG	True_Neg
479	PLATE 22	0.657	NEG	0.675	NEG	True_Neg
481	PLATE 29	1.375	NEG	1.945	POS	False_Pos
485	PLATE 34	1.373	NEG	2.015	POS	False_Pos
489	PLATE 22	1.297	NEG	1.656	NEG	True_Neg
492	PLATE 22	0.882	NEG	0.917	NEG	True_Neg
496	PLATE 12	0.503	NEG	0.387	NEG	True_Neg
510	PLATE 24	0.462	NEG	0.470	NEG	True_Neg
522	PLATE 13	0.329	NEG	-0.370	NEG	True_Neg
525	PLATE 13	0.184	NEG	-0.009	NEG	True_Neg
539	PLATE 24	0.693	NEG	0.702	NEG	True_Neg
540	PLATE 13	0.111	NEG	-0.186	NEG	True_Neg
544	PLATE 14	0.575	NEG	1.151	NEG	True_Neg
557	PLATE 24	0.442	NEG	0.391	NEG	True_Neg
559	PLATE 24	0.451	NEG	0.367	NEG	True_Neg
560	PLATE 14	0.936	NEG	1.791	POS	False_Pos
563	PLATE 24	0.790	NEG	0.789	NEG	True_Neg
576	PLATE 14	-0.163	NEG	0.099	NEG	True_Neg
579	PLATE 24	0.609	NEG	0.636	NEG	True_Neg
588	PLATE 24	0.914	NEG	1.027	NEG	True_Neg
591	PLATE 24	0.057	NEG	0.061	NEG	True_Neg
598	PLATE 15	1.265	NEG	1.723	POS	False_Pos
602	PLATE 15	1.138	NEG	1.297	NEG	True_Neg
604	PLATE 15	0.132	NEG	-0.177	NEG	True_Neg
606	PLATE 15	1.031	NEG	1.550	NEG	True_Neg
610	PLATE 15	0.872	NEG	0.896	NEG	True_Neg
615	PLATE 25	0.597	NEG	3.708	POS	False_Pos
619	PLATE 15	0.101	NEG	-0.176	NEG	True_Neg
630	PLATE 16	0.300	NEG	0.030	NEG	True_Neg
634	PLATE 16	0.321	NEG	0.190	NEG	True_Neg
650	PLATE 25	0.969	NEG	1.899	POS	False_Pos
654	PLATE 25	0.987	NEG	1.589	NEG	True_Neg
659	PLATE 25	0.912	NEG	1.408	NEG	True_Neg
660	PLATE 16	0.651	NEG	0.630	NEG	True_Neg
686	PLATE 25	0.974	NEG	1.709	POS	False_Pos
733	PLATE 26	0.426	NEG	0.779	NEG	True_Neg
736	PLATE 18	0.450	NEG	0.455	NEG	True_Neg
743	PLATE 26	0.455	NEG	1.409	NEG	True_Neg
756	PLATE 26	0.771	NEG	1.586	NEG	True_Neg
757	PLATE 26	0.926	NEG	1.410	NEG	True_Neg

760	PLATE 26	0.635	NEG	1.578	NEG	True_Neg
762	PLATE 26	0.684	NEG	2.417	POS	False_Pos
768	PLATE 26	0.508	NEG	1.636	NEG	True_Neg
770	PLATE 19	0.928	NEG	0.937	NEG	True_Neg
781	PLATE 19	0.556	NEG	0.509	NEG	True_Neg
787	PLATE 37	1.348	NEG	1.521	NEG	True_Neg
811	PLATE 26	0.873	NEG	1.607	NEG	True_Neg
817	PLATE 26	0.978	NEG	1.578	NEG	True_Neg
820	PLATE 26	0.994	NEG	2.648	POS	False_Pos
835	PLATE 23	1.272	NEG	1.333	NEG	True_Neg
852	PLATE 37	0.960	NEG	1.142	NEG	True_Neg
11	PLATE 23	1.851	POS	2.955	POS	True_Pos
12	PLATE 23	2.229	POS	3.807	POS	True_Pos
22	PLATE 01B	2.468	POS	5.693	POS	True_Pos
27	PLATE 01B	1.856	POS	2.794	POS	True_Pos
29	PLATE 01B	2.054	POS	3.603	POS	True_Pos
34	PLATE 01B	1.659	POS	2.776	POS	True_Pos
36	PLATE 01B	2.502	POS	5.772	POS	True_Pos
46	PLATE 23	1.934	POS	2.946	POS	True_Pos
48	PLATE 29	2.003	POS	3.614	POS	True_Pos
49	PLATE 23	2.299	POS	3.767	POS	True_Pos
56	PLATE 02B	2.097	POS	3.311	POS	True_Pos
61	PLATE 02B	1.617	POS	2.176	POS	True_Pos
65	PLATE 02B	2.112	POS	3.687	POS	True_Pos
67	PLATE 02B	2.192	POS	3.397	POS	True_Pos
71	PLATE 02B	1.602	POS	2.151	POS	True_Pos
77	PLATE 02B	2.207	POS	3.627	POS	True_Pos
78	PLATE 23	1.847	POS	2.751	POS	True_Pos
80	PLATE 02B	2.618	POS	4.602	POS	True_Pos
84	PLATE 23	2.160	POS	3.781	POS	True_Pos
85	PLATE 23	2.043	POS	3.504	POS	True_Pos
86	PLATE 29	2.233	POS	5.237	POS	True_Pos
87	PLATE 29	1.796	POS	3.114	POS	True_Pos
88	PLATE 29	1.726	POS	2.903	POS	True_Pos
89	PLATE 28	2.087	POS	3.410	POS	True_Pos
90	PLATE 30	1.602	POS	3.249	POS	True_Pos
91	PLATE 32	1.450	POS	1.825	POS	True_Pos
92	PLATE 28	1.730	POS	2.594	POS	True_Pos
93	PLATE 28	1.860	POS	2.703	POS	True_Pos
94	PLATE 03A	2.153	POS	2.955	POS	True_Pos
95	PLATE 28	2.157	POS	3.304	POS	True_Pos
96	PLATE 28	2.348	POS	4.297	POS	True_Pos
97	PLATE 03	2.175	POS	5.746	POS	True_Pos
98	PLATE 29	1.945	POS	3.807	POS	True_Pos
99	PLATE 03	1.890	POS	6.100	POS	True_Pos
100	PLATE 32	1.556	POS	1.969	POS	True_Pos
101	PLATE 28	2.074	POS	3.453	POS	True_Pos
102	PLATE 03A	2.112	POS	3.411	POS	True_Pos
103	PLATE 03A	2.421	POS	4.292	POS	True_Pos
104	PLATE 32	1.943	POS	2.656	POS	True_Pos

105	PLATE 03A	2.126	POS	3.019	POS	True_Pos
106	PLATE 03	2.270	POS	5.320	POS	True_Pos
107	PLATE 03A	2.131	POS	2.992	POS	True_Pos
108	PLATE 03A	2.571	POS	3.994	POS	True_Pos
109	PLATE 28	2.612	POS	5.110	POS	True_Pos
110	PLATE 28	2.593	POS	5.916	POS	True_Pos
111	PLATE 28	2.592	POS	5.670	POS	True_Pos
112	PLATE 03	2.147	POS	6.115	POS	True_Pos
113	PLATE 03A	2.498	POS	4.675	POS	True_Pos
114	PLATE 03A	2.125	POS	3.627	POS	True_Pos
115	PLATE 03A	2.477	POS	4.294	POS	True_Pos
116	PLATE 29	2.037	POS	3.576	POS	True_Pos
117	PLATE 29	2.196	POS	4.126	POS	True_Pos
118	PLATE 03A	2.171	POS	3.352	POS	True_Pos
119	PLATE 03A	1.978	POS	2.719	POS	True_Pos
121	PLATE 28	2.599	POS	5.460	POS	True_Pos
122	PLATE 28	2.676	POS	6.267	POS	True_Pos
123	PLATE 03A	2.652	POS	4.323	POS	True_Pos
124	PLATE 28	2.676	POS	6.055	POS	True_Pos
125	PLATE 03A	2.350	POS	3.722	POS	True_Pos
126	PLATE 28	2.510	POS	4.682	POS	True_Pos
127	PLATE 03	1.966	POS	6.147	POS	True_Pos
128	PLATE 32	1.584	POS	2.017	POS	True_Pos
129	PLATE 28	2.549	POS	5.118	POS	True_Pos
130	PLATE 04	2.195	POS	5.209	POS	True_Pos
131	PLATE 29	1.542	POS	2.411	POS	True_Pos
132	PLATE 28	2.662	POS	6.212	POS	True_Pos
133	PLATE 29	1.628	POS	2.375	POS	True_Pos
134	PLATE 28	2.572	POS	5.511	POS	True_Pos
135	PLATE 37	1.689	POS	2.690	POS	True_Pos
136	PLATE 28	2.045	POS	3.695	POS	True_Pos
137	PLATE 28	1.990	POS	3.552	POS	True_Pos
138	PLATE 32	1.747	POS	2.315	POS	True_Pos
139	PLATE 37	1.866	POS	3.285	POS	True_Pos
140	PLATE 28	2.534	POS	6.971	POS	True_Pos
142	PLATE 04A	2.322	POS	3.995	POS	True_Pos
143	PLATE 33	1.980	POS	3.130	POS	True_Pos
145	PLATE 33	2.632	POS	6.235	POS	True_Pos
146	PLATE 04A	2.530	POS	4.155	POS	True_Pos
147	PLATE 33	2.011	POS	3.238	POS	True_Pos
148	PLATE 37	2.416	POS	5.892	POS	True_Pos
149	PLATE 04	2.532	POS	4.055	POS	True_Pos
150	PLATE 33	2.557	POS	4.570	POS	True_Pos
151	PLATE 04A	1.812	POS	2.504	POS	True_Pos
152	PLATE 04A	2.312	POS	3.896	POS	True_Pos
153	PLATE 04A	2.589	POS	5.482	POS	True_Pos
154	PLATE 04A	2.550	POS	4.917	POS	True_Pos
155	PLATE 33	2.186	POS	3.694	POS	True_Pos
158	PLATE 33	2.236	POS	5.209	POS	True_Pos
160	PLATE 04A	2.031	POS	2.850	POS	True_Pos

161	PLATE 04A	1.952	POS	2.582	POS	True_Pos
162	PLATE 33	1.921	POS	2.780	POS	True_Pos
163	PLATE 33	2.706	POS	4.811	POS	True_Pos
164	PLATE 37	2.594	POS	7.460	POS	True_Pos
165	PLATE 04A	1.815	POS	2.537	POS	True_Pos
166	PLATE 04A	2.165	POS	3.539	POS	True_Pos
167	PLATE 37	2.595	POS	7.707	POS	True_Pos
168	PLATE 04A	2.030	POS	3.264	POS	True_Pos
170	PLATE 04A	2.100	POS	3.352	POS	True_Pos
171	PLATE 33	2.571	POS	5.205	POS	True_Pos
172	PLATE 04A	2.039	POS	3.026	POS	True_Pos
173	PLATE 04A	2.084	POS	3.146	POS	True_Pos
174	PLATE 33	1.818	POS	2.002	POS	True_Pos
175	PLATE 33	1.732	POS	2.604	POS	True_Pos
176	PLATE 38	1.495	POS	5.577	POS	True_Pos
177	PLATE 33	1.642	POS	2.377	POS	True_Pos
178	PLATE 34	2.541	POS	4.369	POS	True_Pos
179	PLATE 29	2.199	POS	3.742	POS	True_Pos
180	PLATE 28	1.643	POS	2.709	POS	True_Pos
182	PLATE 34	1.555	POS	2.119	POS	True_Pos
186	PLATE 29	1.415	POS	1.946	POS	True_Pos
192	PLATE 21	1.758	POS	2.706	POS	True_Pos
193	PLATE 21	1.979	POS	3.305	POS	True_Pos
195	PLATE 05	1.917	POS	3.303	POS	True_Pos
197	PLATE 21	1.853	POS	3.551	POS	True_Pos
200	PLATE 05	2.157	POS	3.366	POS	True_Pos
201	PLATE 05	1.846	POS	2.724	POS	True_Pos
202	PLATE 05	2.692	POS	5.244	POS	True_Pos
203	PLATE 05	1.645	POS	2.182	POS	True_Pos
204	PLATE 21	2.012	POS	3.585	POS	True_Pos
205	PLATE 21	1.636	POS	2.358	POS	True_Pos
206	PLATE 05	2.000	POS	3.653	POS	True_Pos
207	PLATE 21	1.650	POS	2.307	POS	True_Pos
208	PLATE 05	1.616	POS	2.340	POS	True_Pos
209	PLATE 05	2.177	POS	3.596	POS	True_Pos
210	PLATE 34	2.599	POS	4.690	POS	True_Pos
211	PLATE 05	1.908	POS	2.964	POS	True_Pos
212	PLATE 05	2.359	POS	4.043	POS	True_Pos
213	PLATE 34	2.687	POS	5.315	POS	True_Pos
214	PLATE 05	1.641	POS	2.284	POS	True_Pos
215	PLATE 21	2.041	POS	3.397	POS	True_Pos
216	PLATE 06	1.523	POS	2.372	POS	True_Pos
217	PLATE 33	2.091	POS	3.299	POS	True_Pos
218	PLATE 33	1.632	POS	2.137	POS	True_Pos
219	PLATE 34	2.729	POS	5.356	POS	True_Pos
220	PLATE 38	2.451	POS	3.107	POS	True_Pos
221	PLATE 33	1.900	POS	2.703	POS	True_Pos
222	PLATE 34	2.506	POS	5.470	POS	True_Pos
223	PLATE 34	2.483	POS	5.204	POS	True_Pos
224	PLATE 06	1.564	POS	2.274	POS	True_Pos

225	PLATE 34	1.903	POS	3.170	POS	True_Pos
226	PLATE 21	2.169	POS	3.713	POS	True_Pos
227	PLATE 34	2.549	POS	5.450	POS	True_Pos
228	PLATE 06	2.629	POS	5.375	POS	True_Pos
230	PLATE 33	1.782	POS	2.523	POS	True_Pos
231	PLATE 06	2.383	POS	4.236	POS	True_Pos
232	PLATE 21	2.280	POS	4.355	POS	True_Pos
233	PLATE 38	1.799	POS	2.345	POS	True_Pos
234	PLATE 06	2.430	POS	4.754	POS	True_Pos
236	PLATE 06	1.605	POS	2.534	POS	True_Pos
237	PLATE 34	2.663	POS	5.947	POS	True_Pos
238	PLATE 06	2.184	POS	3.757	POS	True_Pos
239	PLATE 06	2.404	POS	4.333	POS	True_Pos
240	PLATE 06	1.466	POS	1.855	POS	True_Pos
241	PLATE 06	1.613	POS	2.339	POS	True_Pos
242	PLATE 34	2.708	POS	5.655	POS	True_Pos
243	PLATE 21	2.212	POS	4.484	POS	True_Pos
244	PLATE 06	2.013	POS	3.374	POS	True_Pos
245	PLATE 06	1.692	POS	2.524	POS	True_Pos
249	PLATE 06	2.464	POS	4.923	POS	True_Pos
256	PLATE 21	1.784	POS	3.187	POS	True_Pos
257	PLATE 22	1.506	POS	2.428	POS	True_Pos
258	PLATE 30	2.427	POS	2.640	POS	True_Pos
260	PLATE 29	1.586	POS	1.994	POS	True_Pos
262	PLATE 30	2.030	POS	3.504	POS	True_Pos
263	PLATE 29	1.838	POS	2.829	POS	True_Pos
265	PLATE 31	1.611	POS	2.503	POS	True_Pos
266	PLATE 27	2.225	POS	4.267	POS	True_Pos
267	PLATE 27	2.158	POS	4.569	POS	True_Pos
268	PLATE 23	1.638	POS	2.264	POS	True_Pos
269	PLATE 23	1.945	POS	2.899	POS	True_Pos
271	PLATE 23	1.995	POS	3.175	POS	True_Pos
273	PLATE 07	2.550	POS	4.347	POS	True_Pos
274	PLATE 29	2.200	POS	3.415	POS	True_Pos
275	PLATE 07	2.106	POS	3.228	POS	True_Pos
277	PLATE 07	2.140	POS	3.199	POS	True_Pos
278	PLATE 07	1.924	POS	2.810	POS	True_Pos
279	PLATE 23	2.007	POS	3.224	POS	True_Pos
280	PLATE 23	1.758	POS	2.630	POS	True_Pos
281	PLATE 07	2.580	POS	4.650	POS	True_Pos
282	PLATE 23	1.984	POS	3.453	POS	True_Pos
283	PLATE 07	2.558	POS	4.279	POS	True_Pos
284	PLATE 07	2.559	POS	4.123	POS	True_Pos
285	PLATE 07	2.100	POS	3.113	POS	True_Pos
286	PLATE 07	2.614	POS	4.500	POS	True_Pos
287	PLATE 07	2.726	POS	4.907	POS	True_Pos
288	PLATE 07	2.060	POS	3.166	POS	True_Pos
289	PLATE 07	1.646	POS	2.202	POS	True_Pos
290	PLATE 23	1.957	POS	3.222	POS	True_Pos
291	PLATE 07	2.071	POS	3.467	POS	True_Pos

292	PLATE 31	2.086	POS	3.812	POS	True_Pos
294	PLATE 31	2.049	POS	3.540	POS	True_Pos
295	PLATE 07	2.210	POS	3.533	POS	True_Pos
296	PLATE 07	2.171	POS	3.536	POS	True_Pos
300	PLATE 07	2.330	POS	4.272	POS	True_Pos
301	PLATE 27	1.561	POS	2.637	POS	True_Pos
302	PLATE 31	2.034	POS	3.152	POS	True_Pos
303	PLATE 08	1.565	POS	2.126	POS	True_Pos
304	PLATE 29	1.948	POS	3.162	POS	True_Pos
305	PLATE 08	1.788	POS	2.420	POS	True_Pos
306	PLATE 08	2.124	POS	3.138	POS	True_Pos
307	PLATE 08	2.118	POS	3.090	POS	True_Pos
308	PLATE 31	1.853	POS	2.869	POS	True_Pos
309	PLATE 27	1.526	POS	2.039	POS	True_Pos
310	PLATE 27	2.216	POS	3.816	POS	True_Pos
311	PLATE 08	2.570	POS	4.817	POS	True_Pos
312	PLATE 08	1.464	POS	2.021	POS	True_Pos
313	PLATE 08	2.668	POS	5.013	POS	True_Pos
314	PLATE 27	1.738	POS	2.530	POS	True_Pos
315	PLATE 27	2.004	POS	3.331	POS	True_Pos
316	PLATE 08	1.802	POS	2.571	POS	True_Pos
317	PLATE 08	1.667	POS	2.251	POS	True_Pos
319	PLATE 08	1.950	POS	2.975	POS	True_Pos
320	PLATE 08	1.958	POS	2.987	POS	True_Pos
322	PLATE 29	1.526	POS	2.268	POS	True_Pos
323	PLATE 08	2.437	POS	4.294	POS	True_Pos
326	PLATE 27	1.542	POS	2.311	POS	True_Pos
329	PLATE 31	2.139	POS	3.586	POS	True_Pos
332	PLATE 08	1.544	POS	2.000	POS	True_Pos
336	PLATE 29	2.022	POS	3.913	POS	True_Pos
337	PLATE 27	2.447	POS	5.520	POS	True_Pos
338	PLATE 34	2.068	POS	3.347	POS	True_Pos
339	PLATE 34	2.128	POS	3.481	POS	True_Pos
341	PLATE 31	1.531	POS	2.291	POS	True_Pos
342	PLATE 33	1.996	POS	3.085	POS	True_Pos
343	PLATE 09	1.460	POS	2.259	POS	True_Pos
344	PLATE 27	2.385	POS	5.343	POS	True_Pos
345	PLATE 32	2.533	POS	4.210	POS	True_Pos
346	PLATE 27	2.561	POS	4.864	POS	True_Pos
347	PLATE 27	2.666	POS	6.169	POS	True_Pos
348	PLATE 32	2.359	POS	3.529	POS	True_Pos
349	PLATE 27	2.297	POS	4.005	POS	True_Pos
350	PLATE 27	2.017	POS	3.369	POS	True_Pos
351	PLATE 27	2.070	POS	3.955	POS	True_Pos
352	PLATE 27	2.274	POS	4.509	POS	True_Pos
354	PLATE 22	1.563	POS	2.460	POS	True_Pos
355	PLATE 09	2.056	POS	3.579	POS	True_Pos
356	PLATE 09	1.650	POS	2.348	POS	True_Pos
357	PLATE 09	2.719	POS	4.885	POS	True_Pos
358	PLATE 29	1.961	POS	3.741	POS	True_Pos

360	PLATE 09	2.309	POS	4.034	POS	True_Pos
361	PLATE 31	2.730	POS	6.125	POS	True_Pos
362	PLATE 09	2.344	POS	4.764	POS	True_Pos
364	PLATE 09	2.103	POS	3.606	POS	True_Pos
365	PLATE 09	2.350	POS	4.275	POS	True_Pos
366	PLATE 09	2.459	POS	4.436	POS	True_Pos
368	PLATE 09	2.591	POS	5.026	POS	True_Pos
369	PLATE 09	2.661	POS	5.205	POS	True_Pos
370	PLATE 09	2.234	POS	3.734	POS	True_Pos
372	PLATE 22	1.736	POS	2.486	POS	True_Pos
373	PLATE 09	2.180	POS	3.969	POS	True_Pos
374	PLATE 09	2.148	POS	4.004	POS	True_Pos
375	PLATE 09	1.537	POS	2.295	POS	True_Pos
376	PLATE 22	1.592	POS	2.225	POS	True_Pos
377	PLATE 09	2.591	POS	5.342	POS	True_Pos
378	PLATE 09	1.623	POS	2.496	POS	True_Pos
379	PLATE 09	2.437	POS	4.725	POS	True_Pos
380	PLATE 30	1.694	POS	2.279	POS	True_Pos
381	PLATE 09	2.299	POS	4.666	POS	True_Pos
382	PLATE 09	2.421	POS	4.798	POS	True_Pos
383	PLATE 10	1.734	POS	2.823	POS	True_Pos
384	PLATE 10	2.119	POS	3.513	POS	True_Pos
385	PLATE 32	2.082	POS	3.290	POS	True_Pos
387	PLATE 33	2.458	POS	4.513	POS	True_Pos
388	PLATE 22	1.535	POS	2.068	POS	True_Pos
389	PLATE 33	2.399	POS	3.952	POS	True_Pos
390	PLATE 22	1.483	POS	2.057	POS	True_Pos
391	PLATE 22	1.684	POS	2.495	POS	True_Pos
392	PLATE 10	1.814	POS	2.618	POS	True_Pos
393	PLATE 32	2.326	POS	3.605	POS	True_Pos
394	PLATE 30	2.517	POS	3.716	POS	True_Pos
395	PLATE 10	2.434	POS	4.515	POS	True_Pos
396	PLATE 10	2.329	POS	4.079	POS	True_Pos
397	PLATE 10	2.515	POS	4.311	POS	True_Pos
398	PLATE 10	2.613	POS	4.289	POS	True_Pos
399	PLATE 10	2.065	POS	3.056	POS	True_Pos
400	PLATE 10	1.677	POS	2.244	POS	True_Pos
401	PLATE 10	2.462	POS	4.294	POS	True_Pos
402	PLATE 10	1.472	POS	1.839	POS	True_Pos
403	PLATE 30	1.712	POS	4.184	POS	True_Pos
404	PLATE 22	1.694	POS	3.000	POS	True_Pos
405	PLATE 10	1.946	POS	3.057	POS	True_Pos
407	PLATE 22	1.596	POS	2.466	POS	True_Pos
409	PLATE 22	2.009	POS	3.119	POS	True_Pos
410	PLATE 37	1.968	POS	3.752	POS	True_Pos
411	PLATE 22	1.808	POS	2.379	POS	True_Pos
412	PLATE 22	1.794	POS	2.647	POS	True_Pos
413	PLATE 31	1.916	POS	3.334	POS	True_Pos
414	PLATE 22	1.472	POS	1.923	POS	True_Pos
415	PLATE 22	1.687	POS	2.447	POS	True_Pos

417	PLATE 10	2.453	POS	4.794	POS	True_Pos
418	PLATE 10	2.100	POS	3.634	POS	True_Pos
419	PLATE 10	2.342	POS	3.905	POS	True_Pos
420	PLATE 10	2.498	POS	4.552	POS	True_Pos
421	PLATE 10	2.489	POS	4.452	POS	True_Pos
422	PLATE 23	1.457	POS	2.214	POS	True_Pos
423	PLATE 10	2.738	POS	5.898	POS	True_Pos
424	PLATE 10	1.856	POS	2.769	POS	True_Pos
426	PLATE 11	1.569	POS	2.635	POS	True_Pos
427	PLATE 32	2.228	POS	3.952	POS	True_Pos
428	PLATE 31	2.431	POS	4.607	POS	True_Pos
429	PLATE 28	2.449	POS	5.695	POS	True_Pos
430	PLATE 31	1.846	POS	2.680	POS	True_Pos
431	PLATE 32	2.016	POS	3.392	POS	True_Pos
432	PLATE 28	2.457	POS	5.975	POS	True_Pos
434	PLATE 28	2.457	POS	6.025	POS	True_Pos
435	PLATE 28	2.420	POS	6.177	POS	True_Pos
436	PLATE 27	1.595	POS	2.606	POS	True_Pos
437	PLATE 27	1.722	POS	2.912	POS	True_Pos
438	PLATE 11	2.606	POS	5.828	POS	True_Pos
439	PLATE 11	1.535	POS	2.259	POS	True_Pos
440	PLATE 11	1.822	POS	2.677	POS	True_Pos
441	PLATE 29	2.023	POS	3.611	POS	True_Pos
442	PLATE 11	1.512	POS	1.993	POS	True_Pos
444	PLATE 30	2.138	POS	2.953	POS	True_Pos
445	PLATE 11	1.639	POS	2.546	POS	True_Pos
446	PLATE 11	2.098	POS	4.409	POS	True_Pos
448	PLATE 11	1.528	POS	2.471	POS	True_Pos
449	PLATE 11	1.639	POS	2.458	POS	True_Pos
450	PLATE 11	1.443	POS	1.897	POS	True_Pos
451	PLATE 11	2.378	POS	4.002	POS	True_Pos
452	PLATE 11	2.378	POS	4.097	POS	True_Pos
453	PLATE 11	2.552	POS	5.107	POS	True_Pos
454	PLATE 11	2.354	POS	4.709	POS	True_Pos
455	PLATE 11	2.556	POS	6.438	POS	True_Pos
456	PLATE 11	2.464	POS	7.652	POS	True_Pos
458	PLATE 11	1.788	POS	3.581	POS	True_Pos
459	PLATE 11	2.103	POS	5.080	POS	True_Pos
460	PLATE 11	2.296	POS	6.945	POS	True_Pos
461	PLATE 31	2.282	POS	4.350	POS	True_Pos
462	PLATE 28	2.256	POS	3.987	POS	True_Pos
463	PLATE 28	1.601	POS	2.444	POS	True_Pos
464	PLATE 28	2.521	POS	4.770	POS	True_Pos
467	PLATE 28	2.469	POS	4.217	POS	True_Pos
468	PLATE 28	2.124	POS	3.402	POS	True_Pos
470	PLATE 31	1.913	POS	3.100	POS	True_Pos
473	PLATE 28	1.570	POS	2.213	POS	True_Pos
474	PLATE 28	2.546	POS	4.849	POS	True_Pos
476	PLATE 27	2.027	POS	3.389	POS	True_Pos
478	PLATE 22	1.713	POS	2.990	POS	True_Pos

480	PLATE 12	1.686	POS	2.267	POS	True_Pos
482	PLATE 28	2.606	POS	5.260	POS	True_Pos
483	PLATE 22	1.438	POS	2.010	POS	True_Pos
484	PLATE 12	2.567	POS	4.834	POS	True_Pos
486	PLATE 12	2.561	POS	6.731	POS	True_Pos
487	PLATE 12	2.079	POS	4.492	POS	True_Pos
488	PLATE 28	2.596	POS	5.669	POS	True_Pos
490	PLATE 12	2.443	POS	4.008	POS	True_Pos
491	PLATE 12	2.403	POS	4.095	POS	True_Pos
493	PLATE 32	2.413	POS	4.067	POS	True_Pos
494	PLATE 12	2.620	POS	4.965	POS	True_Pos
495	PLATE 12	2.558	POS	6.281	POS	True_Pos
497	PLATE 12	1.537	POS	2.716	POS	True_Pos
498	PLATE 22	1.521	POS	2.360	POS	True_Pos
499	PLATE 12	2.479	POS	7.925	POS	True_Pos
500	PLATE 12	2.533	POS	8.012	POS	True_Pos
502	PLATE 29	2.485	POS	5.279	POS	True_Pos
503	PLATE 22	2.407	POS	4.456	POS	True_Pos
504	PLATE 29	2.471	POS	4.984	POS	True_Pos
506	PLATE 29	2.300	POS	4.485	POS	True_Pos
507	PLATE 31	2.110	POS	3.311	POS	True_Pos
508	PLATE 29	2.329	POS	4.665	POS	True_Pos
509	PLATE 31	1.636	POS	2.256	POS	True_Pos
511	PLATE 31	2.656	POS	5.093	POS	True_Pos
512	PLATE 34	1.956	POS	3.293	POS	True_Pos
514	PLATE 24	2.019	POS	3.457	POS	True_Pos
515	PLATE 24	1.885	POS	3.200	POS	True_Pos
516	PLATE 13	1.961	POS	2.317	POS	True_Pos
517	PLATE 13	1.888	POS	1.958	POS	True_Pos
518	PLATE 24	2.203	POS	4.318	POS	True_Pos
519	PLATE 13	2.268	POS	2.223	POS	True_Pos
520	PLATE 13	2.177	POS	2.320	POS	True_Pos
521	PLATE 24	1.718	POS	2.882	POS	True_Pos
523	PLATE 24	1.981	POS	3.218	POS	True_Pos
524	PLATE 13	1.908	POS	2.139	POS	True_Pos
526	PLATE 31	1.629	POS	2.464	POS	True_Pos
527	PLATE 13	1.920	POS	3.421	POS	True_Pos
528	PLATE 13	2.039	POS	3.293	POS	True_Pos
529	PLATE 34	1.438	POS	1.939	POS	True_Pos
530	PLATE 34	1.599	POS	2.531	POS	True_Pos
531	PLATE 30	1.582	POS	2.046	POS	True_Pos
532	PLATE 13	1.578	POS	2.275	POS	True_Pos
535	PLATE 13	1.706	POS	2.875	POS	True_Pos
536	PLATE 24	2.165	POS	3.720	POS	True_Pos
537	PLATE 13	1.599	POS	3.138	POS	True_Pos
538	PLATE 13	1.412	POS	2.515	POS	True_Pos
541	PLATE 13	2.081	POS	5.564	POS	True_Pos
542	PLATE 31	2.582	POS	5.272	POS	True_Pos
543	PLATE 29	2.114	POS	3.996	POS	True_Pos
545	PLATE 31	2.129	POS	3.624	POS	True_Pos

546	PLATE 31	2.445	POS	5.177	POS	True_Pos
547	PLATE 23	1.682	POS	2.828	POS	True_Pos
549	PLATE 31	1.547	POS	2.318	POS	True_Pos
550	PLATE 29	2.481	POS	7.847	POS	True_Pos
551	PLATE 31	2.348	POS	4.579	POS	True_Pos
552	PLATE 24	1.915	POS	3.171	POS	True_Pos
553	PLATE 14	2.140	POS	6.620	POS	True_Pos
555	PLATE 34	2.000	POS	3.224	POS	True_Pos
556	PLATE 14	2.198	POS	5.107	POS	True_Pos
561	PLATE 24	2.302	POS	3.658	POS	True_Pos
562	PLATE 14	2.175	POS	5.262	POS	True_Pos
564	PLATE 14	1.525	POS	3.584	POS	True_Pos
565	PLATE 24	1.741	POS	2.483	POS	True_Pos
566	PLATE 14	1.652	POS	3.866	POS	True_Pos
570	PLATE 14	2.370	POS	5.709	POS	True_Pos
571	PLATE 24	1.949	POS	3.134	POS	True_Pos
572	PLATE 14	2.210	POS	4.804	POS	True_Pos
573	PLATE 24	1.591	POS	2.380	POS	True_Pos
575	PLATE 14	2.093	POS	4.423	POS	True_Pos
577	PLATE 31	2.398	POS	4.159	POS	True_Pos
578	PLATE 24	1.739	POS	2.956	POS	True_Pos
580	PLATE 24	1.647	POS	2.419	POS	True_Pos
584	PLATE 34	1.958	POS	3.214	POS	True_Pos
585	PLATE 14	1.996	POS	6.995	POS	True_Pos
586	PLATE 31	2.510	POS	4.923	POS	True_Pos
587	PLATE 31	2.472	POS	4.795	POS	True_Pos
589	PLATE 27	2.580	POS	5.131	POS	True_Pos
590	PLATE 34	1.581	POS	2.238	POS	True_Pos
592	PLATE 30	1.948	POS	2.376	POS	True_Pos
593	PLATE 27	2.508	POS	4.893	POS	True_Pos
594	PLATE 34	1.614	POS	2.343	POS	True_Pos
595	PLATE 29	2.528	POS	7.689	POS	True_Pos
596	PLATE 15	2.064	POS	4.865	POS	True_Pos
597	PLATE 15	2.157	POS	4.401	POS	True_Pos
599	PLATE 15	1.721	POS	2.603	POS	True_Pos
600	PLATE 24	1.822	POS	1.863	POS	True_Pos
601	PLATE 15	1.995	POS	3.363	POS	True_Pos
603	PLATE 15	2.158	POS	3.883	POS	True_Pos
605	PLATE 30	1.667	POS	2.935	POS	True_Pos
607	PLATE 15	1.953	POS	3.857	POS	True_Pos
608	PLATE 15	2.451	POS	5.479	POS	True_Pos
609	PLATE 15	2.726	POS	5.789	POS	True_Pos
611	PLATE 15	2.624	POS	6.431	POS	True_Pos
612	PLATE 30	2.219	POS	3.169	POS	True_Pos
613	PLATE 15	1.982	POS	3.492	POS	True_Pos
616	PLATE 15	2.205	POS	6.429	POS	True_Pos
617	PLATE 15	1.464	POS	2.668	POS	True_Pos
618	PLATE 15	2.296	POS	7.251	POS	True_Pos
620	PLATE 15	2.475	POS	9.499	POS	True_Pos
621	PLATE 31	1.488	POS	2.032	POS	True_Pos

622	PLATE 30	2.446	POS	4.320	POS	True_Pos
623	PLATE 34	1.506	POS	2.019	POS	True_Pos
624	PLATE 25	1.548	POS	1.762	POS	True_Pos
625	PLATE 27	2.456	POS	4.751	POS	True_Pos
627	PLATE 31	2.300	POS	4.163	POS	True_Pos
628	PLATE 31	1.991	POS	3.226	POS	True_Pos
629	PLATE 16	1.562	POS	1.920	POS	True_Pos
631	PLATE 31	2.090	POS	3.825	POS	True_Pos
633	PLATE 32	1.705	POS	2.446	POS	True_Pos
636	PLATE 16	1.892	POS	3.253	POS	True_Pos
637	PLATE 16	2.176	POS	3.974	POS	True_Pos
638	PLATE 25	1.455	POS	1.828	POS	True_Pos
639	PLATE 16	2.695	POS	4.841	POS	True_Pos
640	PLATE 25	1.710	POS	1.784	POS	True_Pos
641	PLATE 34	2.239	POS	3.993	POS	True_Pos
643	PLATE 16	2.484	POS	4.670	POS	True_Pos
644	PLATE 16	1.742	POS	2.698	POS	True_Pos
646	PLATE 16	1.532	POS	2.480	POS	True_Pos
647	PLATE 25	1.504	POS	0.492	NEG	False_Neg
648	PLATE 16	1.431	POS	2.368	POS	True_Pos
651	PLATE 16	1.932	POS	3.066	POS	True_Pos
652	PLATE 30	1.453	POS	4.222	POS	True_Pos
653	PLATE 16	1.488	POS	2.060	POS	True_Pos
655	PLATE 16	2.160	POS	3.734	POS	True_Pos
656	PLATE 25	1.547	POS	2.795	POS	True_Pos
657	PLATE 16	1.559	POS	2.710	POS	True_Pos
658	PLATE 25	1.546	POS	1.735	POS	True_Pos
661	PLATE 32	2.416	POS	4.148	POS	True_Pos
666	PLATE 30	2.041	POS	3.657	POS	True_Pos
667	PLATE 33	2.498	POS	5.218	POS	True_Pos
668	PLATE 30	2.033	POS	3.755	POS	True_Pos
669	PLATE 25	1.572	POS	1.499	NEG	False_Neg
670	PLATE 33	2.315	POS	4.450	POS	True_Pos
671	PLATE 17	1.522	POS	1.828	POS	True_Pos
672	PLATE 33	2.269	POS	4.452	POS	True_Pos
673	PLATE 34	2.670	POS	5.027	POS	True_Pos
674	PLATE 33	2.377	POS	4.694	POS	True_Pos
675	PLATE 30	1.688	POS	3.543	POS	True_Pos
676	PLATE 17	2.314	POS	4.359	POS	True_Pos
677	PLATE 33	2.442	POS	5.200	POS	True_Pos
678	PLATE 32	2.628	POS	5.007	POS	True_Pos
682	PLATE 17	2.483	POS	4.088	POS	True_Pos
684	PLATE 17	2.417	POS	3.714	POS	True_Pos
685	PLATE 34	1.916	POS	2.773	POS	True_Pos
687	PLATE 17	2.368	POS	3.699	POS	True_Pos
688	PLATE 30	1.819	POS	3.310	POS	True_Pos
689	PLATE 17	2.338	POS	3.854	POS	True_Pos
690	PLATE 17	2.293	POS	4.193	POS	True_Pos
691	PLATE 17	2.170	POS	4.031	POS	True_Pos
692	PLATE 25	1.455	POS	1.868	POS	True_Pos

693	PLATE 32	2.498	POS	4.529	POS	True_Pos
694	PLATE 17	1.897	POS	2.631	POS	True_Pos
695	PLATE 17	1.619	POS	2.044	POS	True_Pos
696	PLATE 17	2.365	POS	3.529	POS	True_Pos
697	PLATE 17	1.542	POS	1.802	POS	True_Pos
698	PLATE 25	1.482	POS	1.365	NEG	False_Neg
699	PLATE 17	2.268	POS	3.918	POS	True_Pos
700	PLATE 25	1.537	POS	2.078	POS	True_Pos
701	PLATE 25	1.598	POS	1.595	NEG	False_Neg
702	PLATE 17	2.037	POS	3.587	POS	True_Pos
703	PLATE 17	2.148	POS	3.881	POS	True_Pos
704	PLATE 17	2.172	POS	3.978	POS	True_Pos
705	PLATE 17	1.655	POS	2.558	POS	True_Pos
706	PLATE 18	1.648	POS	2.504	POS	True_Pos
707	PLATE 32	2.403	POS	4.517	POS	True_Pos
708	PLATE 32	2.423	POS	4.446	POS	True_Pos
709	PLATE 18	1.615	POS	2.003	POS	True_Pos
710	PLATE 18	1.712	POS	2.054	POS	True_Pos
711	PLATE 32	2.031	POS	3.121	POS	True_Pos
712	PLATE 25	1.585	POS	1.431	NEG	False_Neg
713	PLATE 32	2.157	POS	3.377	POS	True_Pos
714	PLATE 32	2.637	POS	4.820	POS	True_Pos
715	PLATE 32	2.324	POS	3.934	POS	True_Pos
716	PLATE 18	2.410	POS	4.868	POS	True_Pos
717	PLATE 25	1.698	POS	1.025	NEG	False_Neg
718	PLATE 30	1.875	POS	2.953	POS	True_Pos
719	PLATE 25	1.720	POS	1.146	NEG	False_Neg
720	PLATE 18	2.639	POS	4.261	POS	True_Pos
721	PLATE 18	2.126	POS	3.016	POS	True_Pos
722	PLATE 18	1.804	POS	2.396	POS	True_Pos
723	PLATE 18	2.184	POS	3.272	POS	True_Pos
724	PLATE 18	2.344	POS	3.844	POS	True_Pos
725	PLATE 18	1.991	POS	3.261	POS	True_Pos
726	PLATE 18	2.529	POS	5.245	POS	True_Pos
727	PLATE 18	2.714	POS	5.333	POS	True_Pos
728	PLATE 18	2.684	POS	4.963	POS	True_Pos
729	PLATE 18	2.596	POS	4.238	POS	True_Pos
730	PLATE 18	2.358	POS	3.592	POS	True_Pos
731	PLATE 18	2.652	POS	4.362	POS	True_Pos
732	PLATE 18	2.385	POS	3.724	POS	True_Pos
734	PLATE 31	1.731	POS	2.480	POS	True_Pos
735	PLATE 18	2.482	POS	4.753	POS	True_Pos
737	PLATE 18	2.153	POS	3.965	POS	True_Pos
738	PLATE 18	2.136	POS	3.841	POS	True_Pos
740	PLATE 26	2.118	POS	1.747	POS	True_Pos
741	PLATE 18	1.911	POS	3.330	POS	True_Pos
742	PLATE 19	1.723	POS	2.633	POS	True_Pos
744	PLATE 31	2.185	POS	3.348	POS	True_Pos
745	PLATE 26	1.859	POS	1.356	NEG	False_Neg
746	PLATE 26	1.632	POS	1.455	NEG	False_Neg

747	PLATE 30	2.136	POS	3.770	POS	True_Pos
748	PLATE 31	1.838	POS	2.671	POS	True_Pos
749	PLATE 19	1.556	POS	2.025	POS	True_Pos
750	PLATE 32	2.133	POS	3.564	POS	True_Pos
751	PLATE 28	2.487	POS	4.604	POS	True_Pos
752	PLATE 19	2.308	POS	4.050	POS	True_Pos
753	PLATE 19	2.250	POS	3.766	POS	True_Pos
754	PLATE 19	2.412	POS	4.112	POS	True_Pos
755	PLATE 19	1.618	POS	2.240	POS	True_Pos
758	PLATE 19	2.280	POS	3.532	POS	True_Pos
759	PLATE 19	1.974	POS	2.855	POS	True_Pos
761	PLATE 19	1.731	POS	2.533	POS	True_Pos
763	PLATE 26	2.153	POS	1.727	POS	True_Pos
764	PLATE 19	2.465	POS	4.146	POS	True_Pos
765	PLATE 19	1.624	POS	2.092	POS	True_Pos
766	PLATE 19	2.185	POS	3.330	POS	True_Pos
767	PLATE 19	2.352	POS	3.741	POS	True_Pos
769	PLATE 19	1.637	POS	2.067	POS	True_Pos
771	PLATE 19	2.573	POS	4.535	POS	True_Pos
772	PLATE 19	1.538	POS	2.339	POS	True_Pos
773	PLATE 19	2.327	POS	4.147	POS	True_Pos
774	PLATE 19	2.073	POS	3.346	POS	True_Pos
775	PLATE 26	1.691	POS	0.840	NEG	False_Neg
776	PLATE 37	1.454	POS	2.419	POS	True_Pos
777	PLATE 26	1.937	POS	1.763	POS	True_Pos
778	PLATE 19	2.620	POS	4.836	POS	True_Pos
779	PLATE 37	1.480	POS	2.557	POS	True_Pos
780	PLATE 26	2.008	POS	2.032	POS	True_Pos
782	PLATE 20	1.586	POS	2.531	POS	True_Pos
783	PLATE 32	2.108	POS	3.268	POS	True_Pos
784	PLATE 32	2.259	POS	4.076	POS	True_Pos
785	PLATE 20	1.711	POS	2.525	POS	True_Pos
786	PLATE 26	1.456	POS	1.951	POS	True_Pos
788	PLATE 32	2.298	POS	3.867	POS	True_Pos
789	PLATE 20	1.524	POS	2.136	POS	True_Pos
790	PLATE 26	1.835	POS	1.359	NEG	False_Neg
791	PLATE 32	2.620	POS	5.037	POS	True_Pos
792	PLATE 26	1.716	POS	1.708	POS	True_Pos
794	PLATE 20	1.658	POS	2.721	POS	True_Pos
795	PLATE 20	2.103	POS	3.883	POS	True_Pos
796	PLATE 20	1.695	POS	2.541	POS	True_Pos
797	PLATE 20	2.397	POS	4.320	POS	True_Pos
798	PLATE 20	1.773	POS	2.750	POS	True_Pos
799	PLATE 20	2.083	POS	3.598	POS	True_Pos
800	PLATE 20	1.627	POS	2.486	POS	True_Pos
801	PLATE 20	2.209	POS	4.129	POS	True_Pos
802	PLATE 30	2.210	POS	3.854	POS	True_Pos
803	PLATE 20	1.934	POS	3.400	POS	True_Pos
804	PLATE 20	2.319	POS	4.409	POS	True_Pos
805	PLATE 20	1.585	POS	2.487	POS	True_Pos

806	PLATE 20	2.558	POS	4.827	POS	True_Pos
807	PLATE 26	1.512	POS	1.837	POS	True_Pos
808	PLATE 20	2.028	POS	3.517	POS	True_Pos
809	PLATE 37	1.539	POS	2.667	POS	True_Pos
810	PLATE 30	2.087	POS	2.937	POS	True_Pos
812	PLATE 30	2.319	POS	3.469	POS	True_Pos
813	PLATE 26	1.568	POS	2.151	POS	True_Pos
814	PLATE 37	2.013	POS	4.009	POS	True_Pos
815	PLATE 30	2.331	POS	4.143	POS	True_Pos
816	PLATE 20	2.161	POS	4.199	POS	True_Pos
818	PLATE 20	2.267	POS	4.495	POS	True_Pos
819	PLATE 30	2.194	POS	3.689	POS	True_Pos
821	PLATE 20	2.610	POS	5.454	POS	True_Pos
822	PLATE 33	2.222	POS	3.789	POS	True_Pos
823	PLATE 27	1.709	POS	2.961	POS	True_Pos
824	PLATE 03A	2.043	POS	3.254	POS	True_Pos
825	PLATE 32	1.985	POS	2.864	POS	True_Pos
826	PLATE 27	1.853	POS	3.456	POS	True_Pos
827	PLATE 32	2.228	POS	3.820	POS	True_Pos
828	PLATE 03A	1.516	POS	1.845	POS	True_Pos
829	PLATE 03A	1.529	POS	1.857	POS	True_Pos
830	PLATE 03A	2.591	POS	4.632	POS	True_Pos
831	PLATE 27	2.001	POS	3.658	POS	True_Pos
832	PLATE 27	1.533	POS	2.567	POS	True_Pos
833	PLATE 30	1.930	POS	3.126	POS	True_Pos
834	PLATE 32	1.785	POS	2.686	POS	True_Pos
836	PLATE 35	1.559	POS	2.317	POS	True_Pos
837	PLATE 35	2.171	POS	3.845	POS	True_Pos
838	PLATE 35	2.288	POS	3.980	POS	True_Pos
839	PLATE 35	2.534	POS	4.991	POS	True_Pos
840	PLATE 37	2.342	POS	4.939	POS	True_Pos
841	PLATE 35	1.713	POS	2.587	POS	True_Pos
842	PLATE 35	2.161	POS	3.666	POS	True_Pos
843	PLATE 35	2.188	POS	3.726	POS	True_Pos
844	PLATE 35	2.389	POS	4.117	POS	True_Pos
845	PLATE 35	1.514	POS	1.898	POS	True_Pos
846	PLATE 35	2.637	POS	4.555	POS	True_Pos
847	PLATE 35	2.656	POS	4.774	POS	True_Pos
848	PLATE 35	2.300	POS	3.827	POS	True_Pos
849	PLATE 35	2.497	POS	4.503	POS	True_Pos
850	PLATE 35	1.913	POS	3.141	POS	True_Pos
851	PLATE 37	1.959	POS	3.558	POS	True_Pos
853	PLATE 35	1.933	POS	3.041	POS	True_Pos
854	PLATE 38	2.169	POS	3.358	POS	True_Pos
855	PLATE 35	2.731	POS	4.893	POS	True_Pos
856	PLATE 35	2.726	POS	4.925	POS	True_Pos
857	PLATE 35	1.558	POS	1.984	POS	True_Pos
858	PLATE 35	2.665	POS	4.788	POS	True_Pos
859	PLATE 35	2.576	POS	5.342	POS	True_Pos
860	PLATE 35	1.674	POS	2.361	POS	True_Pos

861	PLATE 35	2.447	POS	4.664	POS	True_Pos
862	PLATE 35	2.616	POS	5.440	POS	True_Pos
863	PLATE 35	2.700	POS	5.444	POS	True_Pos
