# Supplementary material for: Validation of new equipment for SARS-CoV-2 diagnosis in Ecuador: Detection of the virus and antibodies generated by disease and vaccines with one POC device
Source: PLoS One. 2025 Apr 16;20(4):e0321794. doi: 10.1371/journal.pone.0321794 (PMC12002511; doi:10.1371/journal.pone.0321794)
Supplement: S5 File — (PDF) [file pone.0321794.s005.pdf]

SAMPLE	PLATE	Spectrophotometer (ABS relative ratio)	SPEC Nucleocapsid	PLUM (PRU relative ratio)	PLUM Nucleocapsid	VALIDATION
1	PLATE 01	0.156	NEG	0.204	NEG	True_Neg
4	PLATE 01	0.800	NEG	0.909	POS	False_Pos
7	PLATE 01	0.344	NEG	0.366	NEG	True_Neg
8	PLATE 01	0.776	NEG	0.953	POS	False_Pos
9	PLATE 01	0.255	NEG	0.260	NEG	True_Neg
14	PLATE 01	0.311	NEG	0.279	NEG	True_Neg
19	PLATE 01	0.559	NEG	0.568	NEG	True_Neg
20	PLATE 01	0.318	NEG	0.293	NEG	True_Neg
21	PLATE 01	0.678	NEG	0.625	NEG	True_Neg
26	PLATE 01	0.765	NEG	0.739	NEG	True_Neg
28	PLATE 01	0.613	NEG	0.651	NEG	True_Neg
37	PLATE 01	0.728	NEG	0.737	NEG	True_Neg
41	PLATE 02	0.555	NEG	0.599	NEG	True_Neg
42	PLATE 02	0.783	NEG	0.896	POS	False_Pos
43	PLATE 02	0.506	NEG	0.538	NEG	True_Neg
44	PLATE 02	0.374	NEG	0.425	NEG	True_Neg
50	PLATE 02	0.471	NEG	0.393	NEG	True_Neg
51	PLATE 02	0.523	NEG	0.456	NEG	True_Neg
57	PLATE 02	0.736	NEG	0.713	NEG	True_Neg
58	PLATE 02	0.781	NEG	0.713	NEG	True_Neg
60	PLATE 02	0.767	NEG	0.743	NEG	True_Neg
62	PLATE 02	0.603	NEG	0.517	NEG	True_Neg
64	PLATE 02	0.518	NEG	0.499	NEG	True_Neg
66	PLATE 02	0.480	NEG	0.492	NEG	True_Neg
68	PLATE 02	0.700	NEG	0.668	NEG	True_Neg
69	PLATE 02	0.624	NEG	0.593	NEG	True_Neg
71	PLATE 02	0.460	NEG	0.412	NEG	True_Neg
72	PLATE 02	0.625	NEG	0.576	NEG	True_Neg
73	PLATE 02	0.410	NEG	0.344	NEG	True_Neg
74	PLATE 02	0.500	NEG	0.497	NEG	True_Neg
76	PLATE 03	0.593	NEG	0.695	NEG	True_Neg
80	PLATE 29	0.563	NEG	0.467	NEG	True_Neg
81	PLATE 03	0.337	NEG	0.356	NEG	True_Neg
82	PLATE 03	0.493	NEG	0.527	NEG	True_Neg
83	PLATE 03	0.506	NEG	0.513	NEG	True_Neg
89	PLATE 03	0.632	NEG	0.615	NEG	True_Neg
90	PLATE 03	0.385	NEG	0.332	NEG	True_Neg
91	PLATE 03	0.571	NEG	0.652	NEG	True_Neg
92	PLATE 27	0.326	NEG	0.379	NEG	True_Neg
94	PLATE 27	0.154	NEG	0.225	NEG	True_Neg
95	PLATE 03	0.684	NEG	0.603	NEG	True_Neg
96	PLATE 03	0.674	NEG	0.605	NEG	True_Neg
97	PLATE 03	0.623	NEG	0.565	NEG	True_Neg
98	PLATE 03	0.583	NEG	0.504	NEG	True_Neg
99	PLATE 03	0.782	NEG	0.753	NEG	True_Neg
100	PLATE 03	0.573	NEG	0.544	NEG	True_Neg
101	PLATE 03	0.479	NEG	0.502	NEG	True_Neg

102	PLATE 27	0.374	NEG	0.311	NEG	True_Neg
103	PLATE 03	0.554	NEG	0.585	NEG	True_Neg
104	PLATE 27	0.247	NEG	0.321	NEG	True_Neg
105	PLATE 03	0.450	NEG	0.343	NEG	True_Neg
106	PLATE 03	0.454	NEG	0.394	NEG	True_Neg
107	PLATE 27	0.192	NEG	0.220	NEG	True_Neg
108	PLATE 03	0.378	NEG	0.322	NEG	True_Neg
109	PLATE 27	0.460	NEG	0.557	NEG	True_Neg
110	PLATE 27	0.410	NEG	0.466	NEG	True_Neg
111	PLATE 27	0.316	NEG	0.356	NEG	True_Neg
112	PLATE 04	0.282	NEG	0.298	NEG	True_Neg
113	PLATE 04	0.369	NEG	0.360	NEG	True_Neg
114	PLATE 04	0.489	NEG	0.516	NEG	True_Neg
115	PLATE 04	0.533	NEG	0.592	NEG	True_Neg
116	PLATE 04	0.438	NEG	0.520	NEG	True_Neg
117	PLATE 04	0.394	NEG	0.455	NEG	True_Neg
118	PLATE 04	0.682	NEG	0.746	NEG	True_Neg
119	PLATE 04	0.493	NEG	0.429	NEG	True_Neg
121	PLATE 04	0.716	NEG	0.640	NEG	True_Neg
124	PLATE 04	0.697	NEG	0.639	NEG	True_Neg
125	PLATE 04	0.630	NEG	0.617	NEG	True_Neg
127	PLATE 04	0.357	NEG	0.374	NEG	True_Neg
128	PLATE 04	0.342	NEG	0.337	NEG	True_Neg
133	PLATE 04	0.642	NEG	0.561	NEG	True_Neg
137	PLATE 04	0.462	NEG	0.397	NEG	True_Neg
138	PLATE 27	0.287	NEG	0.301	NEG	True_Neg
139	PLATE 04	0.646	NEG	0.706	NEG	True_Neg
140	PLATE 27	0.727	NEG	0.730	NEG	True_Neg
142	PLATE 04	0.438	NEG	0.343	NEG	True_Neg
143	PLATE 04	0.758	NEG	0.733	NEG	True_Neg
146	PLATE 04	0.640	NEG	0.588	NEG	True_Neg
147	PLATE 04	0.680	NEG	0.649	NEG	True_Neg
148	PLATE 27	0.484	NEG	0.473	NEG	True_Neg
149	PLATE 04	0.149	NEG	0.106	NEG	True_Neg
151	PLATE 05	0.315	NEG	0.343	NEG	True_Neg
152	PLATE 05	0.419	NEG	0.498	NEG	True_Neg
155	PLATE 05	0.473	NEG	0.606	NEG	True_Neg
160	PLATE 05	0.497	NEG	0.500	NEG	True_Neg
161	PLATE 05	0.426	NEG	0.359	NEG	True_Neg
162	PLATE 05	0.283	NEG	0.236	NEG	True_Neg
163	PLATE 05	0.643	NEG	0.633	NEG	True_Neg
164	PLATE 05	0.510	NEG	0.472	NEG	True_Neg
165	PLATE 05	0.304	NEG	0.259	NEG	True_Neg
166	PLATE 05	0.549	NEG	0.559	NEG	True_Neg
168	PLATE 05	0.663	NEG	0.722	NEG	True_Neg
170	PLATE 05	0.478	NEG	0.508	NEG	True_Neg
172	PLATE 05	0.623	NEG	0.581	NEG	True_Neg
173	PLATE 05	0.591	NEG	0.562	NEG	True_Neg
174	PLATE 05	0.571	NEG	0.553	NEG	True_Neg
175	PLATE 05	0.768	NEG	0.713	NEG	True_Neg

177	PLATE 05	0.539	NEG	0.517	NEG	True_Neg
179	PLATE 05	0.403	NEG	0.512	NEG	True_Neg
180	PLATE 05	0.717	NEG	0.899	POS	False_Pos
183	PLATE 05	0.802	NEG	0.833	POS	False_Pos
185	PLATE 05	0.567	NEG	0.578	NEG	True_Neg
186	PLATE 05	0.716	NEG	0.741	NEG	True_Neg
187	PLATE 05	0.560	NEG	0.517	NEG	True_Neg
188	PLATE 05	0.411	NEG	0.373	NEG	True_Neg
189	PLATE 05	0.591	NEG	0.593	NEG	True_Neg
190	PLATE 05	0.745	NEG	0.901	POS	False_Pos
191	PLATE 06	0.199	NEG	0.214	NEG	True_Neg
194	PLATE 06	0.329	NEG	0.366	NEG	True_Neg
196	PLATE 06	0.220	NEG	0.210	NEG	True_Neg
198	PLATE 06	0.513	NEG	0.448	NEG	True_Neg
199	PLATE 06	0.363	NEG	0.260	NEG	True_Neg
200	PLATE 06	0.564	NEG	0.507	NEG	True_Neg
201	PLATE 06	0.373	NEG	0.294	NEG	True_Neg
203	PLATE 06	0.410	NEG	0.326	NEG	True_Neg
204	PLATE 06	0.376	NEG	0.334	NEG	True_Neg
206	PLATE 06	0.184	NEG	0.123	NEG	True_Neg
207	PLATE 06	0.504	NEG	0.441	NEG	True_Neg
208	PLATE 06	0.375	NEG	0.263	NEG	True_Neg
209	PLATE 06	0.298	NEG	0.176	NEG	True_Neg
211	PLATE 06	0.346	NEG	0.249	NEG	True_Neg
212	PLATE 06	0.214	NEG	0.097	NEG	True_Neg
214	PLATE 06	0.415	NEG	0.351	NEG	True_Neg
215	PLATE 06	0.660	NEG	0.718	NEG	True_Neg
217	PLATE 06	0.518	NEG	0.501	NEG	True_Neg
218	PLATE 06	0.673	NEG	0.657	NEG	True_Neg
221	PLATE 06	0.309	NEG	0.243	NEG	True_Neg
224	PLATE 06	0.384	NEG	0.310	NEG	True_Neg
225	PLATE 06	0.591	NEG	0.574	NEG	True_Neg
226	PLATE 06	0.294	NEG	0.255	NEG	True_Neg
228	PLATE 07	0.763	NEG	0.722	NEG	True_Neg
229	PLATE 07	0.173	NEG	0.190	NEG	True_Neg
230	PLATE 07	0.477	NEG	0.519	NEG	True_Neg
231	PLATE 07	0.652	NEG	0.751	NEG	True_Neg
232	PLATE 07	0.136	NEG	0.139	NEG	True_Neg
233	PLATE 07	0.306	NEG	0.298	NEG	True_Neg
234	PLATE 07	0.453	NEG	0.436	NEG	True_Neg
235	PLATE 07	0.501	NEG	0.452	NEG	True_Neg
236	PLATE 07	0.498	NEG	0.475	NEG	True_Neg
237	PLATE 07	0.385	NEG	0.335	NEG	True_Neg
238	PLATE 07	0.370	NEG	0.307	NEG	True_Neg
239	PLATE 27	0.232	NEG	0.237	NEG	True_Neg
240	PLATE 07	0.284	NEG	0.250	NEG	True_Neg
241	PLATE 07	0.233	NEG	0.240	NEG	True_Neg
242	PLATE 07	0.604	NEG	0.647	NEG	True_Neg
243	PLATE 07	0.340	NEG	0.335	NEG	True_Neg
244	PLATE 07	0.354	NEG	0.325	NEG	True_Neg

245	PLATE 07	0.413	NEG	0.351	NEG	True_Neg
247	PLATE 07	0.650	NEG	0.597	NEG	True_Neg
248	PLATE 07	0.337	NEG	0.286	NEG	True_Neg
250	PLATE 07	0.680	NEG	0.596	NEG	True_Neg
251	PLATE 07	0.402	NEG	0.321	NEG	True_Neg
252	PLATE 07	0.470	NEG	0.448	NEG	True_Neg
254	PLATE 07	0.156	NEG	0.130	NEG	True_Neg
255	PLATE 07	0.294	NEG	0.321	NEG	True_Neg
258	PLATE 07	0.768	NEG	0.772	NEG	True_Neg
259	PLATE 07	0.433	NEG	0.400	NEG	True_Neg
260	PLATE 07	0.549	NEG	0.508	NEG	True_Neg
264	PLATE 07	0.188	NEG	0.156	NEG	True_Neg
265	PLATE 07	0.512	NEG	0.546	NEG	True_Neg
272	PLATE 08	0.444	NEG	0.502	NEG	True_Neg
276	PLATE 08	0.405	NEG	0.365	NEG	True_Neg
278	PLATE 08	0.667	NEG	0.622	NEG	True_Neg
287	PLATE 08	0.677	NEG	0.663	NEG	True_Neg
288	PLATE 08	0.591	NEG	0.534	NEG	True_Neg
289	PLATE 08	0.799	NEG	0.767	NEG	True_Neg
290	PLATE 08	0.697	NEG	0.675	NEG	True_Neg
291	PLATE 08	0.317	NEG	0.290	NEG	True_Neg
293	PLATE 08	0.324	NEG	0.339	NEG	True_Neg
297	PLATE 08	0.549	NEG	0.480	NEG	True_Neg
298	PLATE 08	0.448	NEG	0.383	NEG	True_Neg
303	PLATE 09	0.353	NEG	0.376	NEG	True_Neg
304	PLATE 09	0.603	NEG	0.704	NEG	True_Neg
305	PLATE 09	0.417	NEG	0.444	NEG	True_Neg
307	PLATE 09	0.671	NEG	0.740	NEG	True_Neg
312	PLATE 09	0.562	NEG	0.519	NEG	True_Neg
313	PLATE 09	0.682	NEG	0.670	NEG	True_Neg
317	PLATE 09	0.645	NEG	0.777	NEG	True_Neg
320	PLATE 09	0.527	NEG	0.543	NEG	True_Neg
321	PLATE 09	0.381	NEG	0.305	NEG	True_Neg
322	PLATE 09	0.752	NEG	0.685	NEG	True_Neg
324	PLATE 09	0.636	NEG	0.598	NEG	True_Neg
325	PLATE 09	0.432	NEG	0.348	NEG	True_Neg
326	PLATE 09	0.464	NEG	0.393	NEG	True_Neg
328	PLATE 09	0.251	NEG	0.188	NEG	True_Neg
330	PLATE 09	0.513	NEG	0.490	NEG	True_Neg
331	PLATE 09	0.519	NEG	0.558	NEG	True_Neg
333	PLATE 09	0.512	NEG	0.453	NEG	True_Neg
334	PLATE 09	0.446	NEG	0.409	NEG	True_Neg
335	PLATE 09	0.682	NEG	0.669	NEG	True_Neg
336	PLATE 09	0.650	NEG	0.627	NEG	True_Neg
337	PLATE 27	0.374	NEG	0.444	NEG	True_Neg
340	PLATE 09	0.717	NEG	0.741	NEG	True_Neg
341	PLATE 09	0.438	NEG	0.393	NEG	True_Neg
343	PLATE 10	0.126	NEG	0.209	NEG	True_Neg
353	PLATE 10	0.462	NEG	0.566	NEG	True_Neg
354	PLATE 10	0.698	NEG	0.872	POS	False_Pos

356	PLATE 10	0.332	NEG	0.358	NEG	True_Neg
357	PLATE 27	0.226	NEG	0.281	NEG	True_Neg
358	PLATE 10	0.286	NEG	0.291	NEG	True_Neg
359	PLATE 10	0.115	NEG	0.167	NEG	True_Neg
360	PLATE 10	0.299	NEG	0.314	NEG	True_Neg
362	PLATE 10	0.488	NEG	0.483	NEG	True_Neg
363	PLATE 10	0.412	NEG	0.399	NEG	True_Neg
364	PLATE 10	0.221	NEG	0.225	NEG	True_Neg
365	PLATE 27	0.783	NEG	0.766	NEG	True_Neg
366	PLATE 10	0.726	NEG	0.904	POS	False_Pos
367	PLATE 10	0.307	NEG	0.319	NEG	True_Neg
368	PLATE 27	0.561	NEG	0.541	NEG	True_Neg
369	PLATE 27	0.709	NEG	0.703	NEG	True_Neg
371	PLATE 10	0.349	NEG	0.335	NEG	True_Neg
373	PLATE 10	0.467	NEG	0.475	NEG	True_Neg
377	PLATE 27	0.632	NEG	0.627	NEG	True_Neg
378	PLATE 27	0.484	NEG	0.442	NEG	True_Neg
379	PLATE 10	0.341	NEG	0.432	NEG	True_Neg
380	PLATE 10	0.301	NEG	0.338	NEG	True_Neg
383	PLATE 10	0.655	NEG	0.710	NEG	True_Neg
384	PLATE 10	0.427	NEG	0.429	NEG	True_Neg
385	PLATE 10	0.628	NEG	0.616	NEG	True_Neg
388	PLATE 10	0.512	NEG	0.510	NEG	True_Neg
389	PLATE 10	0.147	NEG	0.148	NEG	True_Neg
393	PLATE 11	0.134	NEG	0.167	NEG	True_Neg
394	PLATE 11	0.182	NEG	0.236	NEG	True_Neg
395	PLATE 11	0.481	NEG	0.527	NEG	True_Neg
396	PLATE 11	0.708	NEG	0.740	NEG	True_Neg
397	PLATE 11	0.636	NEG	0.619	NEG	True_Neg
398	PLATE 11	0.293	NEG	0.263	NEG	True_Neg
399	PLATE 11	0.505	NEG	0.482	NEG	True_Neg
400	PLATE 11	0.203	NEG	0.191	NEG	True_Neg
401	PLATE 11	0.239	NEG	0.217	NEG	True_Neg
402	PLATE 11	0.312	NEG	0.264	NEG	True_Neg
403	PLATE 11	0.455	NEG	0.417	NEG	True_Neg
404	PLATE 11	0.663	NEG	0.707	NEG	True_Neg
405	PLATE 11	0.589	NEG	0.645	NEG	True_Neg
407	PLATE 11	0.713	NEG	0.740	NEG	True_Neg
408	PLATE 11	0.469	NEG	0.417	NEG	True_Neg
409	PLATE 11	0.491	NEG	0.422	NEG	True_Neg
410	PLATE 11	0.335	NEG	0.296	NEG	True_Neg
411	PLATE 11	0.232	NEG	0.216	NEG	True_Neg
412	PLATE 11	0.712	NEG	0.653	NEG	True_Neg
414	PLATE 11	0.381	NEG	0.310	NEG	True_Neg
417	PLATE 11	0.402	NEG	0.422	NEG	True_Neg
418	PLATE 11	0.367	NEG	0.377	NEG	True_Neg
419	PLATE 27	0.439	NEG	0.390	NEG	True_Neg
420	PLATE 11	0.466	NEG	0.442	NEG	True_Neg
421	PLATE 11	0.752	NEG	0.718	NEG	True_Neg
422	PLATE 11	0.417	NEG	0.388	NEG	True_Neg

423	PLATE 11	0.397	NEG	0.360	NEG	True_Neg
424	PLATE 11	0.496	NEG	0.446	NEG	True_Neg
425	PLATE 11	0.287	NEG	0.248	NEG	True_Neg
426	PLATE 11	0.410	NEG	0.371	NEG	True_Neg
427	PLATE 11	0.348	NEG	0.344	NEG	True_Neg
428	PLATE 12	0.313	NEG	0.436	NEG	True_Neg
430	PLATE 12	0.293	NEG	0.394	NEG	True_Neg
431	PLATE 12	0.228	NEG	0.309	NEG	True_Neg
432	PLATE 12	0.787	NEG	1.008	POS	False_Pos
435	PLATE 27	0.315	NEG	0.293	NEG	True_Neg
436	PLATE 12	0.139	NEG	0.233	NEG	True_Neg
437	PLATE 12	0.329	NEG	0.387	NEG	True_Neg
439	PLATE 12	0.497	NEG	0.492	NEG	True_Neg
442	PLATE 12	0.385	NEG	0.391	NEG	True_Neg
443	PLATE 12	0.302	NEG	0.299	NEG	True_Neg
445	PLATE 12	0.596	NEG	0.702	NEG	True_Neg
446	PLATE 12	0.255	NEG	0.331	NEG	True_Neg
447	PLATE 12	0.387	NEG	0.410	NEG	True_Neg
448	PLATE 12	0.439	NEG	0.437	NEG	True_Neg
449	PLATE 12	0.379	NEG	0.369	NEG	True_Neg
455	PLATE 12	0.332	NEG	0.326	NEG	True_Neg
457	PLATE 12	0.341	NEG	0.390	NEG	True_Neg
460	PLATE 12	0.687	NEG	0.736	NEG	True_Neg
463	PLATE 12	0.472	NEG	0.435	NEG	True_Neg
464	PLATE 27	0.224	NEG	0.288	NEG	True_Neg
467	PLATE 12	0.450	NEG	0.451	NEG	True_Neg
475	PLATE 13	0.129	NEG	0.178	NEG	True_Neg
476	PLATE 13	0.092	NEG	0.141	NEG	True_Neg
477	PLATE 13	0.070	NEG	0.141	NEG	True_Neg
478	PLATE 13	0.287	NEG	0.381	NEG	True_Neg
479	PLATE 13	0.239	NEG	0.263	NEG	True_Neg
480	PLATE 13	0.210	NEG	0.224	NEG	True_Neg
481	PLATE 13	0.179	NEG	0.182	NEG	True_Neg
482	PLATE 13	0.461	NEG	0.419	NEG	True_Neg
483	PLATE 13	0.392	NEG	0.357	NEG	True_Neg
484	PLATE 13	0.369	NEG	0.329	NEG	True_Neg
485	PLATE 13	0.228	NEG	0.196	NEG	True_Neg
486	PLATE 13	0.432	NEG	0.398	NEG	True_Neg
487	PLATE 13	0.226	NEG	0.221	NEG	True_Neg
488	PLATE 13	0.485	NEG	0.608	NEG	True_Neg
489	PLATE 13	0.156	NEG	0.184	NEG	True_Neg
490	PLATE 13	0.179	NEG	0.169	NEG	True_Neg
491	PLATE 13	0.343	NEG	0.289	NEG	True_Neg
492	PLATE 13	0.330	NEG	0.292	NEG	True_Neg
494	PLATE 27	0.382	NEG	0.385	NEG	True_Neg
495	PLATE 13	0.321	NEG	0.265	NEG	True_Neg
496	PLATE 13	0.770	NEG	0.718	NEG	True_Neg
498	PLATE 13	0.276	NEG	0.219	NEG	True_Neg
499	PLATE 13	0.401	NEG	0.376	NEG	True_Neg
500	PLATE 13	0.138	NEG	0.199	NEG	True_Neg

502	PLATE 13	0.667	NEG	0.803	NEG	True_Neg
507	PLATE 13	0.642	NEG	0.611	NEG	True_Neg
509	PLATE 27	0.795	NEG	0.723	NEG	True_Neg
510	PLATE 13	0.201	NEG	0.151	NEG	True_Neg
511	PLATE 13	0.278	NEG	0.257	NEG	True_Neg
512	PLATE 14	0.280	NEG	0.307	NEG	True_Neg
516	PLATE 14	0.216	NEG	0.242	NEG	True_Neg
524	PLATE 14	0.698	NEG	0.650	NEG	True_Neg
525	PLATE 14	0.362	NEG	0.330	NEG	True_Neg
526	PLATE 14	0.372	NEG	0.334	NEG	True_Neg
528	PLATE 14	0.596	NEG	0.602	NEG	True_Neg
529	PLATE 14	0.411	NEG	0.369	NEG	True_Neg
530	PLATE 14	0.343	NEG	0.302	NEG	True_Neg
535	PLATE 14	0.518	NEG	0.467	NEG	True_Neg
536	PLATE 14	0.575	NEG	0.492	NEG	True_Neg
539	PLATE 14	0.289	NEG	0.266	NEG	True_Neg
540	PLATE 14	0.271	NEG	0.280	NEG	True_Neg
544	PLATE 14	0.346	NEG	0.326	NEG	True_Neg
545	PLATE 14	0.734	NEG	0.708	NEG	True_Neg
546	PLATE 27	0.496	NEG	0.461	NEG	True_Neg
547	PLATE 14	0.397	NEG	0.347	NEG	True_Neg
549	PLATE 14	0.626	NEG	0.563	NEG	True_Neg
553	PLATE 15	0.738	NEG	0.850	POS	False_Pos
556	PLATE 15	0.736	NEG	0.783	NEG	True_Neg
557	PLATE 15	0.248	NEG	0.313	NEG	True_Neg
559	PLATE 15	0.076	NEG	0.162	NEG	True_Neg
560	PLATE 15	0.149	NEG	0.230	NEG	True_Neg
561	PLATE 15	0.803	NEG	0.930	POS	False_Pos
562	PLATE 15	0.225	NEG	0.277	NEG	True_Neg
563	PLATE 15	0.271	NEG	0.306	NEG	True_Neg
564	PLATE 15	0.522	NEG	0.581	NEG	True_Neg
565	PLATE 15	0.185	NEG	0.234	NEG	True_Neg
570	PLATE 15	0.362	NEG	0.352	NEG	True_Neg
573	PLATE 15	0.183	NEG	0.264	NEG	True_Neg
576	PLATE 15	0.173	NEG	0.222	NEG	True_Neg
577	PLATE 15	0.326	NEG	0.330	NEG	True_Neg
579	PLATE 15	0.227	NEG	0.263	NEG	True_Neg
586	PLATE 15	0.201	NEG	0.218	NEG	True_Neg
587	PLATE 15	0.377	NEG	0.460	NEG	True_Neg
588	PLATE 15	0.148	NEG	0.222	NEG	True_Neg
590	PLATE 15	0.308	NEG	0.343	NEG	True_Neg
591	PLATE 15	0.270	NEG	0.295	NEG	True_Neg
592	PLATE 15	0.326	NEG	0.346	NEG	True_Neg
594	PLATE 15	0.612	NEG	0.559	NEG	True_Neg
596	PLATE 15	0.224	NEG	0.229	NEG	True_Neg
597	PLATE 15	0.688	NEG	0.688	NEG	True_Neg
598	PLATE 15	0.210	NEG	0.199	NEG	True_Neg
599	PLATE 16	0.221	NEG	0.273	NEG	True_Neg
600	PLATE 16	0.149	NEG	0.216	NEG	True_Neg
601	PLATE 16	0.781	NEG	0.883	POS	False_Pos

602	PLATE 16	0.208	NEG	0.267	NEG	True_Neg
603	PLATE 16	0.591	NEG	0.690	NEG	True_Neg
604	PLATE 16	0.181	NEG	0.228	NEG	True_Neg
605	PLATE 16	0.325	NEG	0.349	NEG	True_Neg
606	PLATE 16	0.165	NEG	0.223	NEG	True_Neg
608	PLATE 16	0.633	NEG	0.661	NEG	True_Neg
609	PLATE 16	0.319	NEG	0.349	NEG	True_Neg
610	PLATE 16	0.138	NEG	0.194	NEG	True_Neg
613	PLATE 16	0.294	NEG	0.326	NEG	True_Neg
616	PLATE 16	0.460	NEG	0.468	NEG	True_Neg
619	PLATE 16	0.136	NEG	0.200	NEG	True_Neg
622	PLATE 16	0.768	NEG	0.774	NEG	True_Neg
623	PLATE 16	0.260	NEG	0.252	NEG	True_Neg
624	PLATE 16	0.773	NEG	0.767	NEG	True_Neg
625	PLATE 27	0.613	NEG	0.608	NEG	True_Neg
627	PLATE 16	0.357	NEG	0.350	NEG	True_Neg
628	PLATE 16	0.678	NEG	0.789	NEG	True_Neg
630	PLATE 16	0.356	NEG	0.350	NEG	True_Neg
633	PLATE 16	0.395	NEG	0.414	NEG	True_Neg
634	PLATE 16	0.205	NEG	0.240	NEG	True_Neg
637	PLATE 16	0.207	NEG	0.225	NEG	True_Neg
638	PLATE 16	0.232	NEG	0.240	NEG	True_Neg
639	PLATE 16	0.241	NEG	0.234	NEG	True_Neg
640	PLATE 17	0.080	NEG	0.172	NEG	True_Neg
641	PLATE 17	0.778	NEG	0.919	POS	False_Pos
643	PLATE 27	0.578	NEG	0.579	NEG	True_Neg
647	PLATE 17	0.142	NEG	0.198	NEG	True_Neg
651	PLATE 17	0.100	NEG	0.140	NEG	True_Neg
652	PLATE 17	0.555	NEG	0.506	NEG	True_Neg
653	PLATE 17	0.535	NEG	0.505	NEG	True_Neg
654	PLATE 17	0.176	NEG	0.203	NEG	True_Neg
655	PLATE 17	0.264	NEG	0.245	NEG	True_Neg
656	PLATE 17	0.305	NEG	0.273	NEG	True_Neg
657	PLATE 17	0.237	NEG	0.213	NEG	True_Neg
658	PLATE 17	0.266	NEG	0.252	NEG	True_Neg
659	PLATE 17	0.798	NEG	0.932	POS	False_Pos
660	PLATE 17	0.176	NEG	0.188	NEG	True_Neg
661	PLATE 17	0.166	NEG	0.178	NEG	True_Neg
666	PLATE 17	0.419	NEG	0.364	NEG	True_Neg
667	PLATE 17	0.369	NEG	0.337	NEG	True_Neg
668	PLATE 17	0.457	NEG	0.405	NEG	True_Neg
669	PLATE 17	0.238	NEG	0.201	NEG	True_Neg
672	PLATE 17	0.250	NEG	0.203	NEG	True_Neg
673	PLATE 17	0.433	NEG	0.381	NEG	True_Neg
674	PLATE 17	0.475	NEG	0.523	NEG	True_Neg
675	PLATE 17	0.318	NEG	0.304	NEG	True_Neg
676	PLATE 17	0.304	NEG	0.272	NEG	True_Neg
677	PLATE 17	0.686	NEG	0.642	NEG	True_Neg
678	PLATE 28	0.534	NEG	0.652	NEG	True_Neg
682	PLATE 28	0.114	NEG	0.198	NEG	True_Neg

684	PLATE 17	0.326	NEG	0.295	NEG	True_Neg
685	PLATE 17	0.467	NEG	0.417	NEG	True_Neg
686	PLATE 17	0.239	NEG	0.200	NEG	True_Neg
687	PLATE 17	0.293	NEG	0.242	NEG	True_Neg
688	PLATE 17	0.178	NEG	0.157	NEG	True_Neg
689	PLATE 17	0.202	NEG	0.165	NEG	True_Neg
690	PLATE 18	0.353	NEG	0.354	NEG	True_Neg
691	PLATE 18	0.565	NEG	0.581	NEG	True_Neg
692	PLATE 18	0.422	NEG	0.393	NEG	True_Neg
693	PLATE 28	0.601	NEG	0.698	NEG	True_Neg
694	PLATE 18	0.245	NEG	0.249	NEG	True_Neg
695	PLATE 18	0.248	NEG	0.266	NEG	True_Neg
696	PLATE 18	0.642	NEG	0.653	NEG	True_Neg
697	PLATE 18	0.295	NEG	0.267	NEG	True_Neg
698	PLATE 18	0.225	NEG	0.212	NEG	True_Neg
699	PLATE 18	0.297	NEG	0.252	NEG	True_Neg
700	PLATE 18	0.283	NEG	0.253	NEG	True_Neg
701	PLATE 18	0.252	NEG	0.227	NEG	True_Neg
702	PLATE 18	0.445	NEG	0.367	NEG	True_Neg
703	PLATE 18	0.266	NEG	0.227	NEG	True_Neg
704	PLATE 18	0.360	NEG	0.319	NEG	True_Neg
706	PLATE 18	0.431	NEG	0.408	NEG	True_Neg
707	PLATE 28	0.381	NEG	0.432	NEG	True_Neg
708	PLATE 18	0.328	NEG	0.263	NEG	True_Neg
709	PLATE 18	0.592	NEG	0.510	NEG	True_Neg
710	PLATE 18	0.237	NEG	0.207	NEG	True_Neg
711	PLATE 18	0.366	NEG	0.296	NEG	True_Neg
712	PLATE 18	0.347	NEG	0.284	NEG	True_Neg
713	PLATE 18	0.458	NEG	0.361	NEG	True_Neg
714	PLATE 18	0.726	NEG	0.615	NEG	True_Neg
715	PLATE 28	0.517	NEG	0.606	NEG	True_Neg
717	PLATE 18	0.290	NEG	0.252	NEG	True_Neg
718	PLATE 18	0.240	NEG	0.234	NEG	True_Neg
719	PLATE 18	0.277	NEG	0.243	NEG	True_Neg
720	PLATE 28	0.299	NEG	0.378	NEG	True_Neg
721	PLATE 18	0.299	NEG	0.241	NEG	True_Neg
722	PLATE 18	0.353	NEG	0.284	NEG	True_Neg
723	PLATE 18	0.311	NEG	0.252	NEG	True_Neg
724	PLATE 18	0.757	NEG	0.668	NEG	True_Neg
726	PLATE 28	0.729	NEG	0.859	POS	False_Pos
730	PLATE 18	0.447	NEG	0.374	NEG	True_Neg
731	PLATE 28	0.578	NEG	0.569	NEG	True_Neg
732	PLATE 19	0.227	NEG	0.330	NEG	True_Neg
733	PLATE 19	0.603	NEG	0.745	NEG	True_Neg
734	PLATE 19	0.146	NEG	0.224	NEG	True_Neg
736	PLATE 19	0.093	NEG	0.191	NEG	True_Neg
737	PLATE 19	0.260	NEG	0.342	NEG	True_Neg
738	PLATE 19	0.511	NEG	0.622	NEG	True_Neg
740	PLATE 19	0.287	NEG	0.356	NEG	True_Neg
741	PLATE 19	0.157	NEG	0.220	NEG	True_Neg

742	PLATE 19	0.257	NEG	0.279	NEG	True_Neg
743	PLATE 19	0.252	NEG	0.274	NEG	True_Neg
744	PLATE 19	0.473	NEG	0.448	NEG	True_Neg
745	PLATE 19	0.242	NEG	0.249	NEG	True_Neg
746	PLATE 19	0.190	NEG	0.204	NEG	True_Neg
747	PLATE 19	0.364	NEG	0.340	NEG	True_Neg
749	PLATE 19	0.247	NEG	0.316	NEG	True_Neg
750	PLATE 19	0.541	NEG	0.605	NEG	True_Neg
751	PLATE 28	0.375	NEG	0.396	NEG	True_Neg
752	PLATE 19	0.446	NEG	0.444	NEG	True_Neg
753	PLATE 19	0.281	NEG	0.279	NEG	True_Neg
754	PLATE 19	0.384	NEG	0.358	NEG	True_Neg
755	PLATE 19	0.463	NEG	0.425	NEG	True_Neg
756	PLATE 19	0.328	NEG	0.295	NEG	True_Neg
759	PLATE 19	0.731	NEG	0.790	NEG	True_Neg
764	PLATE 28	0.651	NEG	0.674	NEG	True_Neg
767	PLATE 19	0.462	NEG	0.433	NEG	True_Neg
768	PLATE 19	0.654	NEG	0.620	NEG	True_Neg
769	PLATE 19	0.243	NEG	0.219	NEG	True_Neg
770	PLATE 19	0.291	NEG	0.228	NEG	True_Neg
774	PLATE 20	0.700	NEG	0.850	POS	False_Pos
775	PLATE 20	0.071	NEG	0.174	NEG	True_Neg
776	PLATE 20	0.284	NEG	0.350	NEG	True_Neg
777	PLATE 20	0.259	NEG	0.315	NEG	True_Neg
780	PLATE 20	0.551	NEG	0.540	NEG	True_Neg
781	PLATE 20	0.154	NEG	0.218	NEG	True_Neg
783	PLATE 20	0.300	NEG	0.307	NEG	True_Neg
784	PLATE 20	0.233	NEG	0.239	NEG	True_Neg
785	PLATE 20	0.769	NEG	0.784	NEG	True_Neg
787	PLATE 20	0.617	NEG	0.685	NEG	True_Neg
789	PLATE 20	0.601	NEG	0.581	NEG	True_Neg
790	PLATE 20	0.670	NEG	0.634	NEG	True_Neg
791	PLATE 20	0.246	NEG	0.251	NEG	True_Neg
792	PLATE 20	0.606	NEG	0.565	NEG	True_Neg
794	PLATE 20	0.717	NEG	0.686	NEG	True_Neg
797	PLATE 20	0.510	NEG	0.511	NEG	True_Neg
799	PLATE 20	0.272	NEG	0.302	NEG	True_Neg
802	PLATE 20	0.319	NEG	0.296	NEG	True_Neg
804	PLATE 20	0.300	NEG	0.279	NEG	True_Neg
806	PLATE 28	0.558	NEG	0.509	NEG	True_Neg
808	PLATE 20	0.183	NEG	0.200	NEG	True_Neg
809	PLATE 21	0.019	NEG	0.110	NEG	True_Neg
810	PLATE 21	0.478	NEG	0.510	NEG	True_Neg
811	PLATE 21	0.213	NEG	0.248	NEG	True_Neg
812	PLATE 21	0.166	NEG	0.206	NEG	True_Neg
813	PLATE 21	0.282	NEG	0.318	NEG	True_Neg
814	PLATE 21	0.426	NEG	0.459	NEG	True_Neg
815	PLATE 21	0.210	NEG	0.219	NEG	True_Neg
816	PLATE 21	0.522	NEG	0.507	NEG	True_Neg
817	PLATE 21	0.600	NEG	0.549	NEG	True_Neg

819	PLATE 21	0.733	NEG	0.723	NEG	True_Neg
822	PLATE 21	0.134	NEG	0.143	NEG	True_Neg
823	PLATE 21	0.219	NEG	0.207	NEG	True_Neg
824	PLATE 21	0.204	NEG	0.224	NEG	True_Neg
825	PLATE 21	0.500	NEG	0.462	NEG	True_Neg
826	PLATE 21	0.361	NEG	0.331	NEG	True_Neg
827	PLATE 21	0.627	NEG	0.554	NEG	True_Neg
828	PLATE 21	0.678	NEG	0.638	NEG	True_Neg
829	PLATE 21	0.324	NEG	0.303	NEG	True_Neg
830	PLATE 21	0.642	NEG	0.584	NEG	True_Neg
833	PLATE 21	0.487	NEG	0.457	NEG	True_Neg
834	PLATE 21	0.136	NEG	0.168	NEG	True_Neg
835	PLATE 21	0.588	NEG	0.586	NEG	True_Neg
836	PLATE 21	0.144	NEG	0.159	NEG	True_Neg
838	PLATE 28	0.669	NEG	0.675	NEG	True_Neg
839	PLATE 28	0.302	NEG	0.322	NEG	True_Neg
841	PLATE 21	0.400	NEG	0.342	NEG	True_Neg
842	PLATE 21	0.170	NEG	0.173	NEG	True_Neg
843	PLATE 28	0.213	NEG	0.275	NEG	True_Neg
845	PLATE 21	0.214	NEG	0.224	NEG	True_Neg
847	PLATE 28	0.498	NEG	0.520	NEG	True_Neg
850	PLATE 21	0.148	NEG	0.167	NEG	True_Neg
851	PLATE 21	0.206	NEG	0.193	NEG	True_Neg
852	PLATE 21	0.109	NEG	0.122	NEG	True_Neg
854	PLATE 28	0.457	NEG	0.454	NEG	True_Neg
855	PLATE 28	0.321	NEG	0.336	NEG	True_Neg
856	PLATE 28	0.426	NEG	0.450	NEG	True_Neg
857	PLATE 22	0.147	NEG	0.201	NEG	True_Neg
859	PLATE 28	0.272	NEG	0.271	NEG	True_Neg
860	PLATE 22	0.652	NEG	0.728	NEG	True_Neg
863	PLATE 28	0.345	NEG	0.323	NEG	True_Neg
2	PLATE 01	1.057	POS	1.261	POS	True_Pos
10	PLATE 01	0.822	POS	0.842	POS	True_Pos
11	PLATE 28	1.230	POS	1.401	POS	True_Pos
12	PLATE 28	1.208	POS	1.254	POS	True_Pos
13	PLATE 01	0.843	POS	0.831	POS	True_Pos
15	PLATE 01	1.072	POS	1.121	POS	True_Pos
16	PLATE 01	0.939	POS	0.955	POS	True_Pos
17	PLATE 01	1.947	POS	2.525	POS	True_Pos
18	PLATE 01	1.511	POS	2.181	POS	True_Pos
22	PLATE 01	2.089	POS	2.342	POS	True_Pos
23	PLATE 01	1.244	POS	1.326	POS	True_Pos
24	PLATE 01	1.062	POS	1.141	POS	True_Pos
25	PLATE 01	1.501	POS	1.699	POS	True_Pos
27	PLATE 01	1.446	POS	1.750	POS	True_Pos
29	PLATE 01	2.354	POS	2.620	POS	True_Pos
30	PLATE 01	0.967	POS	1.126	POS	True_Pos
31	PLATE 01	1.499	POS	1.777	POS	True_Pos
32	PLATE 01	0.901	POS	0.946	POS	True_Pos
33	PLATE 01	1.128	POS	1.272	POS	True_Pos

34	PLATE 01	1.041	POS	1.143	POS	True_Pos
36	PLATE 01	1.703	POS	2.035	POS	True_Pos
38	PLATE 01	1.198	POS	1.361	POS	True_Pos
39	PLATE 01	0.878	POS	1.039	POS	True_Pos
40	PLATE 02	1.575	POS	2.219	POS	True_Pos
45	PLATE 02	0.942	POS	1.189	POS	True_Pos
46	PLATE 28	1.207	POS	1.353	POS	True_Pos
47	PLATE 02	0.972	POS	1.071	POS	True_Pos
48	PLATE 02	1.796	POS	2.096	POS	True_Pos
49	PLATE 02	2.259	POS	2.684	POS	True_Pos
52	PLATE 02	0.821	POS	0.805	NEG	False_Neg
53	PLATE 02	0.932	POS	0.988	POS	True_Pos
54	PLATE 02	0.927	POS	1.072	POS	True_Pos
55	PLATE 02	1.381	POS	1.925	POS	True_Pos
56	PLATE 02	1.753	POS	2.393	POS	True_Pos
59	PLATE 02	0.898	POS	0.904	POS	True_Pos
61	PLATE 02	1.040	POS	1.045	POS	True_Pos
63	PLATE 02	0.961	POS	1.023	POS	True_Pos
65	PLATE 02	1.239	POS	1.797	POS	True_Pos
67	PLATE 02	1.505	POS	1.952	POS	True_Pos
75	PLATE 02	0.986	POS	1.212	POS	True_Pos
77	PLATE 03	1.241	POS	1.589	POS	True_Pos
78	PLATE 03	2.072	POS	2.456	POS	True_Pos
84	PLATE 28	1.340	POS	1.460	POS	True_Pos
85	PLATE 29	1.027	POS	0.976	POS	True_Pos
86	PLATE 28	0.927	POS	1.031	POS	True_Pos
87	PLATE 03	2.283	POS	2.307	POS	True_Pos
88	PLATE 03	1.209	POS	1.300	POS	True_Pos
93	PLATE 03	0.848	POS	0.878	POS	True_Pos
122	PLATE 04	1.260	POS	1.350	POS	True_Pos
123	PLATE 04	1.590	POS	1.732	POS	True_Pos
126	PLATE 04	1.924	POS	2.552	POS	True_Pos
129	PLATE 04	1.820	POS	2.635	POS	True_Pos
130	PLATE 04	0.928	POS	0.938	POS	True_Pos
131	PLATE 04	0.808	POS	0.766	NEG	False_Neg
132	PLATE 04	1.212	POS	1.303	POS	True_Pos
134	PLATE 04	1.014	POS	1.005	POS	True_Pos
135	PLATE 04	0.954	POS	0.984	POS	True_Pos
136	PLATE 28	1.079	POS	1.321	POS	True_Pos
145	PLATE 04	1.614	POS	2.264	POS	True_Pos
150	PLATE 05	1.507	POS	2.062	POS	True_Pos
153	PLATE 05	1.822	POS	2.400	POS	True_Pos
154	PLATE 05	1.767	POS	2.775	POS	True_Pos
158	PLATE 05	1.061	POS	1.287	POS	True_Pos
167	PLATE 05	1.096	POS	1.499	POS	True_Pos
171	PLATE 05	1.641	POS	1.966	POS	True_Pos
176	PLATE 05	1.712	POS	2.072	POS	True_Pos
178	PLATE 05	0.869	POS	1.056	POS	True_Pos
182	PLATE 05	1.545	POS	2.018	POS	True_Pos
192	PLATE 06	0.871	POS	1.025	POS	True_Pos

193	PLATE 06	1.468	POS	1.899	POS	True_Pos
195	PLATE 06	2.061	POS	3.219	POS	True_Pos
197	PLATE 06	1.587	POS	1.907	POS	True_Pos
202	PLATE 06	1.060	POS	1.079	POS	True_Pos
205	PLATE 06	1.185	POS	1.669	POS	True_Pos
210	PLATE 06	1.702	POS	1.880	POS	True_Pos
213	PLATE 06	1.186	POS	1.274	POS	True_Pos
216	PLATE 06	0.862	POS	0.933	POS	True_Pos
219	PLATE 06	1.827	POS	2.065	POS	True_Pos
220	PLATE 27	1.113	POS	1.182	POS	True_Pos
222	PLATE 06	1.019	POS	1.090	POS	True_Pos
223	PLATE 06	2.294	POS	2.804	POS	True_Pos
227	PLATE 27	1.387	POS	1.563	POS	True_Pos
249	PLATE 27	0.869	POS	1.014	POS	True_Pos
256	PLATE 07	1.258	POS	1.593	POS	True_Pos
257	PLATE 07	0.827	POS	0.903	POS	True_Pos
262	PLATE 07	1.433	POS	1.601	POS	True_Pos
263	PLATE 07	0.986	POS	1.046	POS	True_Pos
266	PLATE 08	2.065	POS	2.767	POS	True_Pos
267	PLATE 08	1.905	POS	2.496	POS	True_Pos
268	PLATE 08	1.271	POS	1.664	POS	True_Pos
269	PLATE 08	1.880	POS	2.618	POS	True_Pos
271	PLATE 08	0.902	POS	1.171	POS	True_Pos
273	PLATE 08	1.697	POS	2.172	POS	True_Pos
274	PLATE 08	1.009	POS	1.104	POS	True_Pos
275	PLATE 08	1.792	POS	2.070	POS	True_Pos
277	PLATE 08	0.971	POS	1.028	POS	True_Pos
279	PLATE 08	1.646	POS	1.923	POS	True_Pos
280	PLATE 08	1.820	POS	2.336	POS	True_Pos
281	PLATE 08	1.986	POS	2.995	POS	True_Pos
282	PLATE 08	0.967	POS	1.240	POS	True_Pos
283	PLATE 08	1.732	POS	2.271	POS	True_Pos
284	PLATE 08	1.698	POS	2.062	POS	True_Pos
285	PLATE 08	0.832	POS	0.786	NEG	False_Neg
286	PLATE 08	1.354	POS	1.498	POS	True_Pos
292	PLATE 08	0.943	POS	1.206	POS	True_Pos
294	PLATE 08	1.507	POS	1.898	POS	True_Pos
295	PLATE 08	0.911	POS	0.909	POS	True_Pos
296	PLATE 08	1.693	POS	2.039	POS	True_Pos
300	PLATE 08	1.146	POS	1.282	POS	True_Pos
301	PLATE 08	1.197	POS	1.392	POS	True_Pos
302	PLATE 08	1.026	POS	1.307	POS	True_Pos
306	PLATE 09	0.907	POS	1.138	POS	True_Pos
308	PLATE 09	1.014	POS	1.308	POS	True_Pos
309	PLATE 09	0.868	POS	1.033	POS	True_Pos
310	PLATE 09	1.999	POS	2.496	POS	True_Pos
311	PLATE 09	1.340	POS	1.486	POS	True_Pos
314	PLATE 09	1.020	POS	1.114	POS	True_Pos
315	PLATE 09	1.471	POS	1.872	POS	True_Pos
316	PLATE 09	0.856	POS	1.004	POS	True_Pos

319	PLATE 09	0.874	POS	1.037	POS	True_Pos
323	PLATE 09	1.119	POS	1.137	POS	True_Pos
329	PLATE 09	1.324	POS	1.813	POS	True_Pos
332	PLATE 09	0.837	POS	0.907	POS	True_Pos
338	PLATE 25	1.752	POS	2.241	POS	True_Pos
339	PLATE 25	1.511	POS	1.959	POS	True_Pos
342	PLATE 09	2.137	POS	3.252	POS	True_Pos
344	PLATE 25	1.856	POS	2.426	POS	True_Pos
345	PLATE 25	1.668	POS	2.324	POS	True_Pos
346	PLATE 25	1.872	POS	2.251	POS	True_Pos
347	PLATE 25	1.167	POS	1.336	POS	True_Pos
348	PLATE 25	1.771	POS	2.040	POS	True_Pos
349	PLATE 25	1.355	POS	1.436	POS	True_Pos
350	PLATE 10	1.427	POS	1.934	POS	True_Pos
351	PLATE 10	1.463	POS	1.977	POS	True_Pos
352	PLATE 10	1.105	POS	1.453	POS	True_Pos
355	PLATE 10	0.928	POS	1.113	POS	True_Pos
361	PLATE 25	1.890	POS	2.144	POS	True_Pos
370	PLATE 10	1.219	POS	1.457	POS	True_Pos
372	PLATE 10	1.797	POS	2.154	POS	True_Pos
374	PLATE 10	1.610	POS	1.818	POS	True_Pos
375	PLATE 10	1.349	POS	1.547	POS	True_Pos
376	PLATE 10	0.905	POS	0.978	POS	True_Pos
381	PLATE 10	1.274	POS	1.561	POS	True_Pos
382	PLATE 10	0.829	POS	0.861	POS	True_Pos
387	PLATE 10	1.136	POS	1.281	POS	True_Pos
390	PLATE 11	0.835	POS	1.000	POS	True_Pos
391	PLATE 11	0.988	POS	1.143	POS	True_Pos
392	PLATE 11	1.467	POS	1.831	POS	True_Pos
413	PLATE 11	1.020	POS	0.985	POS	True_Pos
415	PLATE 11	0.838	POS	0.929	POS	True_Pos
429	PLATE 27	1.146	POS	1.237	POS	True_Pos
434	PLATE 12	1.044	POS	1.450	POS	True_Pos
438	PLATE 12	1.196	POS	1.430	POS	True_Pos
440	PLATE 12	0.838	POS	0.895	POS	True_Pos
441	PLATE 12	1.125	POS	1.282	POS	True_Pos
444	PLATE 12	1.254	POS	1.515	POS	True_Pos
450	PLATE 12	1.039	POS	1.115	POS	True_Pos
451	PLATE 12	1.491	POS	1.892	POS	True_Pos
452	PLATE 12	1.839	POS	2.101	POS	True_Pos
453	PLATE 12	1.811	POS	2.133	POS	True_Pos
454	PLATE 12	1.692	POS	2.061	POS	True_Pos
456	PLATE 12	1.161	POS	1.573	POS	True_Pos
458	PLATE 12	0.968	POS	1.142	POS	True_Pos
459	PLATE 12	0.957	POS	1.033	POS	True_Pos
461	PLATE 12	2.007	POS	2.359	POS	True_Pos
462	PLATE 12	1.770	POS	2.126	POS	True_Pos
468	PLATE 12	0.850	POS	0.964	POS	True_Pos
470	PLATE 13	1.137	POS	1.556	POS	True_Pos
473	PLATE 13	0.884	POS	1.139	POS	True_Pos

474	PLATE 27	1.087	POS	1.409	POS	True_Pos
493	PLATE 27	0.956	POS	0.996	POS	True_Pos
497	PLATE 13	0.818	POS	0.769	NEG	False_Neg
503	PLATE 13	1.819	POS	2.324	POS	True_Pos
504	PLATE 13	1.579	POS	1.830	POS	True_Pos
506	PLATE 13	0.895	POS	0.934	POS	True_Pos
508	PLATE 13	0.866	POS	0.875	POS	True_Pos
514	PLATE 14	0.952	POS	1.046	POS	True_Pos
515	PLATE 14	2.041	POS	2.356	POS	True_Pos
517	PLATE 14	1.319	POS	1.642	POS	True_Pos
518	PLATE 14	1.453	POS	1.682	POS	True_Pos
519	PLATE 14	1.139	POS	1.247	POS	True_Pos
520	PLATE 14	1.341	POS	1.412	POS	True_Pos
521	PLATE 14	1.389	POS	1.417	POS	True_Pos
522	PLATE 14	1.652	POS	1.776	POS	True_Pos
523	PLATE 14	1.831	POS	2.042	POS	True_Pos
527	PLATE 14	0.976	POS	1.107	POS	True_Pos
531	PLATE 14	1.199	POS	1.169	POS	True_Pos
532	PLATE 14	1.786	POS	1.925	POS	True_Pos
537	PLATE 14	1.042	POS	0.988	POS	True_Pos
538	PLATE 14	1.437	POS	1.595	POS	True_Pos
541	PLATE 14	0.975	POS	1.042	POS	True_Pos
542	PLATE 14	2.077	POS	2.399	POS	True_Pos
543	PLATE 14	1.981	POS	2.167	POS	True_Pos
550	PLATE 14	1.060	POS	1.090	POS	True_Pos
551	PLATE 25	1.141	POS	1.196	POS	True_Pos
552	PLATE 14	1.393	POS	1.700	POS	True_Pos
555	PLATE 15	1.305	POS	1.476	POS	True_Pos
566	PLATE 15	1.646	POS	1.850	POS	True_Pos
571	PLATE 15	1.398	POS	1.544	POS	True_Pos
572	PLATE 15	1.349	POS	1.666	POS	True_Pos
575	PLATE 15	1.285	POS	1.580	POS	True_Pos
578	PLATE 15	1.873	POS	2.153	POS	True_Pos
580	PLATE 15	1.275	POS	1.341	POS	True_Pos
584	PLATE 15	0.983	POS	0.960	POS	True_Pos
585	PLATE 15	1.264	POS	1.339	POS	True_Pos
589	PLATE 27	0.843	POS	0.864	POS	True_Pos
593	PLATE 25	1.919	POS	2.547	POS	True_Pos
595	PLATE 15	1.613	POS	1.859	POS	True_Pos
607	PLATE 16	0.869	POS	0.898	POS	True_Pos
611	PLATE 16	0.942	POS	0.980	POS	True_Pos
612	PLATE 16	0.957	POS	1.033	POS	True_Pos
615	PLATE 16	0.964	POS	1.127	POS	True_Pos
617	PLATE 16	1.411	POS	1.627	POS	True_Pos
618	PLATE 16	1.495	POS	1.654	POS	True_Pos
620	PLATE 25	0.884	POS	1.036	POS	True_Pos
621	PLATE 16	1.678	POS	1.987	POS	True_Pos
629	PLATE 16	1.165	POS	1.456	POS	True_Pos
631	PLATE 16	1.985	POS	2.278	POS	True_Pos
636	PLATE 16	1.326	POS	1.444	POS	True_Pos

644	PLATE 17	1.294	POS	1.556	POS	True_Pos
646	PLATE 17	1.439	POS	1.788	POS	True_Pos
648	PLATE 17	1.004	POS	1.176	POS	True_Pos
650	PLATE 17	1.092	POS	1.306	POS	True_Pos
670	PLATE 25	1.664	POS	1.877	POS	True_Pos
671	PLATE 17	1.159	POS	1.147	POS	True_Pos
705	PLATE 18	0.874	POS	0.966	POS	True_Pos
716	PLATE 18	0.859	POS	0.816	POS	True_Pos
725	PLATE 18	1.639	POS	1.762	POS	True_Pos
727	PLATE 18	1.871	POS	2.150	POS	True_Pos
728	PLATE 26	1.750	POS	2.156	POS	True_Pos
729	PLATE 28	0.894	POS	1.004	POS	True_Pos
735	PLATE 26	0.977	POS	1.169	POS	True_Pos
748	PLATE 19	0.825	POS	0.886	POS	True_Pos
757	PLATE 19	1.028	POS	0.978	POS	True_Pos
758	PLATE 19	1.421	POS	1.573	POS	True_Pos
760	PLATE 19	0.864	POS	1.153	POS	True_Pos
761	PLATE 19	1.930	POS	2.539	POS	True_Pos
762	PLATE 19	1.332	POS	1.612	POS	True_Pos
763	PLATE 19	1.354	POS	1.497	POS	True_Pos
765	PLATE 19	1.383	POS	1.540	POS	True_Pos
766	PLATE 19	0.817	POS	0.910	POS	True_Pos
771	PLATE 20	1.580	POS	2.089	POS	True_Pos
772	PLATE 20	1.222	POS	1.660	POS	True_Pos
773	PLATE 20	1.693	POS	2.255	POS	True_Pos
778	PLATE 26	1.590	POS	1.969	POS	True_Pos
779	PLATE 20	0.817	POS	0.885	POS	True_Pos
782	PLATE 20	1.616	POS	1.757	POS	True_Pos
786	PLATE 20	0.913	POS	1.060	POS	True_Pos
788	PLATE 20	1.771	POS	2.154	POS	True_Pos
795	PLATE 20	1.744	POS	1.899	POS	True_Pos
796	PLATE 20	1.422	POS	1.597	POS	True_Pos
798	PLATE 28	1.816	POS	2.014	POS	True_Pos
800	PLATE 20	1.406	POS	1.596	POS	True_Pos
801	PLATE 20	1.147	POS	1.223	POS	True_Pos
803	PLATE 20	1.524	POS	1.629	POS	True_Pos
805	PLATE 20	1.773	POS	2.002	POS	True_Pos
807	PLATE 20	0.967	POS	0.978	POS	True_Pos
818	PLATE 21	0.906	POS	0.896	POS	True_Pos
820	PLATE 21	1.140	POS	1.213	POS	True_Pos
821	PLATE 21	0.865	POS	0.832	POS	True_Pos
831	PLATE 21	1.056	POS	1.015	POS	True_Pos
832	PLATE 21	1.240	POS	1.286	POS	True_Pos
837	PLATE 26	1.196	POS	1.302	POS	True_Pos
840	PLATE 28	0.808	POS	0.933	POS	True_Pos
844	PLATE 26	1.775	POS	2.130	POS	True_Pos
846	PLATE 26	0.949	POS	1.154	POS	True_Pos
848	PLATE 26	1.364	POS	1.654	POS	True_Pos
849	PLATE 26	1.621	POS	1.869	POS	True_Pos
853	PLATE 21	1.775	POS	2.072	POS	True_Pos

858	PLATE 26	0.912	POS	0.928	POS	True_Pos
861	PLATE 26	1.795	POS	2.051	POS	True_Pos
862	PLATE 26	1.109	POS	1.351	POS	True_Pos
