# Supplementary material for: Validation of new equipment for SARS-CoV-2 diagnosis in Ecuador: Detection of the virus and antibodies generated by disease and vaccines with one POC device
Source: PLoS One. 2025 Apr 16;20(4):e0321794. doi: 10.1371/journal.pone.0321794 (PMC12002511; doi:10.1371/journal.pone.0321794)
Supplement: S6 File — (PDF) [file pone.0321794.s006.pdf]

SAMPLE	SPEC AVERAGE S-RBD-N	SPEC AVERAGE	PLUM AVERAGE S-RBD-N	PLUM AVERAGE	FINAL VALIDATION
131	1.175	POS	0.389	POS	False_Neg
285	1.466	POS	0.350	POS	False_Neg
497	1.178	POS	0.343	POS	False_Neg
4	0.795	NEG	2.433	NEG	False_Pos
8	0.691	NEG	2.486	NEG	False_Pos
42	0.942	NEG	2.470	NEG	False_Pos
183	0.845	NEG	2.511	NEG	False_Pos
190	1.023	NEG	2.060	NEG	False_Pos
250	1.010	NEG	2.510	NEG	False_Pos
272	0.892	NEG	2.537	NEG	False_Pos
321	0.860	NEG	2.533	NEG	False_Pos
353	0.896	NEG	2.455	NEG	False_Pos
481	0.777	NEG	2.463	NEG	False_Pos
485	0.801	NEG	2.506	NEG	False_Pos
560	0.542	NEG	2.510	NEG	False_Pos
598	0.738	NEG	2.461	NEG	False_Pos
659	0.855	NEG	2.470	NEG	False_Pos
686	0.606	NEG	2.455	NEG	False_Pos
1	0.404	NEG	0.508	NEG	True_Neg
7	0.474	NEG	0.523	NEG	True_Neg
9	0.373	NEG	0.386	NEG	True_Neg
14	0.533	NEG	0.603	NEG	True_Neg
19	0.591	NEG	0.605	NEG	True_Neg
20	0.355	NEG	0.342	NEG	True_Neg
21	0.919	NEG	1.056	NEG	True_Neg
26	0.739	NEG	0.741	NEG	True_Neg
28	0.581	NEG	0.589	NEG	True_Neg
37	0.576	NEG	0.578	NEG	True_Neg
41	0.529	NEG	0.544	NEG	True_Neg
43	0.591	NEG	0.620	NEG	True_Neg
44	0.471	NEG	0.495	NEG	True_Neg
50	0.509	NEG	0.487	NEG	True_Neg
51	0.535	NEG	0.497	NEG	True_Neg
57	0.849	NEG	0.901	NEG	True_Neg
58	0.813	NEG	0.794	NEG	True_Neg
60	0.632	NEG	0.622	NEG	True_Neg
62	0.778	NEG	0.752	NEG	True_Neg
64	0.523	NEG	0.533	NEG	True_Neg
66	0.579	NEG	0.584	NEG	True_Neg
68	0.900	NEG	0.916	NEG	True_Neg
69	0.621	NEG	0.601	NEG	True_Neg
72	0.758	NEG	0.728	NEG	True_Neg
73	0.627	NEG	0.580	NEG	True_Neg
74	0.531	NEG	0.532	NEG	True_Neg
76	0.627	NEG	0.698	NEG	True_Neg
81	0.400	NEG	0.415	NEG	True_Neg
82	0.518	NEG	0.526	NEG	True_Neg

83	0.757	NEG	0.801	NEG	True_Neg
185	0.401	NEG	0.442	NEG	True_Neg
187	0.794	NEG	0.883	NEG	True_Neg
188	0.296	NEG	0.321	NEG	True_Neg
189	0.390	NEG	0.435	NEG	True_Neg
191	0.286	NEG	0.266	NEG	True_Neg
194	0.255	NEG	0.312	NEG	True_Neg
196	0.200	NEG	0.228	NEG	True_Neg
198	0.554	NEG	0.577	NEG	True_Neg
199	0.550	NEG	0.539	NEG	True_Neg
229	0.166	NEG	0.225	NEG	True_Neg
235	0.791	NEG	0.904	NEG	True_Neg
247	0.684	NEG	0.689	NEG	True_Neg
248	0.287	NEG	0.289	NEG	True_Neg
251	0.786	NEG	0.829	NEG	True_Neg
252	0.748	NEG	0.852	NEG	True_Neg
254	0.181	NEG	0.202	NEG	True_Neg
255	0.256	NEG	0.300	NEG	True_Neg
259	0.462	NEG	0.406	NEG	True_Neg
264	0.360	NEG	0.396	NEG	True_Neg
276	0.711	NEG	0.749	NEG	True_Neg
293	0.723	NEG	0.827	NEG	True_Neg
297	0.522	NEG	0.501	NEG	True_Neg
298	0.473	NEG	0.442	NEG	True_Neg
324	0.739	NEG	0.740	NEG	True_Neg
325	0.511	NEG	0.481	NEG	True_Neg
328	0.436	NEG	0.421	NEG	True_Neg
330	0.641	NEG	0.631	NEG	True_Neg
331	0.606	NEG	0.628	NEG	True_Neg
333	0.452	NEG	0.451	NEG	True_Neg
334	0.530	NEG	0.534	NEG	True_Neg
335	0.764	NEG	0.805	NEG	True_Neg
340	0.657	NEG	0.664	NEG	True_Neg
359	0.123	NEG	0.203	NEG	True_Neg
363	0.473	NEG	0.532	NEG	True_Neg
367	0.766	NEG	0.992	NEG	True_Neg
371	0.642	NEG	0.693	NEG	True_Neg
408	0.652	NEG	0.651	NEG	True_Neg
425	0.616	NEG	0.740	NEG	True_Neg
443	0.787	NEG	0.914	NEG	True_Neg
447	0.388	NEG	0.363	NEG	True_Neg
457	0.647	NEG	0.869	NEG	True_Neg
475	0.645	NEG	0.796	NEG	True_Neg
477	0.236	NEG	0.226	NEG	True_Neg
479	0.448	NEG	0.469	NEG	True_Neg
489	0.727	NEG	0.920	NEG	True_Neg
492	0.606	NEG	0.605	NEG	True_Neg
496	0.636	NEG	0.553	NEG	True_Neg
510	0.332	NEG	0.311	NEG	True_Neg
525	0.273	NEG	0.161	NEG	True_Neg

539	0.491	NEG	0.484	NEG	True_Neg
540	0.191	NEG	0.047	NEG	True_Neg
544	0.461	NEG	0.738	NEG	True_Neg
557	0.345	NEG	0.352	NEG	True_Neg
559	0.264	NEG	0.265	NEG	True_Neg
563	0.531	NEG	0.547	NEG	True_Neg
576	0.005	NEG	0.160	NEG	True_Neg
579	0.418	NEG	0.449	NEG	True_Neg
588	0.531	NEG	0.624	NEG	True_Neg
591	0.164	NEG	0.178	NEG	True_Neg
602	0.673	NEG	0.782	NEG	True_Neg
604	0.157	NEG	0.025	NEG	True_Neg
606	0.598	NEG	0.886	NEG	True_Neg
610	0.505	NEG	0.545	NEG	True_Neg
619	0.118	NEG	0.012	NEG	True_Neg
630	0.328	NEG	0.190	NEG	True_Neg
634	0.263	NEG	0.215	NEG	True_Neg
654	0.582	NEG	0.896	NEG	True_Neg
660	0.413	NEG	0.409	NEG	True_Neg
733	0.515	NEG	0.762	NEG	True_Neg
736	0.272	NEG	0.323	NEG	True_Neg
743	0.354	NEG	0.841	NEG	True_Neg
756	0.550	NEG	0.940	NEG	True_Neg
768	0.581	NEG	1.128	NEG	True_Neg
770	0.610	NEG	0.582	NEG	True_Neg
781	0.355	NEG	0.364	NEG	True_Neg
787	0.983	NEG	1.103	NEG	True_Neg
811	0.543	NEG	0.928	NEG	True_Neg
817	0.789	NEG	1.063	NEG	True_Neg
835	0.930	NEG	0.959	NEG	True_Neg
852	0.535	NEG	0.632	NEG	True_Neg
11	1.540	POS	2.178	POS	True_Pos
12	1.718	POS	2.530	POS	True_Pos
22	2.279	POS	4.017	POS	True_Pos
27	1.651	POS	2.272	POS	True_Pos
29	2.204	POS	3.111	POS	True_Pos
34	1.350	POS	1.959	POS	True_Pos
36	2.102	POS	3.904	POS	True_Pos
46	1.571	POS	2.149	POS	True_Pos
48	1.899	POS	2.855	POS	True_Pos
49	2.279	POS	3.225	POS	True_Pos
56	1.925	POS	2.852	POS	True_Pos
61	1.328	POS	1.611	POS	True_Pos
65	1.675	POS	2.742	POS	True_Pos
67	1.848	POS	2.674	POS	True_Pos
77	1.724	POS	2.608	POS	True_Pos
78	1.960	POS	2.603	POS	True_Pos
84	1.750	POS	2.621	POS	True_Pos
85	1.535	POS	2.240	POS	True_Pos
86	1.580	POS	3.134	POS	True_Pos

87	2.040	POS	2.710	POS	True_Pos
88	1.467	POS	2.101	POS	True_Pos
93	1.354	POS	1.791	POS	True_Pos
122	1.968	POS	3.808	POS	True_Pos
123	2.121	POS	3.027	POS	True_Pos
126	2.217	POS	3.617	POS	True_Pos
129	2.185	POS	3.877	POS	True_Pos
130	1.562	POS	3.074	POS	True_Pos
132	1.937	POS	3.757	POS	True_Pos
134	1.793	POS	3.258	POS	True_Pos
135	1.322	POS	1.837	POS	True_Pos
136	1.562	POS	2.508	POS	True_Pos
145	2.123	POS	4.249	POS	True_Pos
150	2.032	POS	3.316	POS	True_Pos
153	2.205	POS	3.941	POS	True_Pos
154	2.158	POS	3.846	POS	True_Pos
158	1.649	POS	3.248	POS	True_Pos
167	1.846	POS	4.603	POS	True_Pos
171	2.106	POS	3.586	POS	True_Pos
176	1.604	POS	3.824	POS	True_Pos
178	1.705	POS	2.712	POS	True_Pos
182	1.550	POS	2.068	POS	True_Pos
192	1.314	POS	1.866	POS	True_Pos
193	1.724	POS	2.602	POS	True_Pos
195	1.989	POS	3.261	POS	True_Pos
197	1.720	POS	2.729	POS	True_Pos
202	1.876	POS	3.162	POS	True_Pos
205	1.411	POS	2.013	POS	True_Pos
210	2.151	POS	3.285	POS	True_Pos
213	1.937	POS	3.294	POS	True_Pos
216	1.192	POS	1.652	POS	True_Pos
219	2.278	POS	3.710	POS	True_Pos
220	1.782	POS	2.145	POS	True_Pos
222	1.763	POS	3.280	POS	True_Pos
223	2.389	POS	4.004	POS	True_Pos
227	1.968	POS	3.507	POS	True_Pos
249	1.667	POS	2.968	POS	True_Pos
256	1.521	POS	2.390	POS	True_Pos
257	1.167	POS	1.665	POS	True_Pos
262	1.732	POS	2.553	POS	True_Pos
263	1.412	POS	1.938	POS	True_Pos
266	2.145	POS	3.517	POS	True_Pos
267	2.032	POS	3.532	POS	True_Pos
268	1.454	POS	1.964	POS	True_Pos
269	1.913	POS	2.759	POS	True_Pos
271	1.449	POS	2.173	POS	True_Pos
273	2.124	POS	3.259	POS	True_Pos
274	1.605	POS	2.259	POS	True_Pos
275	1.949	POS	2.649	POS	True_Pos
277	1.555	POS	2.113	POS	True_Pos

279	1.827	POS	2.574	POS	True_Pos
280	1.789	POS	2.483	POS	True_Pos
281	2.283	POS	3.822	POS	True_Pos
282	1.476	POS	2.347	POS	True_Pos
283	2.145	POS	3.275	POS	True_Pos
284	2.129	POS	3.093	POS	True_Pos
286	1.984	POS	2.999	POS	True_Pos
292	1.514	POS	2.509	POS	True_Pos
294	1.778	POS	2.719	POS	True_Pos
295	1.561	POS	2.221	POS	True_Pos
296	1.932	POS	2.787	POS	True_Pos
300	1.738	POS	2.777	POS	True_Pos
301	1.379	POS	2.015	POS	True_Pos
302	1.530	POS	2.229	POS	True_Pos
306	1.516	POS	2.138	POS	True_Pos
308	1.434	POS	2.088	POS	True_Pos
309	1.197	POS	1.536	POS	True_Pos
310	2.107	POS	3.156	POS	True_Pos
311	1.955	POS	3.151	POS	True_Pos
314	1.379	POS	1.822	POS	True_Pos
315	1.737	POS	2.602	POS	True_Pos
316	1.329	POS	1.787	POS	True_Pos
319	1.412	POS	2.006	POS	True_Pos
323	1.778	POS	2.715	POS	True_Pos
329	1.731	POS	2.699	POS	True_Pos
332	1.191	POS	1.453	POS	True_Pos
338	1.910	POS	2.794	POS	True_Pos
339	1.820	POS	2.720	POS	True_Pos
342	2.066	POS	3.168	POS	True_Pos
344	2.121	POS	3.885	POS	True_Pos
345	2.101	POS	3.267	POS	True_Pos
346	2.216	POS	3.558	POS	True_Pos
347	1.917	POS	3.753	POS	True_Pos
348	2.065	POS	2.784	POS	True_Pos
349	1.826	POS	2.720	POS	True_Pos
350	1.722	POS	2.651	POS	True_Pos
351	1.767	POS	2.966	POS	True_Pos
352	1.690	POS	2.981	POS	True_Pos
355	1.492	POS	2.346	POS	True_Pos
361	2.310	POS	4.134	POS	True_Pos
370	1.727	POS	2.596	POS	True_Pos
372	1.766	POS	2.320	POS	True_Pos
374	1.879	POS	2.911	POS	True_Pos
375	1.443	POS	1.921	POS	True_Pos
376	1.249	POS	1.602	POS	True_Pos
381	1.786	POS	3.113	POS	True_Pos
382	1.625	POS	2.830	POS	True_Pos
387	1.797	POS	2.897	POS	True_Pos
390	1.159	POS	1.528	POS	True_Pos
391	1.336	POS	1.819	POS	True_Pos

392	1.641	POS	2.224	POS	True_Pos
413	1.468	POS	2.159	POS	True_Pos
415	1.262	POS	1.688	POS	True_Pos
429	1.798	POS	3.466	POS	True_Pos
434	1.751	POS	3.738	POS	True_Pos
438	1.901	POS	3.629	POS	True_Pos
440	1.330	POS	1.786	POS	True_Pos
441	1.574	POS	2.447	POS	True_Pos
444	1.696	POS	2.234	POS	True_Pos
450	1.241	POS	1.506	POS	True_Pos
451	1.934	POS	2.947	POS	True_Pos
452	2.109	POS	3.099	POS	True_Pos
453	2.182	POS	3.620	POS	True_Pos
454	2.023	POS	3.385	POS	True_Pos
456	1.813	POS	4.612	POS	True_Pos
458	1.378	POS	2.362	POS	True_Pos
459	1.530	POS	3.056	POS	True_Pos
461	2.145	POS	3.354	POS	True_Pos
462	2.013	POS	3.056	POS	True_Pos
468	1.487	POS	2.183	POS	True_Pos
470	1.525	POS	2.328	POS	True_Pos
473	1.227	POS	1.676	POS	True_Pos
474	1.816	POS	3.129	POS	True_Pos
493	1.685	POS	2.531	POS	True_Pos
503	2.113	POS	3.390	POS	True_Pos
504	2.025	POS	3.407	POS	True_Pos
506	1.597	POS	2.710	POS	True_Pos
508	1.597	POS	2.770	POS	True_Pos
514	1.485	POS	2.251	POS	True_Pos
515	1.963	POS	2.778	POS	True_Pos
517	1.603	POS	1.800	POS	True_Pos
518	1.828	POS	3.000	POS	True_Pos
519	1.703	POS	1.735	POS	True_Pos
520	1.759	POS	1.866	POS	True_Pos
521	1.554	POS	2.150	POS	True_Pos
523	1.906	POS	2.630	POS	True_Pos
527	1.448	POS	2.264	POS	True_Pos
531	1.390	POS	1.608	POS	True_Pos
532	1.682	POS	2.100	POS	True_Pos
537	1.320	POS	2.063	POS	True_Pos
538	1.424	POS	2.055	POS	True_Pos
541	1.528	POS	3.303	POS	True_Pos
542	2.329	POS	3.836	POS	True_Pos
543	2.047	POS	3.081	POS	True_Pos
550	1.771	POS	4.468	POS	True_Pos
551	1.745	POS	2.887	POS	True_Pos
552	1.654	POS	2.436	POS	True_Pos
555	1.653	POS	2.350	POS	True_Pos
566	1.649	POS	2.858	POS	True_Pos
571	1.674	POS	2.339	POS	True_Pos

572	1.780	POS	3.235	POS	True_Pos
575	1.689	POS	3.001	POS	True_Pos
578	1.806	POS	2.554	POS	True_Pos
580	1.461	POS	1.880	POS	True_Pos
584	1.471	POS	2.087	POS	True_Pos
585	1.630	POS	4.167	POS	True_Pos
589	1.711	POS	2.997	POS	True_Pos
593	2.213	POS	3.720	POS	True_Pos
595	2.071	POS	4.774	POS	True_Pos
607	1.411	POS	2.377	POS	True_Pos
611	1.783	POS	3.705	POS	True_Pos
612	1.588	POS	2.101	POS	True_Pos
617	1.438	POS	2.147	POS	True_Pos
618	1.896	POS	4.452	POS	True_Pos
620	1.680	POS	5.267	POS	True_Pos
621	1.583	POS	2.009	POS	True_Pos
629	1.364	POS	1.688	POS	True_Pos
631	2.037	POS	3.051	POS	True_Pos
636	1.609	POS	2.348	POS	True_Pos
644	1.518	POS	2.127	POS	True_Pos
646	1.486	POS	2.134	POS	True_Pos
648	1.218	POS	1.772	POS	True_Pos
670	1.989	POS	3.163	POS	True_Pos
671	1.341	POS	1.488	POS	True_Pos
705	1.265	POS	1.762	POS	True_Pos
716	1.634	POS	2.842	POS	True_Pos
725	1.815	POS	2.512	POS	True_Pos
727	2.293	POS	3.742	POS	True_Pos
728	2.217	POS	3.560	POS	True_Pos
729	1.745	POS	2.621	POS	True_Pos
735	1.729	POS	2.961	POS	True_Pos
748	1.331	POS	1.778	POS	True_Pos
758	1.851	POS	2.552	POS	True_Pos
761	1.831	POS	2.536	POS	True_Pos
763	1.754	POS	1.612	POS	True_Pos
765	1.504	POS	1.816	POS	True_Pos
766	1.501	POS	2.120	POS	True_Pos
771	2.077	POS	3.312	POS	True_Pos
772	1.380	POS	2.000	POS	True_Pos
773	2.010	POS	3.201	POS	True_Pos
778	2.105	POS	3.403	POS	True_Pos
779	1.149	POS	1.721	POS	True_Pos
782	1.601	POS	2.144	POS	True_Pos
786	1.185	POS	1.505	POS	True_Pos
788	2.035	POS	3.010	POS	True_Pos
795	1.923	POS	2.891	POS	True_Pos
796	1.558	POS	2.069	POS	True_Pos
798	1.794	POS	2.382	POS	True_Pos
800	1.517	POS	2.041	POS	True_Pos
801	1.678	POS	2.676	POS	True_Pos

803	1.729	POS	2.515	POS	True_Pos
805	1.679	POS	2.245	POS	True_Pos
807	1.240	POS	1.837	NEG	True_Pos
818	1.586	POS	2.696	POS	True_Pos
821	1.738	POS	3.143	POS	True_Pos
831	1.528	POS	2.336	POS	True_Pos
832	1.386	POS	1.927	POS	True_Pos
837	1.683	POS	2.573	POS	True_Pos
840	1.575	POS	2.936	POS	True_Pos
844	2.082	POS	3.124	POS	True_Pos
846	1.793	POS	2.855	POS	True_Pos
848	1.832	POS	2.741	POS	True_Pos
849	2.059	POS	3.186	POS	True_Pos
853	1.854	POS	2.557	POS	True_Pos
858	1.788	POS	2.858	POS	True_Pos
861	2.121	POS	3.358	POS	True_Pos
862	1.862	POS	3.396	POS	True_Pos
