# Supplementary material for: Validation of new equipment for SARS-CoV-2 diagnosis in Ecuador: Detection of the virus and antibodies generated by disease and vaccines with one POC device
Source: PLoS One. 2025 Apr 16;20(4):e0321794. doi: 10.1371/journal.pone.0321794 (PMC12002511; doi:10.1371/journal.pone.0321794)
Supplement: S7 File — (PDF) [file pone.0321794.s007.pdf]

SAMPLE	PLATE	Spectrophotometer (ABS relative ratio)	SPEC Spike-RBD	PLUM (PRU relative ratio)	PLUM Spike-RBD	VALIDATION
26	PLATE 01C	0.222	NEG	0.198	NEG	True_Neg
64	PLATE 01C	0.189	NEG	0.170	NEG	True_Neg
81	PLATE 01C	0.221	NEG	0.179	NEG	True_Neg
188	PLATE 01C	0.163	NEG	0.181	NEG	True_Neg
250	PLATE 03C	1.240	NEG	1.652	NEG	True_Neg
293	PLATE 01C	0.770	NEG	0.728	NEG	True_Neg
353	PLATE 01C	1.387	NEG	2.004	POS	False_Pos
359	PLATE 01C	0.231	NEG	0.198	NEG	True_Neg
475	PLATE 03C	1.252	NEG	1.257	NEG	True_Neg
489	PLATE 02C	1.322	NEG	1.656	NEG	True_Neg
525	PLATE 02C	0.700	NEG	0.787	NEG	True_Neg
544	PLATE 02C	0.135	NEG	0.102	NEG	True_Neg
563	PLATE 02C	0.900	NEG	1.235	NEG	True_Neg
602	PLATE 02C	0.443	NEG	0.403	NEG	True_Neg
634	PLATE 02C	0.679	NEG	0.724	NEG	True_Neg
736	PLATE 02C	0.238	NEG	0.260	NEG	True_Neg
781	PLATE 02C	0.045	NEG	0.045	NEG	True_Neg
897	PLATE 03C	0.403	NEG	0.432	NEG	True_Neg
864	PLATE 01C	1.977	POS	3.389	POS	True_Pos
865	PLATE 03C	2.597	POS	2.851	POS	True_Pos
866	PLATE 03C	2.592	POS	3.286	POS	True_Pos
867	PLATE 03C	1.441	POS	1.913	POS	True_Pos
868	PLATE 01C	2.207	POS	3.566	POS	True_Pos
869	PLATE 01C	2.249	POS	3.600	POS	True_Pos
870	PLATE 01C	1.542	POS	2.348	POS	True_Pos
871	PLATE 01C	1.822	POS	2.869	POS	True_Pos
872	PLATE 01C	1.825	POS	2.592	POS	True_Pos
873	PLATE 01C	2.078	POS	3.552	POS	True_Pos
874	PLATE 01C	1.918	POS	3.103	POS	True_Pos
875	PLATE 01C	1.951	POS	3.456	POS	True_Pos
876	PLATE 02C	1.976	POS	4.740	POS	True_Pos
877	PLATE 02C	2.130	POS	3.888	POS	True_Pos
878	PLATE 03C	1.842	POS	2.409	POS	True_Pos
879	PLATE 02C	2.207	POS	6.909	POS	True_Pos
880	PLATE 03C	1.753	POS	2.134	POS	True_Pos
881	PLATE 02C	2.268	POS	4.710	POS	True_Pos
882	PLATE 03C	1.753	POS	1.866	POS	True_Pos
883	PLATE 02C	2.106	POS	5.296	POS	True_Pos
884	PLATE 01C	1.984	POS	3.668	POS	True_Pos
885	PLATE 01C	2.308	POS	3.555	POS	True_Pos
886	PLATE 01C	1.757	POS	2.499	POS	True_Pos
887	PLATE 01C	1.885	POS	3.459	POS	True_Pos
888	PLATE 03C	2.073	POS	2.341	POS	True_Pos
889	PLATE 03C	2.492	POS	2.876	POS	True_Pos
890	PLATE 01C	1.892	POS	3.065	POS	True_Pos
891	PLATE 03C	2.260	POS	2.593	POS	True_Pos
892	PLATE 01C	1.677	POS	2.276	POS	True_Pos

893	PLATE 01C	2.167	POS	3.550	POS	True_Pos
894	PLATE 03C	1.535	POS	1.725	POS	True_Pos
895	PLATE 01C	1.603	POS	2.450	POS	True_Pos
896	PLATE 02C	1.921	POS	5.227	POS	True_Pos
898	PLATE 03C	1.692	POS	2.155	POS	True_Pos
899	PLATE 02C	2.354	POS	7.797	POS	True_Pos
900	PLATE 03C	2.296	POS	2.777	POS	True_Pos
901	PLATE 03C	2.608	POS	2.823	POS	True_Pos
902	PLATE 03C	2.348	POS	3.123	POS	True_Pos
903	PLATE 02C	2.067	POS	4.947	POS	True_Pos
904	PLATE 03C	1.735	POS	1.893	POS	True_Pos
905	PLATE 03C	1.896	POS	2.157	POS	True_Pos
906	PLATE 03C	1.632	POS	1.723	POS	True_Pos
907	PLATE 03C	2.344	POS	2.783	POS	True_Pos
908	PLATE 03C	2.331	POS	3.044	POS	True_Pos
