# Supplementary material for: Validation of new equipment for SARS-CoV-2 diagnosis in Ecuador: Detection of the virus and antibodies generated by disease and vaccines with one POC device
Source: PLoS One. 2025 Apr 16;20(4):e0321794. doi: 10.1371/journal.pone.0321794 (PMC12002511; doi:10.1371/journal.pone.0321794)
Supplement: S8 File — (PDF) [file pone.0321794.s008.pdf]

SAMPLE	PLATE	Spectrophotometer (ABS relative ratio)	SPEC Nucleocapsid	PLUM (PRU relative ratio)	PLUM Nucleocapsid	VALIDATION
26	PLATE 01	0.765	NEG	0.739	NEG	True_Neg
64	PLATE 02	0.518	NEG	0.499	NEG	True_Neg
81	PLATE 03	0.337	NEG	0.356	NEG	True_Neg
188	PLATE 05	0.411	NEG	0.373	NEG	True_Neg
250	PLATE 07	0.680	NEG	0.596	NEG	True_Neg
293	PLATE 08	0.324	NEG	0.339	NEG	True_Neg
353	PLATE 10	0.462	NEG	0.566	NEG	True_Neg
359	PLATE 10	0.115	NEG	0.167	NEG	True_Neg
475	PLATE 13	0.129	NEG	0.178	NEG	True_Neg
489	PLATE 13	0.156	NEG	0.184	NEG	True_Neg
525	PLATE 14	0.362	NEG	0.330	NEG	True_Neg
544	PLATE 14	0.346	NEG	0.326	NEG	True_Neg
563	PLATE 15	0.271	NEG	0.306	NEG	True_Neg
602	PLATE 16	0.208	NEG	0.267	NEG	True_Neg
634	PLATE 16	0.205	NEG	0.240	NEG	True_Neg
736	PLATE 19	0.093	NEG	0.191	NEG	True_Neg
781	PLATE 20	0.154	NEG	0.218	NEG	True_Neg
864	PLATE 22	0.435	NEG	0.471	NEG	True_Neg
865	PLATE 28	0.181	NEG	0.198	NEG	True_Neg
866	PLATE 22	0.158	NEG	0.183	NEG	True_Neg
867	PLATE 22	0.087	NEG	0.150	NEG	True_Neg
868	PLATE 28	0.294	NEG	0.246	NEG	True_Neg
869	PLATE 22	0.205	NEG	0.221	NEG	True_Neg
870	PLATE 22	0.227	NEG	0.216	NEG	True_Neg
872	PLATE 22	0.345	NEG	0.255	NEG	True_Neg
873	PLATE 22	0.329	NEG	0.276	NEG	True_Neg
875	PLATE 22	0.297	NEG	0.224	NEG	True_Neg
878	PLATE 22	0.213	NEG	0.206	NEG	True_Neg
879	PLATE 22	0.205	NEG	0.155	NEG	True_Neg
880	PLATE 22	0.794	NEG	0.789	NEG	True_Neg
881	PLATE 22	0.570	NEG	0.466	NEG	True_Neg
882	PLATE 22	0.295	NEG	0.202	NEG	True_Neg
883	PLATE 28	0.183	NEG	0.227	NEG	True_Neg
885	PLATE 22	0.280	NEG	0.216	NEG	True_Neg
887	PLATE 22	0.356	NEG	0.253	NEG	True_Neg
889	PLATE 22	0.517	NEG	0.484	NEG	True_Neg
890	PLATE 22	0.138	NEG	0.132	NEG	True_Neg
891	PLATE 22	0.187	NEG	0.162	NEG	True_Neg
892	PLATE 22	0.460	NEG	0.393	NEG	True_Neg
893	PLATE 28	0.511	NEG	0.603	NEG	True_Neg
894	PLATE 22	0.196	NEG	0.156	NEG	True_Neg
895	PLATE 22	0.498	NEG	0.426	NEG	True_Neg
896	PLATE 22	0.409	NEG	0.328	NEG	True_Neg
897	PLATE 22	0.143	NEG	0.103	NEG	True_Neg
900	PLATE 28	0.450	NEG	0.498	NEG	True_Neg
901	PLATE 22	0.115	NEG	0.096	NEG	True_Neg
906	PLATE 23	0.515	NEG	0.584	NEG	True_Neg

907	PLATE 23	0.030	NEG	0.105	NEG	True_Neg
871	PLATE 22	0.870	POS	0.865	POS	True_Pos
874	PLATE 22	1.054	POS	1.033	POS	True_Pos
876	PLATE 22	0.921	POS	0.878	POS	True_Pos
877	PLATE 22	1.992	POS	2.515	POS	True_Pos
884	PLATE 22	0.991	POS	0.956	POS	True_Pos
886	PLATE 22	1.207	POS	1.184	POS	True_Pos
888	PLATE 22	1.060	POS	1.102	POS	True_Pos
898	PLATE 22	0.867	POS	0.820	POS	True_Pos
899	PLATE 22	1.043	POS	1.109	POS	True_Pos
902	PLATE 26	1.237	POS	1.350	POS	True_Pos
903	PLATE 23	1.031	POS	0.891	POS	True_Pos
904	PLATE 23	0.953	POS	1.162	POS	True_Pos
905	PLATE 23	1.903	POS	2.364	POS	True_Pos
908	PLATE 23	1.470	POS	0.427	NEG	False_Neg
