# Supplementary material for: Validation of new equipment for SARS-CoV-2 diagnosis in Ecuador: Detection of the virus and antibodies generated by disease and vaccines with one POC device
Source: PLoS One. 2025 Apr 16;20(4):e0321794. doi: 10.1371/journal.pone.0321794 (PMC12002511; doi:10.1371/journal.pone.0321794)
Supplement: S9 File — (PDF) [file pone.0321794.s009.pdf]

SAMPLE	SPEC AVERAGE S-RBD-N	SPEC AVERAGE	PLUM AVERAGE S-RBD-N	PLUM AVERAGE	FINAL VALIDATION
26	0.493	True_Neg	0.468	True_Neg	True_Neg
64	0.353	True_Neg	0.335	True_Neg	True_Neg
81	0.279	True_Neg	0.267	True_Neg	True_Neg
188	0.287	True_Neg	0.277	True_Neg	True_Neg
250	0.960	True_Neg	1.124	True_Neg	True_Neg
293	0.547	True_Neg	0.533	True_Neg	True_Neg
353	0.924	True_Neg	2.004	S-RBD	False_Pos
359	0.173	True_Neg	0.182	True_Neg	True_Neg
475	0.691	True_Neg	0.718	True_Neg	True_Neg
489	0.739	True_Neg	0.920	True_Neg	True_Neg
525	0.531	True_Neg	0.559	True_Neg	True_Neg
544	0.240	True_Neg	0.214	True_Neg	True_Neg
563	0.585	True_Neg	0.770	True_Neg	True_Neg
602	0.325	True_Neg	0.335	True_Neg	True_Neg
634	0.442	True_Neg	0.482	True_Neg	True_Neg
736	0.165	True_Neg	0.225	True_Neg	True_Neg
781	0.099	True_Neg	0.131	True_Neg	True_Neg
897	0.273	True_Neg	0.268	True_Neg	True_Neg
871	1.346	True_Pos	1.867	True_Pos	True_Pos
874	1.486	True_Pos	2.068	True_Pos	True_Pos
876	1.449	True_Pos	2.809	True_Pos	True_Pos
877	2.061	True_Pos	3.201	True_Pos	True_Pos
884	1.487	True_Pos	2.312	True_Pos	True_Pos
886	1.482	True_Pos	1.841	True_Pos	True_Pos
888	1.567	True_Pos	1.721	True_Pos	True_Pos
898	1.279	True_Pos	1.487	True_Pos	True_Pos
899	1.698	True_Pos	4.453	True_Pos	True_Pos
902	1.792	True_Pos	2.237	True_Pos	True_Pos
903	1.549	True_Pos	2.919	True_Pos	True_Pos
904	1.344	True_Pos	1.528	True_Pos	True_Pos
905	1.900	True_Pos	2.260	True_Pos	True_Pos
908	1.901	True_Pos	0.927	S-RBD	False_Neg
